# Extracellular vesicles of Norway spruce contain precursors and enzymes for lignin formation and salicylic acid

**DOI:** 10.1093/plphys/kiae287

**Published:** 2024-05-21

**Authors:** Santeri Kankaanpää, Enni Väisänen, Geert Goeminne, Rabah Soliymani, Sandrien Desmet, Anatoliy Samoylenko, Seppo Vainio, Gunnar Wingsle, Wout Boerjan, Ruben Vanholme, Anna Kärkönen

**Affiliations:** Production Systems, Natural Resources Institute Finland (Luke), 31600 Jokioinen, Finland; Organismal and Evolutionary Biology Research Programme, Faculty of Biological and Environmental Sciences, Viikki Plant Science Centre, University of Helsinki, 00014 Helsinki, Finland; VIB Metabolomics Core Ghent, VIB-UGent Center for Plant Systems Biology, Ghent University, 9052 Ghent, Belgium; Meilahti Clinical Proteomics Core Facility, Biochemistry & Developmental Biology, Faculty of Medicine, Biomedicum Helsinki, University of Helsinki, 00014 Helsinki, Finland; VIB Metabolomics Core Ghent, VIB-UGent Center for Plant Systems Biology, Ghent University, 9052 Ghent, Belgium; Faculty of Biochemistry and Molecular Medicine, Disease Networks Research Unit, Kvantum Institute, Infotech Oulu, University of Oulu, 90014 Oulu, Finland; Faculty of Biochemistry and Molecular Medicine, Disease Networks Research Unit, Kvantum Institute, Infotech Oulu, University of Oulu, 90014 Oulu, Finland; Umeå Plant Science Centre, Department of Forest Genetics and Plant Physiology, Swedish University of Agricultural Sciences, 90183 Umeå, Sweden; VIB Center for Plant Systems Biology, VIB, 9052 Ghent, Belgium; Department of Plant Biotechnology and Bioinformatics, Ghent University, 9052 Ghent, Belgium; VIB Center for Plant Systems Biology, VIB, 9052 Ghent, Belgium; Department of Plant Biotechnology and Bioinformatics, Ghent University, 9052 Ghent, Belgium; Production Systems, Natural Resources Institute Finland (Luke), 00790 Helsinki, Finland; Department of Agricultural Sciences, Viikki Plant Science Centre, University of Helsinki, 00014 Helsinki, Finland

## Abstract

Lignin is a phenolic polymer in plants that rigidifies the cell walls of water-conducting tracheary elements and support-providing fibers and stone cells. Different mechanisms have been suggested for the transport of lignin precursors to the site of lignification in the cell wall. Extracellular vesicle (EV)-enriched samples isolated from a lignin-forming cell suspension culture of Norway spruce (*Picea abies* L. Karst.) contained both phenolic metabolites and enzymes related to lignin biosynthesis. Metabolomic analysis revealed mono-, di-, and oligolignols in the EV isolates, as well as carbohydrates and amino acids. In addition, salicylic acid (SA) and some proteins involved in SA signaling were detected in the EV-enriched samples. A proteomic analysis detected several laccases, peroxidases, β-glucosidases, putative dirigent proteins, and cell wall-modifying enzymes, such as glycosyl hydrolases, transglucosylase/hydrolases, and expansins in EVs. Our findings suggest that EVs are involved in transporting enzymes required for lignin polymerization in Norway spruce, and radical coupling of monolignols can occur in these vesicles.

## Teaser

Extracellular vesicles transport enzymes putatively involved in lignin polymerization in Norway spruce, and radical coupling of monolignols can occur in these vesicles.

## Introduction

Lignin is the second-most abundant terrestrial biopolymer after cellulose, constituting 20% to 35% of the dry weight of wood. It gives strength and waterproofs the cell walls of water-conducting vessels and tracheids, as well as support-giving sclerenchyma cells (fibers and stone cells). In gymnosperms, such as Norway spruce (*Picea abies* L. Karst.), lignin is primarily composed of guaiacyl (G) units with small amounts of *p*-hydroxyphenyl (H) units, which are derived from coniferyl and *p*-coumaryl alcohols, respectively (Boerjan et al. 2003; Vanholme et al. 2019; del Río et al. 2020). In addition to these canonical monolignols, other units such as cinnamaldehydes, are commonly present in spruce lignin (Yamamoto et al. 2020).

The biosynthesis of monolignols occurs in the cytosol and is quite well understood (reviewed in Dixon and Barros 2019; Vanholme et al. 2019; and De Meester et al. 2022). The final polymerization takes place in the cell wall via laccase- and/or peroxidase-catalyzed oxidation of monolignols to form phenolic radicals, which then couple nonenzymatically (Berthet et al. 2011; Zhao et al. 2013; Shigeto and Tsutsumi 2016; Hoffmann et al. 2020, 2022; Rojas-Murcia et al. 2020; Blaschek et al. 2023). There are still many open questions in relation to the transport of lignin precursors to the cell wall (Perkins et al. 2019). Vesicular transport has been suggested as a means of transport (Stafford 1974) supported by the observations that radioactivity from labeled phenylalanine and cinnamic acid was localized in the ER, Golgi bodies, Golgi vesicles, and cell walls of coleoptiles of wheat (*Triticum aestivum*) (Pickett-Heaps 1968) and cryptomeria (*Cryptomeria japonica*) (Takabe et al. 1985). However, experiments with seedlings of lodgepole pine (*Pinus contorta* var. *latifolia*) showed that the Golgi is not likely involved in the monolignol transport (Kaneda et al. 2008). Using membrane vesicles prepared from the rosette leaves of Arabidopsis (*Arabidopsis thaliana*), the ATP-binding cassette (ABC)-transporter-mediated transport of coniferyl alcohol has been detected (Miao and Liu 2010). In the plasma membrane of the endodermal and vascular tissue of Arabidopsis roots, a G-type ABC transporter, called ABCG29, was identified, which is capable of transporting *p*-coumaryl alcohol in vitro (Alejandro et al. 2012). However, no transporters for the more abundant lignin building blocks, coniferyl and sinapyl alcohol, have yet been found. Furthermore, several angiosperm and gymnosperm tree species are evidently capable of the secondary active, H^+^-antiporter-dependent transport of coniferin and *p*-coumaryl alcohol glucoside through the endomembrane system and/or via the tonoplast in vitro, suggesting that vesicular transport may be involved in monolignol glucoside secretion (Tsuyama et al. 2013, 2019; Väisänen et al. 2020; Shimada et al. 2021). A similar type of transport was also detected in Bright Yellow-2 (BY-2) cells of tobacco (*Nicotiana tabacum*), which do not lignify (Väisänen et al. 2020), raising some doubt that this pathway is specifically involved in lignification. Biochemical and computational studies have shown that the diffusion of monolignols and dimers, but not glycosidic forms, is another possible mechanism for the secretion of lignin precursors across the plasma membrane (Boija and Johansson 2006; Vermaas et al. 2019). Recently, Perkins et al. (2022) showed that the entrapment of a monolignol-oxidizing enzyme in liposomes can create a sink that facilitates the diffusion of monolignols across the membrane following a polymerization-driven concentration gradient.

Extracellular vesicles (EVs) are lipid bilayer-enclosed particles that are involved in the secretion of lipids, proteins, metabolites, and nucleic acids. They can deliver information from one cell to another (Woith et al. 2019) and are considered to be one of several mechanisms of unconventional protein secretion (Ding et al. 2014; Maricchiolo et al. 2022). EVs are derived from the endosomal pathway, by directly blebbing from the plasma membrane or from apoptotic cells (van Niel et al. 2018). In addition, there is an exocyst positive organelle secretion (EXPO) mechanism, which has been shown to function as an efficient mediator of EV secretion in plants (Wang et al. 2010; Ding et al. 2014). The mechanisms underpinning the biogenesis and secretion of EVs have been well characterized in mammalian cells (reviewed by Colombo et al. 2014 and Teng and Fussenegger 2021). However, studies on plant EVs have only recently started to emerge, even though EVs were first observed by electron microscopy over 50 yr ago (Halperin and Jensen 1967). Among the key questions for vesicle biogenesis in plants is the contribution of endosomal sorting complexes required for transport (ESCRT) in the formation of multivesicular bodies, which consequently release exosomes. ESCRTs have also been shown to function in the blebbing of microvesicles from the plasma membrane (Hurley 2015; Gao et al. 2017).

The majority of plant EV research to date has focused on plant–pathogen interactions (Regente et al. 2017; De Palma et al. 2020; He et al. 2021). The interest in these interactions has been partly due to the identification of penetration resistance proteins, such as PENETRATION3 (PEN3), which is an abundant protein in Arabidopsis EVs and has been shown to play a role in pathogen resistance (Stein et al. 2006; Rutter and Innes 2017). EVs also participate in pollen tube growth (Prado et al. 2014), wound repair (Liu et al. 2020a), and cell wall synthesis (Regente et al. 2017; de la Canal and Pinedo 2018) and are present in root exudates (De Palma et al. 2020) and in the culture medium of plant cell suspensions (Woith et al. 2021). Recently, De Bellis et al. (2022) used electron microscopy to show that EVs are likely involved in the transport of suberin precursors to the cell wall in the endodermis of Arabidopsis roots. Furthermore, secretory autophagy was shown to be required for pathogen-induced lignin deposition (Jeon et al. 2022). In Arabidopsis mutants with defective autophagy, the formation of a pathogen-induced lignin barrier was impaired. Moreover, EV-like structures containing monolignols colocalize with autophagic vesicles deployed to the site of defense-induced lignification (Jeon et al. 2022).

Here, we examined the culture medium of an extracellular lignin-forming cell culture of Norway spruce (Simola et al. 1992; Kärkönen et al. 2002) for the presence of EVs. In this cell culture, the extracellular lignin that forms in the culture medium is mostly composed of G units with a slightly elevated content of H units as compared to vertical wood lignin (Brunow et al. 1993; Giummarella et al. 2019). Our hypothesis was that EVs secreted by cultured spruce cells transport into the apoplast lignin precursors and/or proteins involved in lignin biosynthesis. With suspension cell cultures, the culture medium is part of the continuum of apoplastic fluid that permeates cell walls. The vesicles isolated from the medium are natively secreted by cells, and not those which could be released when cellular membranes are ruptured during tissue homogenization. Previously, it was shown that there is only little cell death during the cultivation of these spruce cell cultures because peroxidase isoenzyme patterns clearly differ between the culture medium and other cellular compartments (Kärkönen et al. 2002), and flavonoids were detected only intracellularly and not in the culture medium (Laitinen et al. 2017). In addition to lignin-forming conditions, we also examined EVs isolated from the culture medium when lignin polymerization was hindered by the KI-mediated scavenging of H_2_O_2_ (Huwiler et al. 1985; Laitinen et al. 2017). By using proteomic and metabolomic analyses of the EV-enriched samples isolated from the culture medium of tissue-cultured cells, we show that spruce cells secrete EVs that carry both enzymes and precursors involved in lignin formation. Furthermore, our data show that EV-enriched samples contain lignin monomers, dimers, and oligomers, suggesting that the polymerization of monolignols can occur in the EV lumen.

## Results and discussion

### EVs are present in the spruce cell culture medium

**Figure 1.**
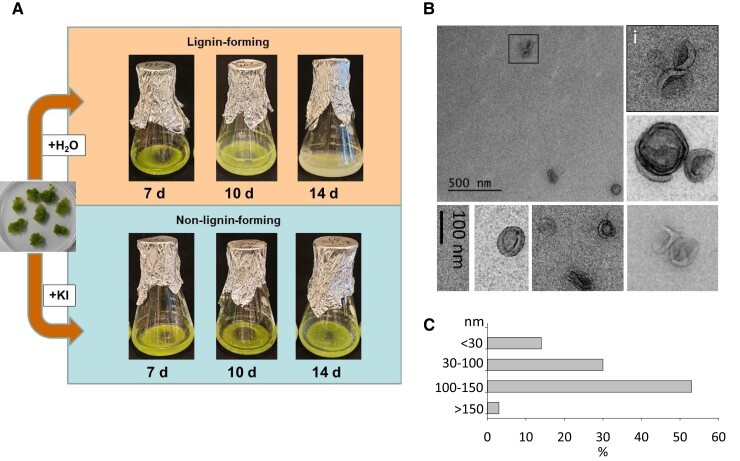
Tissue culture model and extracellular vesicles (EVs) isolated from the culture medium of tissue-cultured cells of Norway spruce. **A)** Setup for experiments using an extracellular lignin-forming Norway spruce cell culture (according to Laitinen et al. 2017 with some modifications). Spruce cells were transferred at Day 0 from the solid maintenance medium to the liquid nutrient medium supplemented either with an H_2_O_2_ scavenger, KI (5 mm; non-lignin-forming conditions), or the corresponding volume of water (H_2_O; lignin-forming conditions). At Days 7, 10, and 14, 4 replicate cultures of each treatment were harvested, and the media were collected for the isolation of extracellular vesicles (EVs) (see Materials and methods). **B)** Transmission electron micrographs of the EVs pelleted with ultracentrifugation from the spruce cell culture media. Common characteristics of EVs are seen in the EM images with moon-shaped particles with membrane characteristics shown in the close-ups. i) Close-up of the EVs marked with a rectangular shape in the big image. A scale bar (100 nm) shown is for all close-up images. **C)** Histogram of the size distribution of EVs as measured from the transmission electron microscopy images using ImageJ (*n* = 100).

A cell culture of Norway spruce that forms extracellular lignin (Simola et al. 1992; Kärkönen et al. 2002) was used to investigate whether or not spruce cells secrete EVs during lignification. Lignin-forming conditions were compared to those where lignin formation was hindered by the addition of KI, an H_2_O_2_ scavenger, to evaluate the effects on EV presence and cargo (Fig. 1A; based on Laitinen et al. 2017). Extracellular lignin became visible in the culture medium of lignin-forming cultures 10 d following the transfer of callus cells into the liquid medium. At Day 14, the medium was fully cloudy with extracellular lignin (Fig. 1A). In contrast, the KI-treated cultures remained clear until Day 14 when some cloudiness appeared in the medium, suggesting that some extracellular lignin had formed.

Our data show that cultured Norway spruce cells secrete EVs into the culture medium (Fig. 1B). The EV-enriched samples (henceforth referred to as EV samples) were isolated from the culture media of both lignin-forming and non-lignin-forming cell cultures using an ultracentrifugation-based approach (Supplementary Fig. S1). There was a gel-like ribbon in the EV pellet surrounding the membranous core, suggesting that some pectin had pelleted with EVs. This was then confirmed through ruthenium red staining and an immunoblot analysis with an LM19 antibody that recognizes weakly methylesterified homogalacturonan (Supplementary Fig. S2, A to C). The immunoblot showed that a majority of pectin was extracted away from the pellets using an EDTA-containing buffer (Supplementary Fig. S2C). Examination of the resuspended pellets by transmission electron microscopy (TEM) revealed that 53% of the EVs had a diameter of 30 to 100 nm, 30% of 100 to 150 nm, 14% over 150 nm, and only 3% less than 30 nm (Fig. 1, B and C). The heterogeneity in the EV sizes has also been observed earlier, for example, in Arabidopsis (Rutter and Innes 2017; Huang et al. 2021), sunflower (*Helianthus annuus*; Regente et al. 2017), and sorghum (*Sorghum bicolor*; Chaya et al 2023). Results from the nanoparticle tracking analysis (NTA) of the particle concentration (1.04 × 10^12^ isolated from 18 mL of the medium) and size distribution (70 to 800 nm) (Supplementary Fig. S2D), however, did not fully correspond to the observations made via TEM imaging. No large particles were seen by TEM, and the particle concentration was much lower than was indicated by the NTA results. The NTA is known to overestimate the average size of EVs as it is not capable of measuring particles under 60 nm in diameter (Gardiner et al. 2013; Maas et al. 2015; Bachurski et al. 2019). Furthermore, the refractive index of the EVs can differ substantially from the polystyrene beads used to prepare the calibration curves (Gardiner et al. 2014).

### EV metabolome contains mono-, di-, tri-, and tetralignols

**Figure 2.**
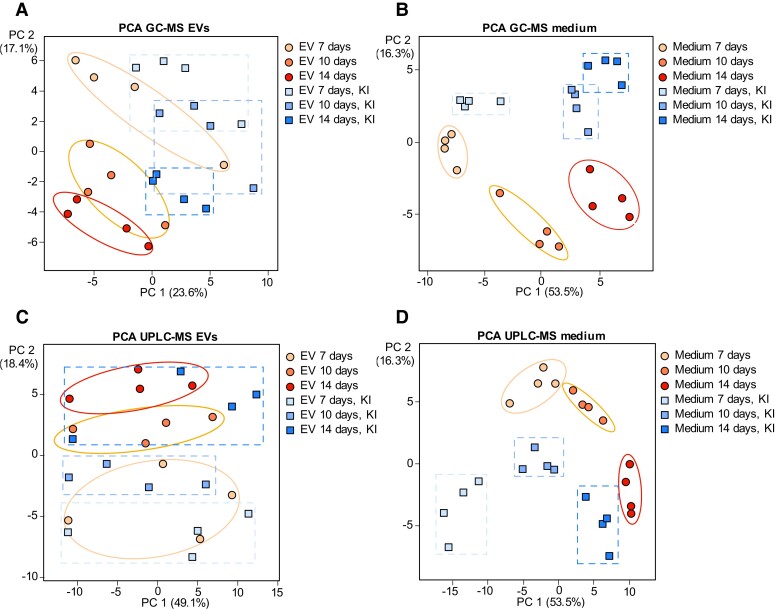
Metabolomic analysis of compounds in the extracellular vesicle (EV) samples and in the EV-depleted culture media of Norway spruce cultured cells. Principal component analysis (PCA) plot of the gas chromatography–mass spectrometry (GC–MS)-detected compounds in **A)** the EV samples and in **B)** the culture media at 3 different time points (7, 10, and 14 d) for 2 treatments, namely, lignin-forming and non-lignin-forming (KI-treatment). Ellipses and rectangles frame biological repeats and are added for clarity. The value between the brackets is the percentage of the variation explained by the respective principal component (PC). PCA plot of the ultra-performance liquid chromatography–mass spectrometry (UPLC–MS)-detected compounds in **C)** the EV samples and in **D)** the EV-depleted culture media in 3 different time points (7, 10, and 14 d) in 2 treatments [lignin-forming and non-lignin-forming (KI-treatment)]. Ellipses and rectangles frame biological repeats and are added for clarity. The value between the brackets is the percentage of the variation explained by the respective principal component (PC).

**Figure 3.**
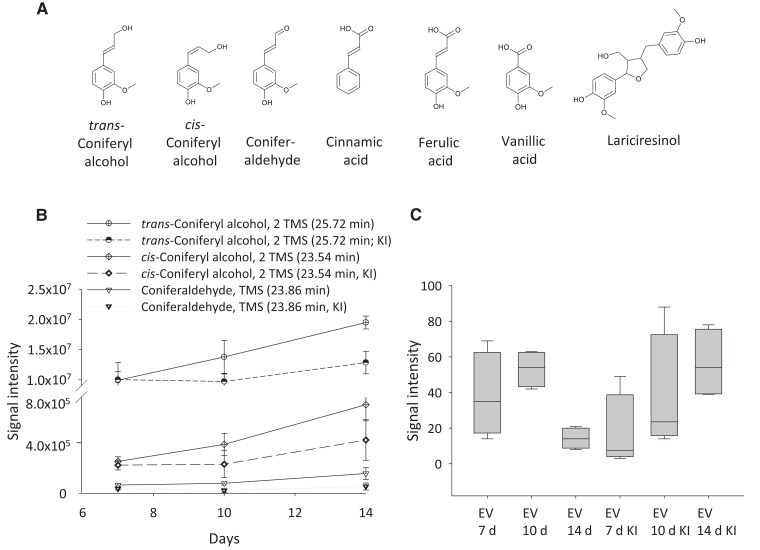
Accumulation of the phenolic metabolites in the extracellular vesicle (EV) samples as observed in the metabolic analysis. **A)** Example of the phenolic metabolites detected only in the EV samples, and not in the medium samples, by gas chromatography–mass spectrometry (GC–MS). **B)** Accumulation of coniferyl alcohol and coniferaldehyde in the EV samples as detected by GC–MS in lignin-forming and non-lignin-forming (KI-treatment) conditions (mean ± SD, *n* = 4). **C)** Accumulation of G(8-8)G hexoside (34, pinoresinol hexoside) in the EV samples as observed by ultra-performance liquid chromatography–mass spectrometry (UPLC–MS) in lignin-forming and non-lignin-forming conditions (KI-treatment). The boxplots show the median (horizontal lines), 25th to 75th percentiles (edges of the boxes), and minimum to maximum values (edges of the whiskers) (*n* = 4).

For each timepoint and treatment, 4 biological replicates of the EV samples were analyzed by gas chromatography–mass spectrometry (GC–MS) and ultra-performance liquid chromatography–mass spectrometry (UPLC–MS) to investigate the metabolic cargo (Fig. 2). The culture media in which EVs had been pelleted away (i.e. EV-depleted media, henceforth referred to as medium/media) were used for comparative purposes. The GC–MS data showed that the metabolomes of the EV samples (Supplementary Table S1A) were partly different from those of the corresponding media (Supplementary Table S1B). In addition, the metabolites changed in both EVs and in the media during the progression of the lignin formation (Fig. 2, A and B). The EV samples contained amino acids (e.g. serine and threonine), sugars (e.g. pinitol and cellobiose), fatty acids, and various phenolic compounds (Supplementary Table S1). Interestingly, coniferyl alcohol, coniferaldehyde, cinnamic acid, ferulic acid, and the lignan lariciresinol were among the compounds detected in the EV samples, but not in the culture media (Fig. 3A; Supplementary Fig. S3; Supplementary Table S1). This observation hints at the specificity of the EV cargo. The content of coniferyl alcohol increased in the EV samples isolated from the lignin-forming cultures throughout the progression of the lignin formation (Fig. 3B). In addition, in the non-lignin-forming conditions (KI-treatment), the coniferyl alcohol levels were slightly elevated at Day 14 in comparison with those at Days 7 and 10. The coniferaldehyde levels also increased throughout the culturing period, especially in the lignin-forming cultures (Fig. 3B).

**Figure 4.**
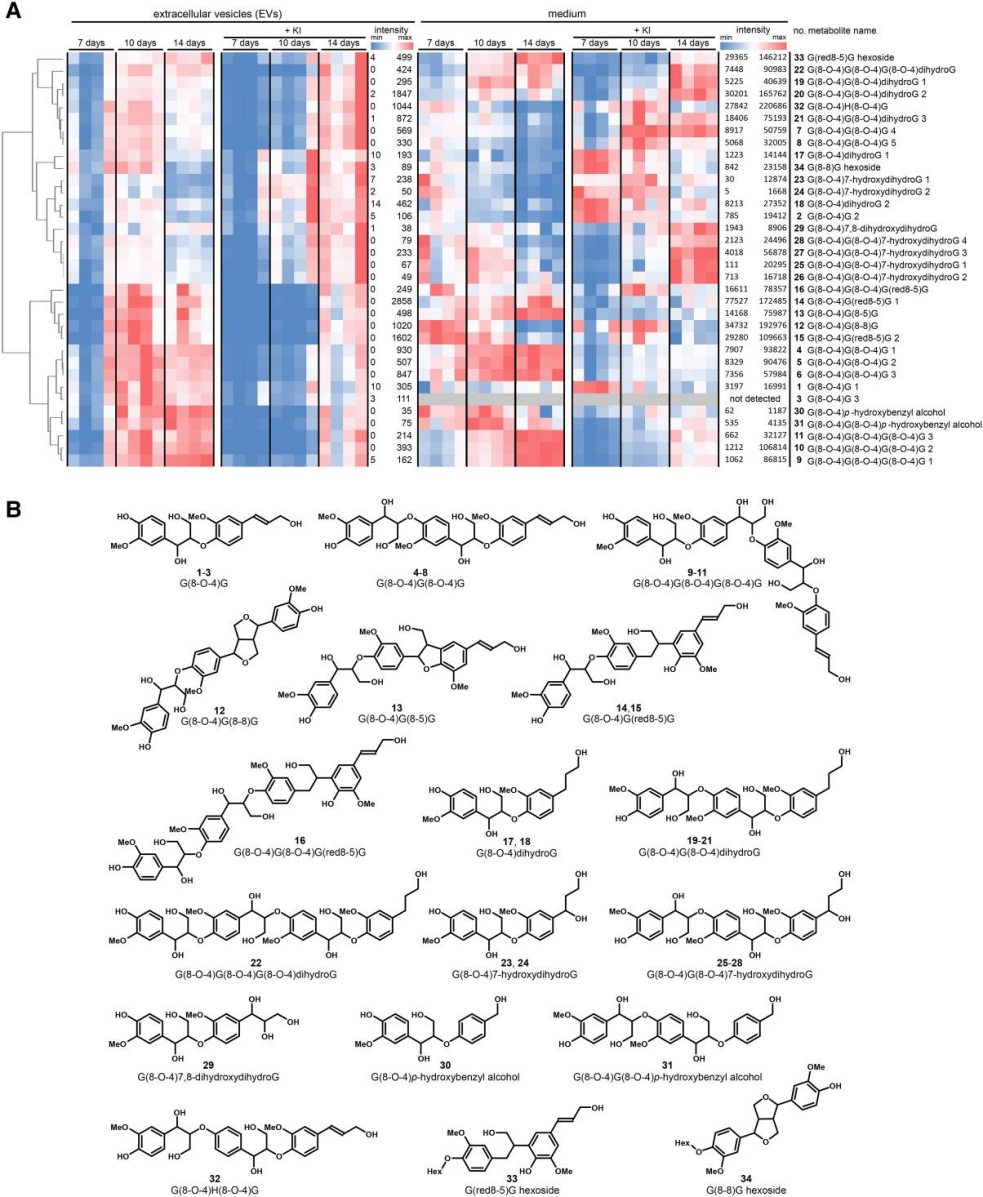
Compounds detected by ultra-performance liquid chromatography–mass spectrometry (UPLC–MS) analysis in the extracellular vesicle (EV) samples and in the EV-depleted culture media of Norway spruce cultured cells. **A)** Heat maps showing compounds detected in the EV samples and in the culture media. The structural characterizations were based on the MS/MS-fragmentation spectra. **B)** Structural formulae of di- and oligolignols detected in the EV samples of Norway spruce.

Using UPLC–MS, various di- and oligolignols and dilignol glycosides were identified both in the EV samples and in the media; these compounds differed between lignin-forming and non-lignin-forming conditions and the amounts changed over time (Fig. 2, C and D; Fig. 4A; Supplementary Table S2). Because of the relatively low abundance of EVs in the culture, the EV samples were much less concentrated than the corresponding medium samples. This difference in the concentrations complicated the comparative analyses. Although the EV pellets were thoroughly washed to remove the medium, we cannot rule out the possibility that some medium remained in the EV samples. In that case, the compounds detected in the EV samples could potentially have originated from the medium instead of the EVs themselves. Because the volume of EVs in each sample was small as compared to the total volume of the medium, we reasoned that the reverse (i.e. contamination of medium samples with EVs) would be negligible, especially since at least a majority of the EVs had been pelleted away from the culture medium. Although all EV samples were prepared in the same way, the proportion of residual culture medium could differ between samples due to technical variations. We assessed the potential for residual medium in the EV samples using 2 complementary strategies: (i) by determining the Pearson correlation coefficients of the metabolite MS peak intensities in the EV samples with the peak intensities in the corresponding medium samples; and (ii) by using the intensity of the metabolite MS peak in the EV sample that corresponds to the most intense peak in the medium samples (Supplementary Fig. S4). First, the Pearson correlation coefficients showed that residual medium was negligible for about half of the samples analyzed (7 d samples 1 to 3, 7 d + KI samples 1 to 4, 10 d + KI samples 1 to 4; Supplementary Fig. S4). For the other EV samples, a contamination cannot be ruled out. Second, we evaluated the potential contamination of the EV sample via the metabolite with the most intense peak in the corresponding medium sample. Depending on the sample, this was either G(8-O-4)G(8-8)G (**12**), G(8-O-4)G(red8-5)G 1 (**14**), G(8-O-4)G(8-O-4)dihydroG 2 (**20**), G(8-O-4)H(8-O-4)G (**32**), or G(red8-5)G hexoside (**33**; Fig. 4, A and B). In the same EV samples, which had a low correlation coefficient with their corresponding medium samples (i.e. 7 d samples 1 to 3, 7 d + KI samples 1 to 4, 10 d + KI samples 1 to 4), the corresponding peaks were not detected. This corroborated the first correlation-based approach and confirmed that contamination was minimal in those samples. However, contamination from the medium cannot be excluded for the remaining samples. For these samples, we next calculated which compounds were relatively more abundant in the EV samples as compared to the largest peak in the corresponding medium sample (Supplementary Fig. S4). The number of metabolites that were relatively more abundant in the EV samples differed from sample to sample and varied between 13 (for 7 d sample 4) and 3 (for 10 d sample 3). Three dilignols were more abundant in all EV samples [i.e. G(8-O-4)G 1 (**1**), G(8-O-4)G 3 (**3**), and G(8-O-4)dihydroG 1 (**17**)]. In addition, G(8-O-4)7-hydroxydihydroG 2 (**24**) was more abundant in most EV samples. In summary, in spite of a possible contamination from the medium for some samples, we found that several monolignol dimers (**1**, **3**, **17**, and **24**) were relatively more abundant in the EV samples compared to the corresponding medium samples.

In the EV samples isolated from the lignin-forming cultures, oligolignols were identified, which were differentially present in a treatment/timepoint-dependent manner and had the highest concentration either at Day 10 or 14 following the transfer of cells into the liquid medium (Fig. 4A; Supplementary Table S2). By contrast, the concentration of oligolignols peaked only at Day 14 in the non-lignin-forming conditions. This observation indicates that monolignol coupling was efficiently inhibited by KI, and this inhibition was mitigated over time. Note that the concentration of oligolignols is the result of a balance between the rate of biosynthesis and the rate of subsequent coupling to higher-order oligomers. The accumulation of a dimer could thus either signify an increased biosynthesis, a reduced further coupling, or a combination of both these factors. The main linkage type of the identified oligolignols was 8–O–4, which is the most common linkage in lignin, but 8–8 and 8–5 linkages were also present. Oligolignols in the EV samples contained G and H units and also units originating from dihydroconiferyl alcohol (dihydroG, hydroxydihydroG, dihydroxydihydroG) and *p*-hydroxybenzyl alcohol (Fig. 4B). Reduced structures, such as isodihydrodehydrodiconiferyl alcohol [IDDDC, G(red8-5)G], were present in tri- and tetralignols (**14**, **15**, and **16**). In poplar, IDDDC is formed from dehydrodiconiferyl alcohol [G(8-5)G] by a phenylcoumaran benzylic ether reductase, which is located in the cytosol (Niculaes et al. 2014). G(8-O-4)IDDDC and IDDDC hexoside were also previously identified in the cell culture medium of Norway spruce (Laitinen et al. 2017). It is possible that EVs are responsible for transporting these reduced compounds out from the cytosol. One dilignol, called G(8-O-4)G 3 (**3**), was detected only in the EV samples and not in the media (Fig. 4; Supplementary Table S2). While this could potentially support the hypothesis of specific loading of EVs, it could also be a technical artifact due to ion suppression resulting from co-eluting metabolites in the concentrated medium samples.

The concentration of G(8-8)G hexoside (**34**, pinoresinol hexoside) in the EV samples was decreased by Day 14 in lignin-forming conditions (Fig. 3C; Supplementary Table S2) when the concentrations of several trilignols were still high (Fig. 4A). Accordingly, it is possible that G(8-8)G hexoside was deglycosylated by a β-glucosidase and underwent further coupling to form a trimer. In the proteomic data of the EV samples, 6 proteins were annotated as β-glucosidases (Supplementary Table S3, see below). However, these had low (≤52%) sequence identity to a pine (*Pinus contorta*) β-glucosidase that was previously shown to be involved in coniferin deglucosylation (Dharmawardhana et al. 1999). Diphenol glucosides were, however, not tested as substrates for the coniferin-hydrolyzing enzyme from pine.

### Salicylic acid (SA) was detected in the EV samples

**Figure 5.**
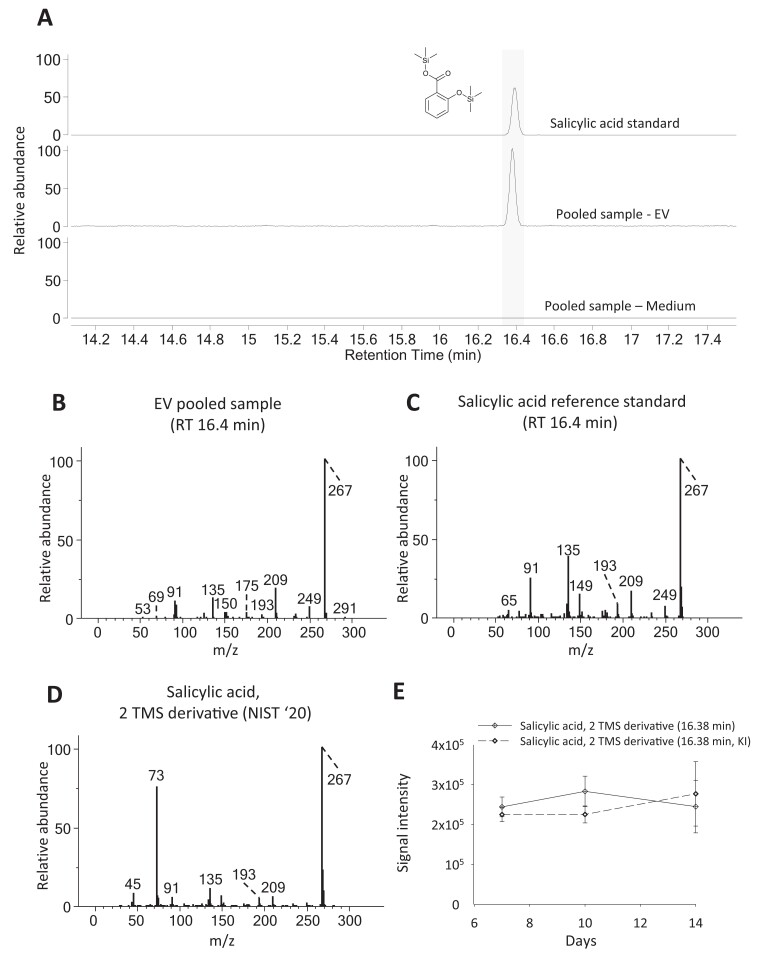
Extracted ion chromatograms (EIC) and electron impact (EI) spectra of salicylic acid (SA) and presence of SA in the extracellular vesicle (EV) samples of Norway spruce. **A)** EIC of *m/z* 267.0869 of a SA reference standard (top), a pooled sample of all EV samples (middle), and a pooled sample of all EV-depleted culture medium samples (bottom). **B)** Deconvoluted EI spectrum of the peak eluting at 16.38 min in a pooled sample of all EV samples. **C)** Deconvoluted EI spectrum of the peak eluting at 16.39 min in the chromatogram of the SA reference standard. **D)** EI spectrum of the 2 trimethylsilyl (TMS) derivative of SA in the National Institute of Standards and Technology (NIST) ‘20 spectral library. **E)** Accumulation of SA in the EV samples as detected by gas chromatography–mass spectrometry (GC–MS) in lignin-forming and non-lignin-forming (KI-treatment) conditions (mean ± SD, *n* = 4).

Salicylic acid (SA) was detected in the EV samples at approximately equal levels in all time points in both treatments (Fig. 5; Supplementary Fig. S5), but remained under the detection limit in the medium samples (Supplementary Table S1). This observation is of interest since exogenous SA treatment has been shown to induce EV production in *Arabidopsis* (Rutter and Innes 2017). SA regulates growth and development in plants and is important for resistance against abiotic stresses and in plant–microbe interactions (reviewed in Bonnemain et al. 2013, Maruri-López et al. 2019). After initial defense responses, plants develop a systemic acquired resistance (SAR), even at sites distal to the initial infection. SA, together with other metabolites (e.g. azelaic acid and glycerol-3-phosphate), is essential for this priming process (Maruri-López et al. 2019). The long-distance transport of SA is known to occur in phloem (e.g. Bonnemain et al. 2013; Lim et al. 2016), but the molecular mechanisms of the SA transport remain obscure. Pathogen infection causes SA levels to increase, and SA is translocated to the apoplast where it is then transported to phloem (Rocher et al. 2009; Lim et al. 2016, 2020). The pKa of the carboxyl group of SA is ∼3.0; hence, this group is primarily deprotonated in the cytosol (pH ∼7 to 7.5) and in the apoplast (pH ∼4.5 to 5) (Bonnemain et al. 2013; Lim et al. 2020). It has been suggested that the transport of SA to and from the apoplast occurs in a pH-dependent carrier system (Rocher et al. 2009; Lim et al. 2016, 2020). It is intriguing that SA transport into the apoplast apparently also occurs via EVs. This observation raises questions about the mechanisms of SA loading into EVs (see discussion below).

### Proteome of Norway spruce EV samples contains cargo proteins involved in lignin and cell wall formation

A proteomic analysis of Norway spruce EV samples was conducted using LC–MS/MS protein identifications. The EV samples were analyzed both with and without EDTA extraction. The first analysis provided information about soluble proteins present in the EV lumen and those bound to pectin that was sloughed off from the cell wall into the culture medium and co-pelleted with the EVs (Supplementary Table S3A). Next, to eliminate pectin and pectin-bound proteins, we extracted EV pellets with EDTA in PBS before the trypsin digestion. To ensure membrane protein solubilization, the sodium deoxycholate detergent concentration was increased in this extraction protocol. The extraction with EDTA reduced the sizes of the pellets and efficiently eliminated pectin, as shown by an immunoblot with the LM19 antibody that recognizes weakly methylesterified homogalacturonan (Supplementary Fig. S2, A to C).

In total, 557 proteins were identified across all the EDTA-extracted EV samples (Supplementary Table S3B). Note that due to the large size and long repetitive sequences, the available ver. 1.0 genome assembly of Norway spruce contains partial gene sequences and gene models with missing exons in addition to full-length correct gene models (Nystedt et al. 2013). This affects the number of unique peptides obtained in the proteomic analysis. Relatively few differences occurred in the proteins that were detected in EVs in the 2 treatments or different time points; thus, all annotated proteins were analyzed as 1 group in Gene Ontology (GO) category enrichment testing. The GO analysis for biological function showed that proteins involved in several metabolic processes were present in the EV samples; terms “cell wall organization and biogenesis” and “response to stress or stimuli” were also enriched (Supplementary Fig. S6A). The GO analysis for molecular function showed that proteins with various enzymatic activities were present in the EV samples, including hydrolases, transferases, and oxidoreductases (Supplementary Fig. S6B). The GO category enrichment for cellular component revealed several categories, 5 of which correspond to the “apoplast.” In addition, categories, such as “membrane-bounded organelle,” “Golgi-associated vesicles,” “Golgi apparatus,” “symplast,” and “protein–DNA complex,” were present (Supplementary Fig. S6C). In the EV samples prepared without EDTA extraction, we observed 72 proteins that were additional to those present in the EDTA-extracted EV samples, and, thus, potentially bound to pectins (Supplementary Table S3A). The GO categories for biological function, such as “response to stress,” “reactive oxygen species metabolic process,” “carbohydrate metabolic process,” and “cell–cell signaling,” were prevalent for these 72 proteins (Supplementary Fig. S7A), and GO categories for molecular function included terms “antioxidant activity,” “oxidoreductase activity,” and “hydrolase activity” (Supplementary Fig. S7B). GO categories for cellular component included “extracellular region, apoplast, external encapsulating structure,” “non-membrane-bounded organelle,” and “DNA-packaging complex” (Supplementary Fig. S7C). Pectins have been earlier detected also in EV preparations of olive (*Olea europaea*) isolated from the germination medium of pollen (Prado et al. 2014). Instead of being located in the EV lumen like discussed by Prado et al., it is possible that pectins that were sloughed off from the cell wall into the germination medium were co-pelleted with EVs during ultracentrifugation. Hence, for the proteomic analyses of EVs isolated from tissue/pollen culture media or from actively growing tissues, EDTA extraction needs to be done to remove any co-pelleted pectin to decrease the presence of pectin-bound protein contaminants.

To assess the presence of potential false-positive soluble proteins from the culture medium that were possibly trapped in the EV pellets, 11.8 µL (corresponding to ∼15% of the volume of the pellets) of the unconcentrated EV-depleted culture medium samples (i.e. culture medium where EVs had been pelleted away) was prepared for the proteomic analysis. In these samples, 10 proteins were detected (Supplementary Table S3C). Many of these were pathogenesis-related proteins (van Loon et al. 2006) annotated as an antimicrobial protein, a chitinase, lipid-transfer proteins, and peroxidases. Eight proteins that were detected in the unconcentrated EV-depleted culture medium were also detected in the EV samples (Supplementary Fig. S8A; Supplementary Table S3B). However, 549 proteins were detected solely in the EV samples, an observation which suggests that the potential contamination from the soluble culture medium proteins was minor (∼1.4%).

Proteomic analysis of the concentrated EV-depleted culture medium of both lignin-forming and non-lignin-forming conditions was performed to compare the soluble culture medium proteins with those in the EV samples (Supplementary Fig. S8B; Supplementary Table S3D). Among 218 proteins detected in the concentrated medium, the GO categories for biological function included various “catabolic” or “metabolic processes” (Supplementary Fig. S9A). Categories for “response to stress or stimulus,” “regulation of function or process,” and “cell wall organization or biogenesis” were also present. For molecular function, GO terms, such as “hydrolase activity,” “tetrapyrrole binding,” “antioxidant activity,” and “oxidoreductase activity,” were enriched (Supplementary Fig. S9B). The GO-term enrichment for cellular component gave 8 categories, with 5 corresponding to “apoplast” (Supplementary Fig. S9C).

Fifty-five proteins (∼25%) were found solely in the concentrated culture medium after extensive (100-fold) concentration. However, 163 proteins were found in both the concentrated culture medium and in the EV samples (Supplementary Fig. S8B). For these common proteins, the GO categories for biological function contained various “metabolic processes,” for example, “reactive oxygen species metabolic process” (Supplementary Fig. S10A). Categories for “response to stress or stimuli” and “cell wall organization or biogenesis” were also present. The GO-term enrichment for molecular function included various hydrolases, transferases, and oxidoreductases (Supplementary Fig. S10B), while that for cellular component gave 6 terms, 5 of them corresponding to “apoplast” (Supplementary Fig. S10C). These observations suggest that EVs can indeed transport proteins into the apoplast and complement secretion conducted with the conventional protein secretion pathway. When the next version (ver. 2) of the Norway spruce genome will be published with improved gene models, it will be possible to evaluate the presence of signal peptides in the sequences of EV proteins in order to investigate the proportion of proteins that lack the signal peptide. After release from EVs by a yet unknown mechanism, proteins either remain soluble in the apoplastic fluid or bind to cell wall polymers, such as pectin.

**Figure 6.**
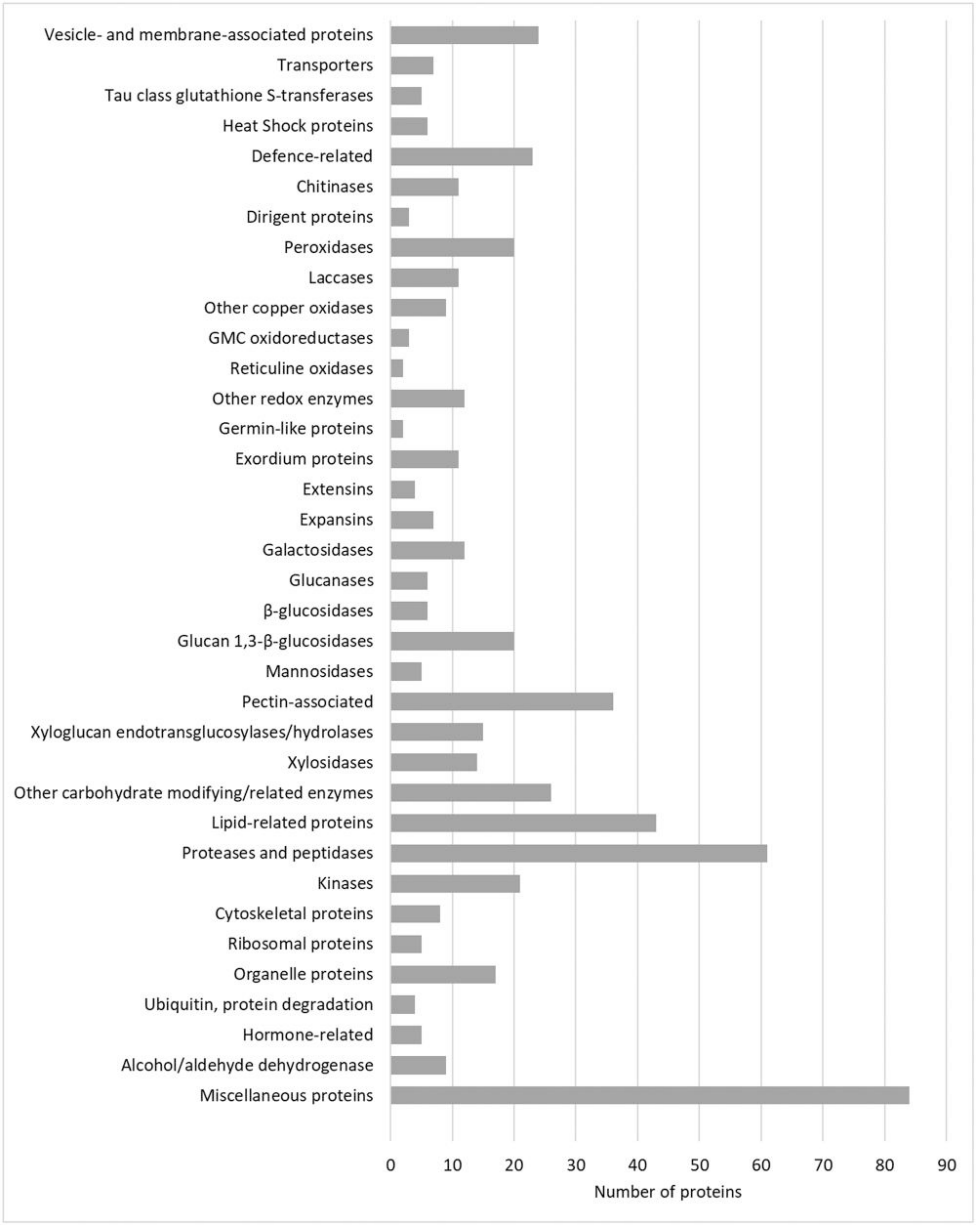
Proteomic analysis of the extracellular vesicle (EV) samples of Norway spruce. Distribution of proteins (*n* = 557) detected in the EV samples in groups with annotated functions.

**Figure 7.**
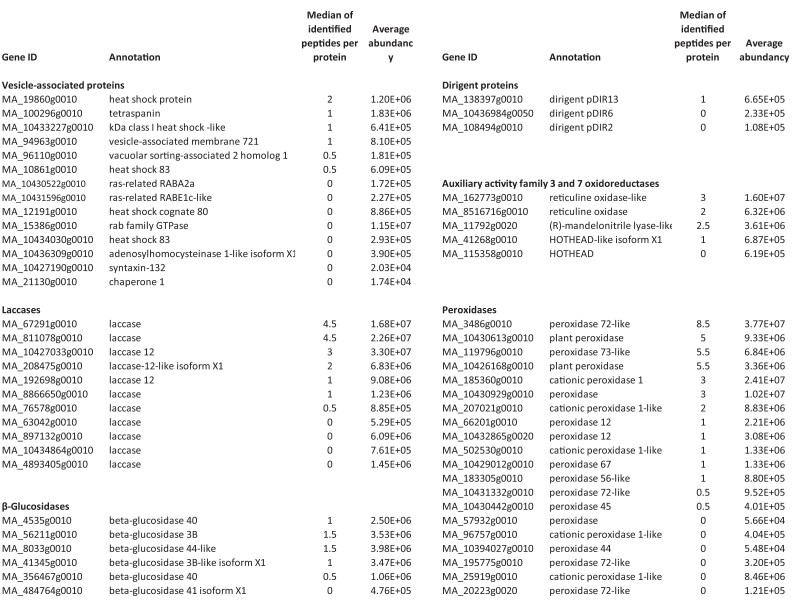
Proteins detected in extracellular vesicle (EV)-enriched samples of Norway spruce, and their annotated functions. The median and the average abundancy of unique peptides per protein across all samples are also shown. Proteins shown are listed based on their reported presence in EVs or their putative role in lignin biosynthesis. A wider list of proteins detected is given in Supplementary Table S3.

The spruce EV samples contained various cell wall proteins, together with some proteases, receptor kinases, and pathogenesis-related proteins (e.g. chitinases and thaumatins) (Supplementary Table S3B, Fig. 6). Thus, the cargo was broadly similar to that identified in EVs isolated from the extracellular fluids of uninfected sunflower seedlings (Regente et al. 2017). In addition, 6 heat shock proteins and an *S*-adenosyl-homocysteinase were detected (Fig. 7); these are commonly found in EVs of plants and mammals (Pinedo et al. 2021). A homolog (MA_100296g0010) of one of the commonly seen proteins associated with EVs, tetraspanin 8 (TET8; e.g. Huang et al. 2021), was identified in our proteomic dataset (Fig. 7; Supplementary Table S3B). Of the proteins that are known to be important for vesicle trafficking and membrane fusion (Zhang et al. 2011; Ichikawa et al. 2014; Ito and Uemura 2022), rat sarcoma virus (Ras)-associated binding (RAB) GTPase homologs, a syntaxin, and a vesicle-associated membrane protein (VAMP) were observed in Norway spruce EVs (Fig. 7; Supplementary Table S3B). In *Arabidopsis* leaves, a syntaxin protein121 (AtSYP121/PENETRATION1; PEN1) occurs in a specific subclass of EVs that differs from those that contain TET8 (Rutter and Innes 2017; Huang et al. 2021). In addition, a protein (MA_96110g0010) putatively associated with the ESCRT III complex assembly was found in the proteomic data of spruce EVs. This protein shares the highest similarity with an *Arabidopsis* sucrose non-fermenting 7 (SNF7)-family protein (AT2G06530) interacting with the apoptosis-linked gene 2 (ALG-2)-interacting protein X (ALIX, also known as programmed cell death 6-interacting protein)—a protein functioning in the trafficking of cargo to the vesicles (Cardona-Lopez et al. 2015).

The proteomic profiles of the spruce EV samples show several proteins putatively related to the lignin formation and the modification of cell wall components (Fig. 6; Supplementary Table S3B). Some of the laccases (MA_67291g0010, MA_192698g0010, MA_208475g0010, MA_4893405g0010, MA_8866650g0010, MA_10427033g0010) and peroxidases (MA_57932g0010, MA_66201g0010, MA_185360g0010, MA_502530g0010, MA_10430929g0010, MA_10431332g0010, MA_10432865g0020) present in the EV samples (Supplementary Table S3B) co-express with the core monolignol biosynthesis genes (Jokipii-Lukkari et al. 2018) in lignin-forming cultured cells or during wood formation in trees (Väisänen et al. 2020). This observation also provides additional candidates for monolignol-oxidizing oxidoreductases since these are not the same as previously identified by Jokipii-Lukkari et al. (2018) based on co-expression with the monolignol biosynthesis genes in developing xylem of Norway spruce. A peroxidase MA_3486 was detected both in the EVs and in the concentrated culture medium. Interestingly, this is the peroxidase PaPx16/17 described previously in Koutaniemi et al. (2007) and Warinowski et al. (2016), which is bound to extracellular lignin. Oxidative enzymes present in this protein fraction were shown to produce a dehydrogenation polymer (DHP) that is more similar to native wood lignin in terms of the subunit linkage structure compared to any other DHP (Warinowski et al. 2016). The observation that proteins in the EV samples, putatively pectin-bound proteins, and EV-depleted culture medium proteins contain different isoenzymes of peroxidases and laccases (Supplementary Table S3) indicates that proteins present in the culture medium of Norway spruce can be further separated into additional groups with unique isoenzyme patterns. It is possible that, in a similar way to *Arabidopsis* (Chou et al. 2018; Hoffmann et al. 2020; Blaschek et al. 2023), separate isoenzymes of oxidative enzymes can localize to different cell wall layers of Norway spruce and could potentially have different specificities and nonoverlapping roles in the oxidation of phenolic compounds.

Three proteins (MA_108494g0010, MA_138397g0010, and MA_10436984g0050) were annotated as dirigent proteins (Fig. 7; Supplementary Table S3B). Genes encoding 2 of these proteins are highly induced during lignin-forming conditions (Laitinen et al. 2017) and co-express with the core monolignol biosynthesis genes (Väisänen et al. 2020). Although there is no biochemical evidence for the participation of dirigent proteins in lignin biosynthesis, it has previously been hypothesized that arrays of dirigent sites could function as lignification initiation sites by guiding the initial assembly of monolignols (Guan et al. 1997; Gang et al. 1999). Furthermore, specific dirigent proteins have been reported to be crucial for lignin formation in the Casparian strip in the roots of *Arabidopsis* and maize (*Zea mays*) (Hosmani et al. 2013; Wang et al. 2022; Gao et al. 2023). In lignin polymerization, the combinatorial chemical coupling of monolignols is favored over the hypothesis involving arrays of dirigent proteins (Ralph et al. 2004). Nonetheless, certain dirigent proteins have been shown to guide the stereoselective coupling of 2 phenolic radicals generated by the action of an oxidative enzyme to form certain enantiomers of lignans (Davin et al. 1997; Yonekura-Sakakibara et al. 2021). For example, pinoresinol synthesis yields (+)-pinoresinol in the presence of a dirigent protein and a racemic mixture of (+)- and (−)-pinoresinol (a mirror image) when the dirigent protein is excluded (Davin et al. 1997). The occurrence of dirigent proteins in the EV samples of Norway spruce together with monolignols and oxidative enzymes highlights the need for further studies on the role of dirigent proteins in lignin biosynthesis. Alternatively, dirigent proteins could be involved in lignan biosynthesis in spruce EVs for defense purposes. Currently, we have a study going on to investigate the role of dirigent proteins in Norway spruce.

Transporter proteins of various classes, such as ABC (MA_8849g0010, MA_10432169g0010), and major facilitator superfamily (MFS, MA_130810g0010) were detected in the proteomes of the EV samples (Supplementary Table S3B). These transporter superfamilies have been previously studied or discussed in relation to the monolignol transport (Miao and Liu 2010; Alejandro et al. 2012; Väisänen et al. 2020), and detecting these proteins in the EV samples raises the question of whether or not they could be used in vesicle loading. One ABC transporter detected here belongs to the ABCB subclass, of which some members in *Arabidopsis* co-express with phenylpropanoid biosynthetic genes (Ehlting et al. 2005), although none have been identified as being responsible for the monolignol transport (Kaneda et al. 2011). Previously, a secondary active transport of coniferin and *p*-coumaryl alcohol glucoside was detected in biochemical assays of Norway spruce, pointing to the possible involvement of MFS and/or multidrug and toxic compound extrusion (MATE) transporters (Väisänen et al. 2020). Hypothetically, vesicles could be loaded via the secondary active transport with these monolignol glucosides (or with other monolignol hexosides). If so, this would necessitate a role for β-glucosidases to cleave the glucose moieties and allow for monolignol oxidation and radical coupling to proceed. As mentioned, 6 β-glucosidases were detected in the EV samples (Fig. 7; Supplementary Table S3B).

Five proteins were annotated as “tau class glutathione *S*-transferases” (Supplementary Table S3B). Glutathione *S*-transferases (GST) play a role in detoxification by conjugating xenobiotics to glutathione, which is then followed by the transfer of the conjugates into the apoplast or sequestration into vacuoles (Coleman et al. 1997). In a cell culture of grapevine (*Vitis vinifera*), a tau-class GST was shown to be involved in the transport of *trans*-resveratrol, a stilbene, into the culture medium; however, no glutathione conjugate of *trans*-resveratrol was detected (Martínez-Márquez et al. 2017). The protein was hypothesized to be bound to the plasma membrane via protein–protein interactions and could be part of a still-unknown molecular machinery for the *trans*-resveratrol transport into the apoplast (Martínez-Márquez et al. 2017). Further studies are needed to investigate whether either of the tau-class GST detected in the present study functions in monolignol transport in a similar way to that for the structurally related stilbene and whether they are localized specifically in EVs or also in the plasma membrane.

Reticuline oxidases (berberine bridge enzyme (BBE)-like proteins with oxidoreductase activities; MA_162773g0010, MA_8516716g0010) were detected in the EV samples (Fig. 7; Supplementary Table S3B). Some BBE-like proteins use mono- and oligosaccharides as substrates and generate H_2_O_2_ (Carter and Thornburg 2004; Benedetti et al. 2018; Locci et al. 2019). Others use monolignols as substrates. For example, *Arabidopsis* AtBBE-like 13 and AtBBE-like 15 oxidize monolignol alcohols and coniferin into corresponding aldehydes (Daniel et al. 2015). This kind of activity could conceivably contribute to the formation of coniferaldehyde detected in the EV samples (Fig. 3, A and B; Supplementary Fig. S3; Supplementary Table S1A). In addition, 3 members of a glucose–methanol–choline (GMC) oxidoreductase family were detected in the EV samples (annotated as (R)-mandelonitrile lyase-like (MA_11792g0020) and HOTHEAD-like (MA_115358g0010, MA_41268g0010); Fig. 7; Supplementary Table S3B). Out of these, MA_11792g0020 was shown to be induced in lignin-forming conditions (Laitinen et al. 2017). Some members of this family function as oxidases utilizing carbohydrates, including glucose as substrates. In this case, the products of the enzymatic reaction are oxidized sugar and H_2_O_2_ (Sützl et al. 2018). By contrast, other members function as dehydrogenases and oxidize monolignols into their corresponding monolignol aldehydes (Sützl et al. 2018; Roy et al. 2022). Members of both the abovementioned enzyme families are possible sources for H_2_O_2_ needed for the peroxidase action. Mammalian EVs have also been shown to transport functional ROS-generating machinery (NADPH oxidases) to the sites needed (Hervera et al. 2018).

**Figure 8.**
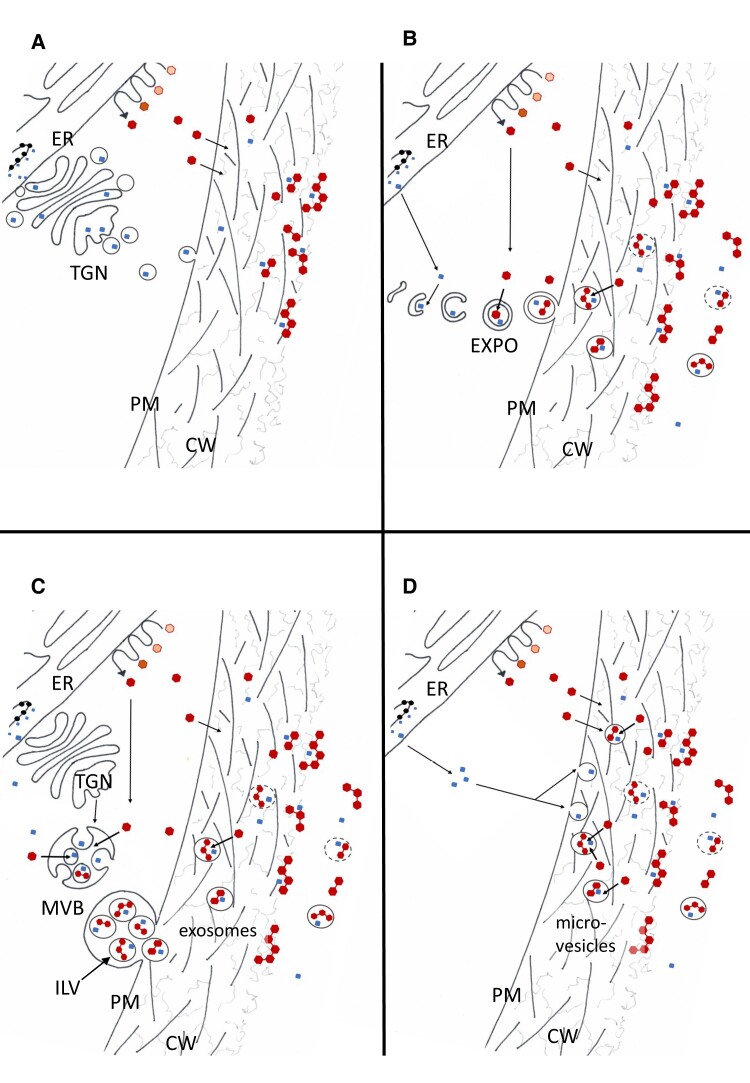
Hypothetical pathways for the export of oxidative enzymes (squares) and lignin mono-, di-, and oligomers (hexagons with varying linkages) to the apoplastic space in tissue-cultured cells of Norway spruce during lignin formation. **A)** Conventional protein secretion exports oxidative enzymes that are synthesized in the endoplasmic reticulum (ER), modified in the Golgi apparatus, sorted in the *trans*-Golgi network (TGN), and transported in Golgi vesicles to the plasma membrane (PM), where the vesicles fuse with the PM to release oxidative enzymes. In addition, monolignols are synthesized via the phenylpropanoid and monolignol biosynthesis pathways (waved arrow indicates the pathways and intermediates are represented with hexagons), with some enzymes associated with the ER and transported through the PM via diffusion and/or a transporter-mediated transport. Monolignol oxidation and polymerization occur in the apoplast (cell wall, and the culture medium in case of cultured cells). **B)** Double membrane-containing exocyst-positive organelles (EXPOs) form in the cytoplasm, thereby sealing the oxidative enzymes inside. Monolignols diffuse along the concentration gradient or are actively transported to the vesicles where they are radicalized by the oxidative enzymes and begin to polymerize. The outer membranes of EXPOs fuse with the PM, releasing the inner vesicle (now called an extracellular vesicle, EV) into the apoplast. **C)** Multivesicular bodies (MVBs) originate from the TGN and contain vesicles (intraluminal vesicles, ILVs) that form by the inward budding of the MVB membrane. Oxidative enzymes get trapped inside the vesicles. Monolignols could follow the oxidative enzymes into the vesicles in a similar way as described above in **B)**. MVB fuses with the PM releasing ILVs into the apoplast. EVs secreted in this way are called exosomes. In **B)** and **C)**, the monolignol accumulation in the EVs could continue in the apoplast, with the monolignols secreted directly through the PM. **D)** Microvesicle-type EVs are produced by blebbing of the PM into the apoplast and seal proteins inside. These EVs accumulate monolignols only in the apoplast; thus, the original monolignol transport through PM would be based on diffusion and/or transporter-mediated transport. In **B)** to **D)**, the EVs finally release their contents to the apoplast by a yet unknown unloading mechanism.

Polysaccharide-modifying enzymes (Kumar et al. 2019) were present in the EV samples (Fig. 6; Supplementary Table S3, A and B). Various glycosyl hydrolases, transglucosylases/hydrolases, pectin-modifying enzymes, and expansins were detected. Several proteins were also annotated as homologs of SKU5 or exordium; these enzymes function during cell growth (Sedbrook et al. 2002; Schröder et al. 2009). Cell wall-modifying enzymes, together with nonenzymatic expansins, are important for the controlled loosening of the cell wall to allow cell extension (Cosgrove 2005, 2022). As these proteins were detected in the EV samples, we propose that similar to that observed in germinating sunflower seedlings (Regente et al. 2012, 2017; de la Canal and Pinedo 2018), not all members of these cell wall-modifying enzyme families are transported to the apoplast via the conventional protein secretion pathway (CPS; Fig. 8A). Similarly, in *Arabidopsis*, 1 xyloglucan transglucosylase/hydrolase isoenzyme was reported to be transported to the apoplast via unconventional protein secretion mediated by EXPOs; the other isoenzymes studied were transported via the CPS pathway (De Caroli et al 2021). Also, a protein annotated as *S*-adenosylmethionine synthase 2 (MA_197296g0010) was detected in the EV samples of Norway spruce (Supplementary Table S3B). In *Arabidopsis*, *S*-adenosyl methionine synthase 2, which contains no signal peptide, was shown to sequester with EXPOs and proposed to be transported to the apoplast (Wang et al. 2010). Interestingly, this protein was also detected in the EVs isolated from the germination medium of the pollen of olive (Prado et al. 2014). Our observations corroborate the findings of Regente et al. (2017) and those of de la Canal and Pinedo (2018) that EVs may have an important role in cell wall remodeling.

Our experimental setup used the culture medium of tissue-cultured cells of Norway spruce to isolate vesicles that are natively secreted by cells. This system does not remove common cellular proteins completely since the differential ultracentrifugation-based isolation procedure is not specific for EVs. Accordingly, some co-isolating soluble proteins (e.g. histones, actin, and ribosomal proteins) were also found in the proteomic data to varying degrees (Supplementary Tables S3, A and B). However, following the critical assessment of our data, we can conclude that only a minority of the proteins detected had originated in organelles, such as the nucleus (e.g. histones), chloroplast, or mitochondrion.

### Proteins involved in SA signaling were detected in the EV samples

In addition to SA (Fig. 5; Supplementary Fig. S5; Supplementary Table S1), the EV samples contained 2 homologs of “defective in induced resistance1” (DIR1) proteins (Supplementary Table S3B). One of these was also detected in the unconcentrated culture medium (Supplementary Table S3C), and both were detected in the concentrated culture medium (Supplementary Table S3D). DIR1 is a lipid-transfer protein that is involved in SAR signaling in *Arabidopsis* (Maldonado et al. 2002). In addition to *Arabidopsis*, several other plants have putative orthologs of DIR1, and at least in cucumber (*Cucumis sativus*), the role of DIR1 in SAR signaling is conserved (Isaacs et al. 2016). As such, it seems that EVs may play an important role in SA signaling by transporting SA and proteins involved in SAR.

### EVs as a mechanism for monolignol transport and platforms for monolignol oligomerization

In our experimental system, we detected coniferyl alcohol and coniferaldehyde in the EV samples (Fig. 3, A and B; Supplementary Fig. S3; Supplementary Table S1), but not in the culture media. This might be partly due to the ongoing polymerization of monolignols in the medium. Based on computational modeling, mono-, di-, and oligolignols can diffuse through membranes (Vermaas et al. 2019). Hence, the di- and oligolignols detected in the EV samples may have diffused into the EVs from the culture medium (Fig. 8). However, the observation that many phenolic acids (e.g. cinnamic, ferulic, and vanillic acid) (Supplementary Table S1; Supplementary Fig. S3) that are not able to diffuse across membranes due to their charge (Vermaas et al. 2019) were present only in EVs and not in the medium suggest that at least some loading of the EVs occurred in the cytosol rather than in the apoplast. In the cytosol, the EV loading could be accomplished partially via diffusion (non-charged phenolics), which is perhaps driven by a polymerization-induced concentration gradient. The presence of oxidative enzymes in the EV lumen could create a sink that drives diffusion through the membrane as was observed in vitro with artificial liposomes (Perkins et al. 2022). It is also possible that loading occurs via an active mechanism, such as an ABC or an MFS transporter (Väisänen et al. 2020), located on any of the membranes associated with the EV formation. EVs could conceivably transport mono-, di-, and oligolignols to the middle lamella and cell corners, which are locations farthest away from the plasma membrane and where it is known that lignification initiates (Fig. 8; Terashima and Fukushima 1988; Donaldson 2001; Marjamaa et al. 2003). EVs could also be a way to transport lignin precursors from the monolignol-synthesizing neighboring cells, whose cell walls do not lignify, to the lignifying cell walls of adjacent cells (a good neighbor hypothesis, Pesquet et al. 2013; Smith et al. 2013). The biosynthesis of monolignols could be co-localized with the formation of EVs. Modifications, such as the ubiquitinylation of enzymes required, for example, in monolignol oxidation, could assimilate and target these toward EVs (Fig. 8; Smith et al. 2015; reviewed in Carnino et al. 2020). Norway spruce EVs, however, were detected to contain relatively low levels of metabolites as compared to the culture medium. This observation suggests that lignin precursors are transported mostly via passive diffusion and/or transporter-mediated transport and not via the EV-mediated transport. However, without knowing the lifetime of EVs, this remains hypothetical. The alternative hypothesis is that EVs do play a major role in lignin precursor transport, but that they quickly release their cargo (and disintegrate).

Since phenolic compounds can diffuse across membranes, in order to stay within EVs, the compounds must be conjugated with another moiety (e.g. glucose) to decrease diffusion through the membrane. Some glucose conjugates were shown to be enriched in EVs, similar to some rather uncommon 7-hydroxydihydro and 7,8-dihydroxydihydro derivates (Fig. 4; Supplementary Table S2). Alternatively, the compounds must be so highly oligomerized that their ability to diffuse through the membrane decreases (Vermaas et al. 2019). The methods now used in the metabolomic analysis did not allow us to detect oligolignols bigger than tetramers. Some diphenols, however, were shown to be enriched in EVs, suggesting that phenolic coupling is going on in EVs. The membrane could have a distinctive composition of lipids and proteins that decreases the propensity for diffusion. Some bacterial membranes, for example, have lipopolysaccharides that limit the diffusion of hydrophobic molecules through membranes (van den Berg 2010). The composition of the membrane lipids of plant EVs has not been extensively studied. The lipid composition of the membrane bilayer of the EVs isolated from *Arabidopsis* leaves was reportedly substantially different from that of other leaf membranes, with a remarkably elevated content of sphingolipids (∼46% and ∼0.5% in EV membranes and other cellular membranes, respectively; Liu et al 2020b). Negatively charged glycosylinositolphosphoceramides (GIPCs) with glucuronic acid and a hexose in the head group are the predominant species of sphingolipids (∼99% of all) in the membranes of *Arabidopsis* EVs. Interestingly, Cacas et al. (2016) observed that GIPCs are enriched also in the outer leaflet of the plasma membrane in tobacco leaves and BY-2 cells and locate especially in the detergent-insoluble microdomains. In contrast to van den Berg (2010), modeling suggests that even highly glycosylated lipopolysaccharides only mildly affect the diffusion rates of monolignols across the membranes (Vermaas et al. 2022). It should be remembered, however, that biological membranes are complex structures, and the physical interactions of their components in the presence of ions and ion gradients are difficult to predict. When phenolic compounds are added in biophysical experiments into simplified membrane systems together with Na^+^ or Ca^2+^, different effects are seen depending on the phenolic molecule and the ion species (Hossain et al. 2021, 2022). To our knowledge, the relationship between mono-, di-, or oligolignols with membranes in the presence of ions has not been studied, and it is not known whether ions affect these compounds and their possible interactions with lipids.

According to the modeling experiments of Vermaas et al. (2019), mono- and dilignols can enrich in the lipid bilayer. The presence of membranous vesicles in the apoplast could thus have an impact on lignification by serving as platforms to control polymerization reactions. The effect would come from the fact that many mono- and dilignols can easily diffuse through the membranes, and the oxidative enzymes in the EV lumen would create a sink. In this way, EVs would serve as the nucleation points for lignin polymerization. The observation that EVs contain coupling products of monolignols supports this hypothesis. After liberation to the apoplast, di- and oligolignols could continue further coupling and grow to lignin polymers. The lipid composition of the bilayer may have some effect on the diffusion of phenolic compounds (Boija and Johansson 2006; Vermaas et al. 2019), meaning that the lipidomic analysis of EVs represents an important area for future research. In addition to the membrane surface of EVs, there is a microenvironment inside the vesicles. Factors, such as pH (Grabber et al. 2003), monolignol species (Demont-Caulet et al. 2010), and the ratio of monolignols and the oxidative enzymes or end products (Méchin et al. 2007, Warinowski et al. 2016), can all affect lignin polymerization. These conditions could conceivably be different in the EV lumen as compared to the surrounding apoplast.

If EVs carry lignin precursors and SA into the apoplast as our study suggests, the mechanism that leads to the unloading of the vesicles is another important question to address. It remains to be seen whether there are certain proteins in the cell wall (e.g. in the pectin-rich middle lamellae and cell corners) that lead to opening of the EVs in an analogous way to the specific receptor-mediated membrane fusion of EVs to target cells in animals, such as syncytin-1 and others (reviewed by Prada and Meldolesi 2016). If specific opening sites existed for EVs, EVs could potentially provide lignin precursors a protective barrier such that they could be transported to places far away from the plasma membrane (middle lamella, cell corners), where lignification starts in muro. Wang et al. (2010) hypothesized that differences in the osmolarity in the symplast (higher osmolarity) and the apoplast (lower osmolarity) would lead to bursting of EXPOs and liberation of their contents into the apoplast. Not all proteins now detected to be present in the EV samples (Supplementary Table S3B) were present in the concentrated culture medium (Supplementary Table S3D), but part of the proteins carried by EVs is likely to be bound to the cell wall polymers. Unloading should also be coupled with the recycling of the now-empty EVs back to the protoplast. Recycling of the burst vesicles could, therefore, be seen as phagocytosis rather than specific pathway-mediated transport. All in all, further studies of the middle lamella microenvironment could unveil the proteins or factors required for the initiation of lignification.

### EVs—single or multiple types?

The study of plant EVs has only just begun. It has not yet been resolved whether or not plants have different types of EVs serving different functions. The morphological and size differences observed in Norway spruce EVs (Fig. 1, B and C) suggest that several types of EVs were present in the culture medium. The origin of EVs in plant cells is not as well-known as in animal cells, but hypothetically, EVs in cultured spruce cells could form and capture proteins via several mechanisms (Fig. 8). EXPOs form in the cytoplasm and could seal oxidative enzymes inside, whereas monolignols could either diffuse along the concentration gradient or be actively transported into these vesicles (Fig. 8B). Fusion of the outer membrane of EXPOs with the PM releases the inner vesicle into the apoplast. We did not, however, detect Exo70E2, which is a membrane protein present in EXPOs, in the proteomic analysis. Considering the challenges in the proteomic analyses of membrane proteins and shortages in many gene models of the Version 1.0 of the Norway spruce genome assembly (see discussion above), we think that this possibility is still worth considering. An additional reason may also be due to the low abundance of the EXPO pathway vesicles in our wild-type samples in contrast to the high accumulation of EVs in *exo70a1*-deficient *Arabidopsis* mutants, as described in detail by De Bellis et al. (2022). The presence of exosomes (Fig. 8C) and/or microvesicles (Fig. 8D) in the EV preparation is supported by the detection of a putative tetraspanin in the proteomic data (Fig. 7; Supplementary Table S3B). Tetraspanin proteins are present in the outer membranes of both exosomes and microvesicles (van Niel et al. 2018). Microvesicles are produced by blebbing of the plasma membrane, whereas exosomes originate from the fusion of multivesicular bodies (MVBs) with the PM (Rutter and Innes 2017; Huang et al. 2021). MVBs originate from the *trans*-Golgi network (TGN) and contain intraluminal vesicles (ILVs) that form by inward budding of the MVB membrane. Oxidative enzymes could get trapped inside the vesicles at this budding stage. Monolignols could be loaded into the vesicles either by diffusion or by active transport. When a MVB fuses with the PM, ILVs are released into the apoplast as exosomes. One possibility hypothesized by De Bellis et al. (2022) was that EVs could form from the ER membranes by invagination into the ER lumen, and this structure would eventually fuse with the PM. Some enzymes involved in monolignol biosynthesis are known to be attached to ER; hence, vesicles’ and monolignols’ synthesis places would co-occur facilitating loading of lignin precursors into the vesicles. The interaction between ER and PM has been shown, and many of the proteins involved have been identified (Li et al. 2021). These contact sites together with large vacuoles present in plant cells pushing the cytoplasm with its organelles to the proximity to PM makes this possibility worth investigating.

The EV samples of Norway spruce were collected by ultracentrifugation, and this strategy could not separate distinct EV types that were shown to be present in the apoplast (Fig. 1, B and C). Only apoptotic bodies were likely to be pelleted in the first centrifugation step (10,000× *g* for 30 min). Our trials for the further separation of the EVs by size or density were not successful probably because of the tendency for spruce EVs to agglomerate into larger aggregates. A density-based separation can further be complicated by the varying densities of EVs in different plant species (1.03 to 1.29 g/mL; Prado et al. 2014; Rutter and Innes 2017). Without any previous data on Norway spruce EVs, we were not able to recover sufficient amounts of vesicles to continue with those experiments. Thus, it is possible that some metabolites and proteins now detected in the EV samples are actually derived from separate EV types. Further studies will show, for example, whether or not distinct EVs carry enzymes related to lignin formation and those related to modeling of cell wall polysaccharides. This type of categorization could lead to discoveries of specific target destinations for each EV subtype or different functionalities related to the EV cargo. Now that we have accumulated proteomic and metabolomic data from the cell cultures, the logical next step is to study EVs present in the developing xylem of trees. The data obtained will help in designing strategies, for example, for affinity purification of EVs, from the more challenging native situation in tree trunks where developing xylem is located under other tissues (i.e. bark, phloem, and cambium). One aspect that needs to be considered in the future studies is the fact that developing tracheids undergo cell death and autolysis during development (Bollhöner et al. 2012), making the native situation even more challenging.

## Materials and methods

### Plant material

Norway spruce (*Picea abies* L. Karst.) tissue-cultured cells (line A3/85) were maintained on a solid nutrient medium with a 16/8 h light/dark cycle at 25 °C, as described in Kärkönen et al. (2002). The experimental setup was similar to that in Laitinen et al. (2017), but with some modifications. No organic nitrogen was added to either solid or liquid medium, except for glycine in the vitamin solution. Clumps of callus cells were transferred into liquid medium 5′ ca. 2.5 wk after sub-culturing (Simola and Santanen 1990) (ca. 2 g cells/100 mL medium in a 500-mL flask), and fresh, filter-sterilized KI solution (final concentration 5 mm) or an equivalent volume of water was added to the medium (Fig. 1A; Laitinen et al. 2017). Cell suspensions were grown on an orbital shaker (100 rpm) at +22 °C in a 16 h light/8 h dark cycle (20 to 50 µmol m^−2^ s^−1^, Osram warm white). The culture medium of individual flasks (*n* = 4) for both treatments was sampled at Days 7, 10, and 14 after the start of the liquid cultures. The cells were left to sediment to the bottom of the flasks. The medium was aliquoted to into 50 mL tubes, and the remaining cells were left to sediment again after which the medium was pipetted into new tubes and stored at −20 °C or −80 °C until further analysis. The culturing experiment was conducted 4 times with 4 replicate flasks for each timepoint and each treatment.

### Isolation of the EV samples

The EV samples were isolated from the culture medium using differential ultracentrifugation based on a protocol modified from that of Rutter and Innes (2017) (Supplementary Fig. S1). Frozen culture medium (ca. 45 mL) was quickly thawed in a 50 °C shaking water bath. Immediately after thawing, the medium was centrifuged at 10,000 × *g* (Sorvall Lynx 6000, Fiberlite F14-14×50cy) for 30 min at 4 °C to pellet the extracellular lignin (if present) and any cellular debris. The resulting clear supernatant was decanted and centrifuged at 100,000 × *g* (Beckman Coulter L-80, Type 50.2 Ti) for 90 min at 10 °C to pellet EVs. The resulting pellets were then resuspended in 250 µL of phosphate buffered saline, pH 7.4 (PBS), aliquoted prior to freezing with liquid nitrogen, and stored at −80 °C. The EV-enriched pellets prepared for the proteomic analysis were resuspended in 20 mm EDTA in PBS, and the suspensions were diluted to a final volume of 18 mL with 20 mm EDTA in PBS and centrifuged again as above to remove any co-precipitated pectin.

### Validation of EVs

According to the online guide of EV-Track (EV-Track.org) and the guidelines of ISEV (Lötvall et al. 2014; Théry et al. 2018), the EV samples were characterized by the nanoparticle tracking analysis (NTA) using Malvern Panalytical Nanosight LM14C and by transmission electron microscopy (JEOL JEM-1400) according to Puhka et al. (2017) at the HiPREP CORE facility at the University of Helsinki. No commercially available antibodies were available that are specific to spruce EV proteins; thus, Western blotting was not conducted.

The samples were subjected to additional purification with size exclusion chromatography and sucrose gradient centrifugation. However, the resulting fractions had inadequate yields likely due to the aggregation of EVs. Hence, EV samples without further purification were used in subsequent analyses.

### Antibody analysis of EVs

To investigate the possibility that some pectin that was sloughed off from the cell wall into the culture medium and co-pelleted in ultracentrifugation with the EVs, a dot blot analysis was conducted using an LM19 antibody (Kerafast) that recognizes weakly methylesterified homogalacturonan (Verhertbruggen et al. 2009). EV pellets with or without EDTA extraction were suspended in the equal volumes of PBS, and 15 µL of each was pipetted as a dot onto the nitrocellulose membrane (Amersham Hybond-ECL) in 3 dilutions. As a control, citrus pectin (Sigma Aldrich, P9135) was used. The antibody treatment was done in a custom manner using LM19 as the primary antibody (1:10 in TTBS supplemented with 2.5% (w/v) milk powder) and Alexa Fluor 647 goat anti-rat IgM (invitrogen) as a secondary antibody (1:1,000 in TTBS supplemented with 2.5% (w/v) milk powder). The signal was detected with the ChemiDoc MP Imaging System (Bio-Rad).

### Sample preparation for metabolomics

The EV pellets from ultracentrifugation were washed with 18 mL PBS and repelleted by ultracentrifugation. The pellets were then aspirated dry and extracted with 500 µL of 90% (v/v) methanol in Milli-Q water. The samples were incubated on a rocking platform for 30 min at room temperature in the dark, after which the visible debris was pelleted by centrifugation (13,000 × *g*, 20 min). The resulting supernatant was transferred into a new tube and dried overnight in a vacuum centrifuge. In parallel, 1 mL of the supernatant from each ultracentrifugation preparation of EVs was pelleted and dried to be used as a comparison sample (EV-depleted culture medium). After drying overnight in a vacuum centrifuge, the tubes were filled with nitrogen gas and stored at −80 °C.

For the UPLC–MS analyses, the dry medium samples were dissolved in 100 µL milli-Q water and loaded on an Oasis HLB solid phase extraction (SPE) cartridge (Waters Corporation, Milford, Massachusetts). The compounds were eluted from the SPE column with 1,250 µL of methanol. The eluent was dried and reconstituted in 100 µL Milli-Q water. The EV pellets were solubilized in 100 µL of cyclohexane and 100 µL of Milli-Q water. The aqueous phases were subsequently filtered on a 0.2 µm filter plate and analyzed by UPLC–MS (see below). For the GC–MS analyses, the dry medium samples were dissolved in 1,000 µL of Milli-Q water. Subsequently, 100µL was further processed via SPE in the same manner as the medium samples described above (i.e. elution with 1,250 µL of methanol, after which the eluent was dried). The dried residues obtained from the metabolite extraction of the EV-depleted media and of the EV pellets were then trimethylsilylated using 100 μL of a derivatization mixture (N-methyl-N-(trimethylsilyl)trifluoroacetamide:pyridine in a 5:1 ratio), and then analyzed through GC–MS (see below).

### UPLC–MS analyses

The samples were subjected to ultra-performance liquid chromatography–high-resolution mass spectrometry (UPLC–HRMS) at VIB Metabolomics Core Ghent (VIB-MCG). Ten µL was injected on a Waters Acquity UHPLC device connected to a Vion HDMS Q-TOF mass spectrometer (Waters, Manchester, UK). Chromatographic separation was carried out on an Acquity UPLC BEH C18 (150 × 2.1 mm, 1.7 μm) column (Waters, USA), with the column temperature maintained at 40 °C. A gradient of 2 eluents was used for separation as follows: eluent A (99:1:0.1 water:acetonitrile:formic acid, pH 3) and eluent B (99:1:0.1 acetonitrile:water:formic acid, pH 3): 99% A for 0 min decreased to 50% A over 30 min, decreased to 30% from 30 to 35 min, and finally decreased to 0% from 35 to 37 min. The flow rate was set to 0.35 mL min^−1^. Electrospray ionization (ESI) was applied, with the LockSpray ion source operating in a positive ionization mode under the following conditions: capillary voltage, 3 kV; reference capillary voltage, 3 kV; source temperature, 120 °C; desolvation gas temperature, 550 °C; desolvation gas flow, 800 L h^−1^; and cone gas flow, 50 L h^−1^. The collision energy for the full MS scan was set at 6 eV for low-energy settings. For high-energy settings (HDMSe), it was ramped from 20 to 70 eV. The mass range was set from 50 to 1,500 Da, and the scan time was 0.1 s. Nitrogen (greater than 99.5%) was employed as desolvation and cone gas. Leucine–enkephalin (100 pg μL^−1^ solubilized in water:acetonitrile 1:1 [v/v], with 0.1% (v/v) formic acid) was used for the lock mass calibration, with scanning every 2 min at a scan time of 0.1 s. The profile data were recorded using Unifi Workstation v2.0 (Waters). Data processing was performed with Progenesis QI v2.4 (Waters). The Pearson correlation coefficients between the peak intensities of 34 compounds detected in the EV samples and those in the corresponding medium samples and the relative intensities of 34 compounds in EV samples as compared to those in the corresponding medium samples were calculated in Microsoft Excel.

### GC–MS analyses

GC–MS analyses were performed on an Agilent 7250 QTOF-MS equipped with an Agilent 7890B GC system. One microliter of the derivatized sample was injected into the injection port in splitless mode, with the injector set to 280 °C. All biological samples were analyzed at random, and pooled samples were included for reproducibility purposes. Separation was achieved using a VF-5 ms column (40 m × 0.25 mm, 0.25 μm; Varian CP9013; Agilent), with helium as the carrier gas at a constant flow rate of 1.2 mL min^−1^. The oven was held at 80 °C for 1 min, ramped to 320 °C at a rate of 5 °C min^−1^, and then held at 320 °C for 5 min. The MS transfer line, MS ion source, and quadrupole were held at 280 °C, 230 °C, and 150 °C, respectively. The MS detector was operated in an electron ionization (EI) mode at 70 eV. Full EI–MS spectra were recorded by scanning the *m*/*z* range from 50 to 800 with a solvent delay of 7.8 min. The resulting GC–MS chromatograms were processed by Analyzer Pro XD software (spectral works). The following parameters were applied for peak detection and deconvolution: area threshold: 500; temporal resolution: minimum; scan window: 4; signal to noise: 3; smoothing: 15; and peak width: 0.05 min. The metabolites corresponding to the deconvoluted spectra were matched against the National Institute of Standards and Technology (NIST) ‘20 Mass Spectral Library to allow a tentative identification. The following parameters were applied for spectral matching: forward threshold: 650; reverse threshold: 650; confidence threshold: 60%; and confidence ratio: 70:30 (forward:reverse).

### Sample preparation for proteomic analyses

The EV samples treated with or without EDTA extraction from 2 and 3 biological replicates, respectively, and from 3 time points were used for the proteomic analysis. In addition, 5.0 mL of the EV-depleted culture media from 2 treatments (10 d, KI-10 d) with 3 biological replicates were concentrated with centrifugal filters (Pall Co, USA, cutoff 3 kDa) for the proteomic analysis. Accordingly, 30µg of the total protein of each sample from the EV samples based on a protein assay (Bio-Rad, USA) were used for the sample preparation. To assess the potential of the presence of the culture medium proteins trapped into the EV pellets leading to false-positive identifications, we estimated that the volumes of the EV pellets after EDTA extraction were ∼78 µL (diameter and height 10 and 1 mm, respectively). Estimating that a maximum of 15% of the medium could be trapped inside the pellets, 11.8 µL of the EV-depleted, unconcentrated culture media (i.e. culture medium where EVs had been pelleted away) were prepared for proteomics in 3 replicates. The samples were loaded on centrifugal filters (Pall Co, USA, cutoff 3 kDa) and centrifuged (13,000 × *g* at ca. +20 °C) until almost dry (Eppendorf 5804 R centrifuge). Urea buffer (UB, 400 µL) containing 8 M urea and 1% (w/v) sodium deoxycholate (SDC) in 10 mm Tris buffer, pH 8.0 (adapted from Zhou et al. 2006), was added on top of the EV samples on the centrifugal filters, incubated for 30 min at room temperature and centrifuged until minimal solution volume was left. To assure membrane solubilization, the proteins from the EDTA-extracted EV samples were solubilized with 5% (w/v) SDC in 100 mm ammonium bicarbonate at 70 °C for 30 min in microcentrifuge tubes (modified from Väisänen et al. 2020). The resulting solutions were diluted to a final concentration of 1% (w/v) SDC in UB, and then loaded on centrifugal filters (cutoff 3 kDa) and centrifuged (13,000 × *g* at ca. 20 °C) until minimal volumes remained (Eppendorf 5804 R centrifuge). UB (400 µL) was added on top of the EV samples on the centrifugal filters and centrifuged until almost dry. Denatured proteins were reduced with 10 mm DTT in UB for 30 min and centrifuged as before. Proteins were alkylated by adding 50 µL of 27 mm iodoacetamide in UB and incubating for 15 min in the dark. Proteins in filters were washed twice with 100 µL of UB. Filters were transferred to new collection tubes, and 1.2 µg of trypsin/lysC mixture (1:25 ratio, mass spec grade, Promega, USA) was added to the samples in 100 mm ammonium bicarbonate buffer (Promega USA) and incubated overnight at 37 °C. The following day, peptides were collected by centrifugation, after which the filters were washed twice with 50 µL of 0.5 m NaCl to elute the remaining peptides.

Formic acid (FA) was added to the peptide digests to lower the pH to below 3, and the peptides were purified using SepPak C18 columns (Waters Corp., USA). The samples were added to methanol-activated columns, washed twice with 0.1% FA (v/v), and eluted with 50% (v/v) acetonitrile. The resulting eluates were dried under vacuum in a vacuum centrifuge. Dried peptide digests were resuspended in 0.1% FA (v/v) solution, and the peptide concentration was estimated using a NanoDrop One device (Thermo Scientific). The remainder of the dried samples was dissolved in 30 µL 0.1% (v/v) trifluoroacetic acid and incubated in a ultrasonicating water bath for 10 min prior to the LC–MS/MS analysis.

### LC–MS/MS proteomic analysis and data processing

#### EV samples without EDTA extraction

The proteomic analysis was mainly performed as described in Väisänen et al. (2020). Accordingly, 3 to 5 µL of each sample was loaded on a BEH C18 analytical column (75 μm internal diameter × 250 mm, 1.7 μm particles; Waters Corp., MA, USA) and separated using a concave 68.5 min gradient of 1% to 40% solvent B (0.1% FA in acetonitrile) in solvent A (0.1% aqueous FA) at a flow rate of 300 nL min^−1^. The eluate was passed to a nano-electrospray ionization-equipped Synapt G2-Si HDMS mass spectrometer (Waters Corp., MA, USA) operating in a resolution mode. All data were in DDA option with dynamic range extension enabled using a scan time of 0.4 s and mass-corrected using Glu-fibrinopeptide B and Leu-enkephalin as the reference peptides.

The data from the DDA run were processed with PEAKS Studio 8.5 (Bioinformatics Solution Inc., Waterloo, Ontario, Canada). The resulting spectra were searched against the *Picea abies* 1.0 database (ConGenIE, http://congenie.org/; Nystedt et al. 2013; currently integrated to http://plantgenie.org; Sundell et al. 2015). The database search was done using a precursor and a fragment tolerance of 30 ppm and 0.15 Da, respectively. The identification of modifications was performed using the PEAKS PTM search option for multiple modifications.

#### EV samples with EDTA extraction and culture medium samples

Since the first LC–MS/MS instrument was no longer available, a different instrument was used in these subsequent experiments. Dried peptide digests were resuspended in 1% (v/v) TFA and sonicated in a water bath for 1 min before injection. Protein digests were analyzed using Nano-LC-Thermo Q Exactive HF. The peptides were separated using an UltiMate 3000 LC system (Thermo Scientific) equipped with a reverse-phase trapping column RP-2 C18 trap column (75 µm × 10 mm, Phenomenex, USA), followed by analytical separation on a Biozen C18 Nano Column (75 μm × 250 mm, 2.6 μm particles; Phenomenex, USA). The injected sample analytes were trapped at a flow rate of 5 µL min^−1^ in 100% solution A (0.1% formic acid). After trapping, the peptides were separated with a linear gradient comprising 33 min from 3% to 45% of solution B (0.1% formic acid/80% acetonitrile) and 4 min from 45% to 95% of solution B. Each sample injection was followed by a blank run to reduce the potential for sample carryover from previous runs.

LC–MS acquisition was done with the following mass spectrometer settings: resolution: 120,000 for MS scans and 15,000 for MS/MS scans. Full MS spectra were acquired from 350 to 1,400 *m*/*z*, and the 15 most abundant precursor ions were selected for fragmentation with 45 s dynamic exclusion time. Ions with 2+, 3+, and 4+ charges were selected for the MS/MS analysis. Secondary ions were isolated with a window of 1.2 *m*/*z*. The MS automatic gain control (AGC) target was set to 3 e^6^ counts, whereas the MS/MS AGC target was set to 2 e^5^. The NCE collision energy stepped was set to 28 kJ mol^−1^.

### Proteomic data and bioinformatics analysis

Following the LC–MS/MS acquisition, the raw files were qualitatively analyzed using Proteome Discoverer version 2.5 (Thermo Scientific, USA). The identification of proteins was performed using the *Picea abies* 1.0 protein database (ConGenIE, http://congenie.org/; Nystedt et al. 2013; currently integrated to http://plantgenie.org; Sundell et al. 2015), which was released in 2013 with 26,437 entries, using the built-in SEQUEST HT engine. The percolator node was used for the EV samples and concentrated culture medium samples to get more statistical data, and the fixed value validator node was utilized for very short protein lists of unconcentrated medium samples. The following parameters were used: 10 ppm and 0.02 Da were the error tolerance values for MS and MS/MS, respectively; trypsin was used as the digesting enzyme, and up to 2 missed cleavages were allowed; the carbamidomethylation of cysteine residues was set as a fixed modification, while the oxidation of methionine, deamidation of asparagine and glutamine, and phosphorylation of serine and threonine were set as variable modifications; the false discovery rate was set to be less than 0.01; and the minimum peptide length was 6 amino acids.

Protein identifications with at least 1 unique peptide from 2 or more samples or 2 or more unique peptides from a single sample were used for analysis in the protein lists of all samples (Supplementary Table S3). A Gene Ontology enrichment analysis was done on the plantgenie.org platform and exported for visualization as Treemaps to REVIGO (Supek et al. 2011).

## Supplementary Material

kiae287_Supplementary_Data

## Data Availability

All data generated during this study are included in this published article and its Supplemental Information files. The LCMS data were uploaded to ProteomeXchange using the Mass Spectrometry Interactive Virtual Environment (MassIVE) platform (Wang et al. 2018; https://massive.ucsd.edu/ProteoSAFe/static/massive.jsp). The dataset identifiers are PXD042254, PXD043420, and PXD047895.

## Supplementary data

The following materials are available in the online version of this article.

**Supplementary Table S1.** Compounds detected by gas chromatography–mass spectrometry (GC–MS) analysis in the extracellular vesicle (EV) samples and EV-depleted culture media of tissue-cultured cells of Norway spruce 7, 10, or 14 d after transfer of cells into liquid conditions.

**Supplementary Table S2.** Lignification-related compounds detected in the extracellular vesicle (EV) samples and the corresponding EV-depleted culture media of Norway spruce cells by ultra-performance liquid chromatography–mass spectrometry (UPLC–MS).

**Supplementary Table S3.** Proteins detected in the non-EDTA-extracted extracellular vesicle (EV) samples, EDTA-extracted EV samples, non-concentrated EV-depleted culture medium samples, and concentrated EV-depleted culture medium samples of Norway spruce under lignin-forming and non-lignin-forming conditions.

**Supplementary Figure S1.** Flowchart showing the process of isolating fractions from the tissue culture of Norway spruce that were analyzed in the current work.

**Supplementary Figure S2.** Extracellular vesicle (EV) samples stained with ruthenium red and by using the LM19 antibody in immunoblots; particle size distribution from a nanoparticle tracking analysis.

**Supplementary Figure S3.** Electron ionization (EI) mass spectra of the volatile derivatives of compounds detected by gas chromatography–mass spectrometry (GC–MS) analysis to be present in the extracellular vesicle (EV) samples and not in the corresponding medium samples.

**Supplementary Figure S4.** Evaluation of the presence of residual medium in the extracellular vesicle (EV) samples using 2 complementary strategies.

**Supplementary Figure S5.** Deconvoluted electron impact (EI) spectra of salicylic acid (SA) and SA isomers.

**Supplementary Figure S6.** REVIGO treemaps giving an overview of the 557 proteins detected in the EDTA-extracted extracellular vesicle (EV) samples isolated from the culture media of tissue-cultured Norway spruce cells.

**Supplementary Figure S7.** REVIGO treemaps giving an overview of the 72 proteins detected in the non-EDTA-extracted extracellular vesicle (EV) samples isolated from the culture media of tissue-cultured Norway spruce cells.

**Supplementary Figure S8.** Distribution of proteins in the EDTA-extracted extracellular vesicle (EV) samples and EV-depleted culture medium samples of Norway spruce.

**Supplementary Figure S9.** REVIGO treemaps giving an overview of the 218 proteins detected in extracellular vesicle (EV)-depleted, concentrated culture medium samples of tissue-cultured Norway spruce cells.

**Supplementary Figure S10.** REVIGO treemaps giving an overview of the 163 proteins common to concentrated, extracellular vesicle (EV)-depleted culture medium samples and EV samples of Norway spruce.

## Dive Curated Terms

The following phenotypic, genotypic, and functional terms are of significance to the work described in this paper:

DIR1 Gramene: AT5G48485
DIR1 Araport: AT5G48485

SKU5 Gramene: AT4G12420
SKU5 Araport: AT4G12420

TET8 Gramene: AT2G23810
TET8 Araport: AT2G23810

trans-resveratrol CHEBI: CHEBI:45713

EDTA Gramene: Ethylenediamine tetraacetic acid
EDTA Araport: Ethylenediamine tetraacetic acid

PEN1 Gramene: AT3G11820
PEN1 Araport: AT3G11820
