# Supplementary material for: Extracellular vesicles of Norway spruce contain precursors and enzymes for lignin formation and salicylic acid
Source: Plant Physiol. 2024 May 21;196(2):788–809. doi: 10.1093/plphys/kiae287 (PMC11444294; doi:10.1093/plphys/kiae287)
Supplement: kiae287_Supplementary_Data [file kiae287_supplementary_data.zip › Kankaanpaa_etal_Supplementary_Data.pdf]

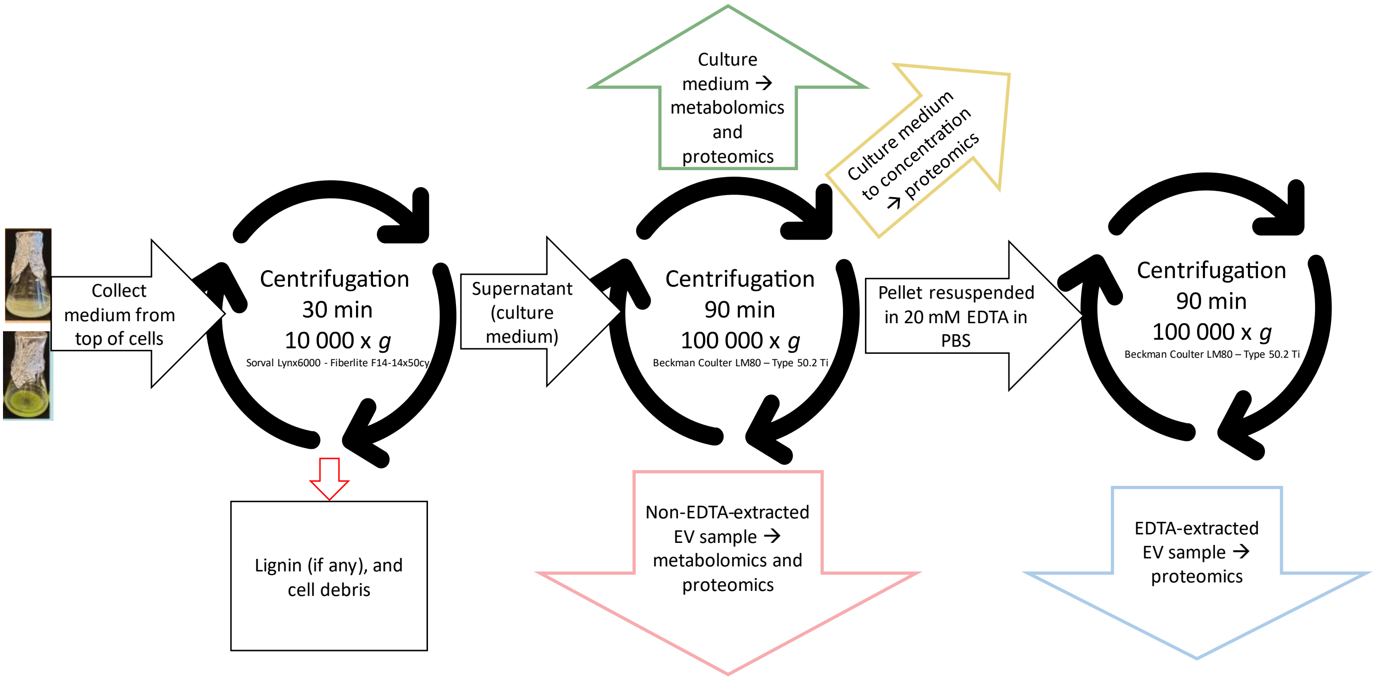

Supplementary Figure S1. Flowchart showing the process of isolating fractions from the tissue culture of Norway spruce that were analyzed in the current work.

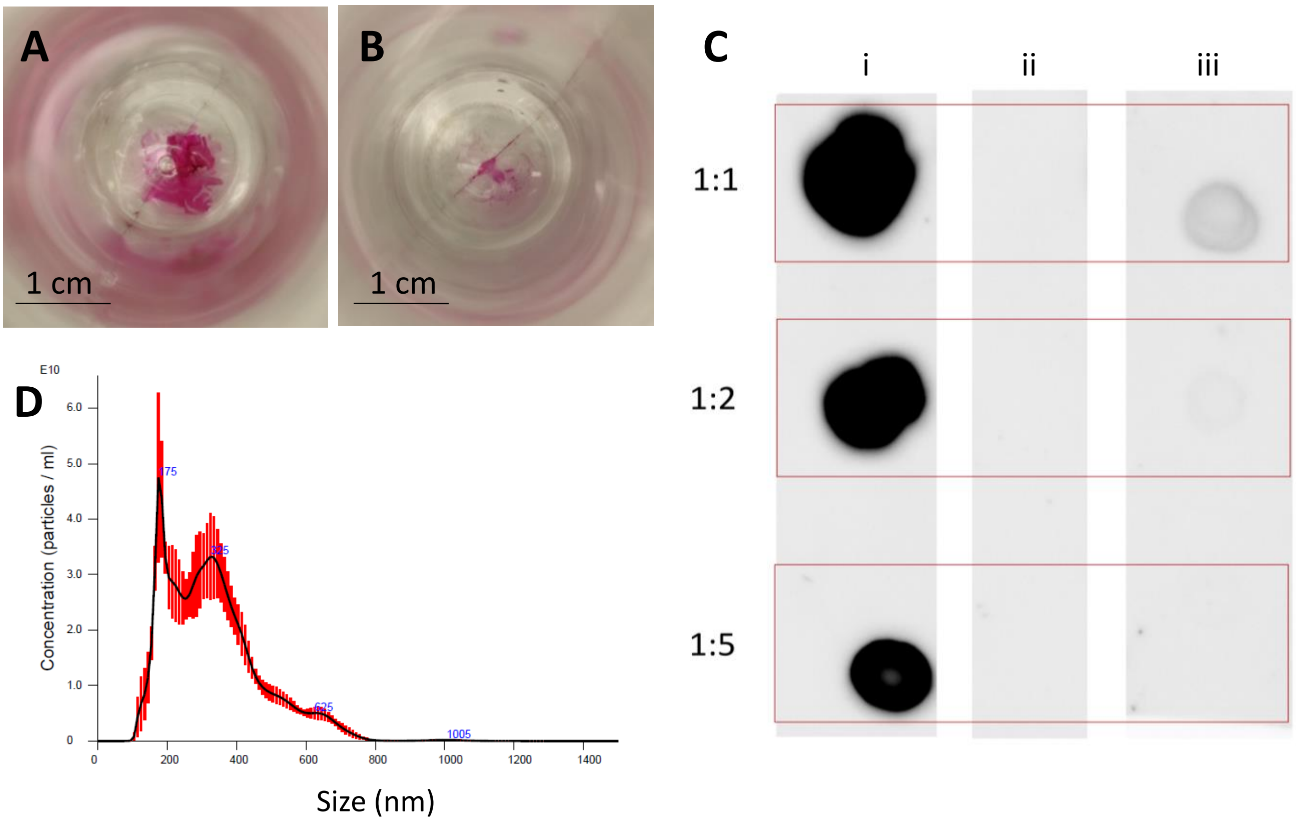

Supplementary Figure S2. Extracellular vesicle (EV) samples stained with Ruthenium Red and by using LM19 antibody in immunoblots; particle size distribution from a nanoparticle tracking analysis. A) Pelleted EV sample without EDTA treatment stained with Ruthenium Red. The dye stains negatively charged compounds including pectin and EV membranes red. **B)** EV pellet stained after EDTA extraction. Negatively-charged EV-membranes are slightly stained. **C)** Dot blot of EV samples without and with EDTA extraction solubilized in equal volumes of PBS using an LM19 antibody that recognizes weakly methylesterified homogalacturonan, and Alexa Fluor 647 goat anti-rat IgM as a secondary antibody. i) Non-EDTA extracted EV sample after first ultracentrifugation (15 µl/dot); ii) EDTA-extracted EV sample (15 µl/dot); iii) citrus pectin (100 µg/dot). The signal was detected as a fluorescence. **D)** Particle size distribution of EVs from a nanoparticle tracking analysis. Red bars denote the standard deviation across 5 replicates. Peaks identified are denoted with blue numbers.

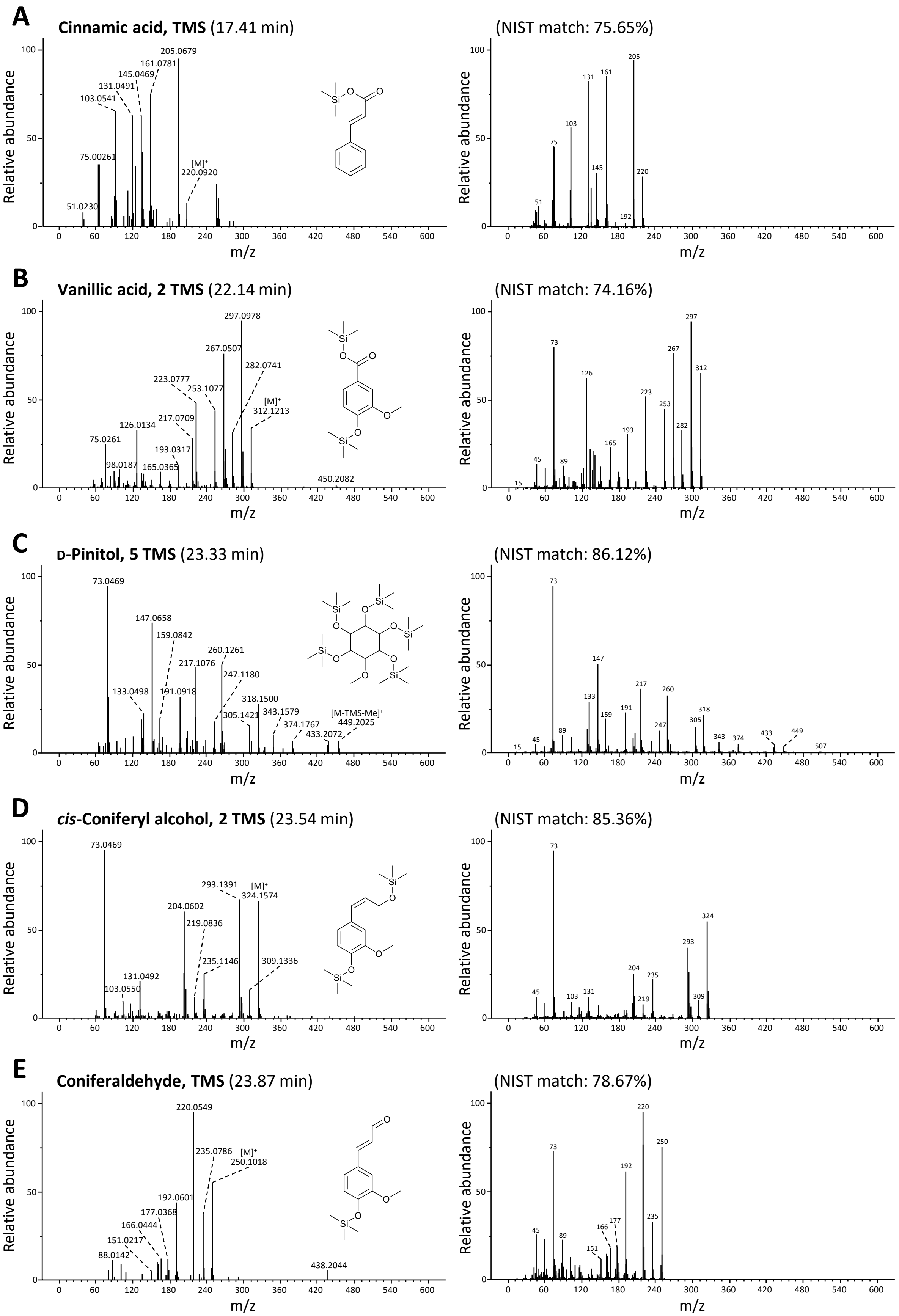
A
Cinnamic acid, TMS (17.41 min)
205.0679
161.0781
145.0469
131.0491
103.0541
75.00261
[M]+
220.0920
51.0230
(NIST match: 75.65%)
B
Vanillic acid, 2 TMS (22.14 min)
297.0978
267.0507
282.0741
223.0777
253.1077
[M]+
312.1213
217.0709
126.0134
75.0261
193.0317
98.0187
165.0365
450.2082
(NIST match: 74.16%)
C
D-Pinitol, 5 TMS (23.33 min)
73.0469
147.0658
159.0842
260.1261
217.1076
247.1180
133.0498
191.0918
318.1500
343.1579
[M-TMS-Me]+
305.1421
374.1767
449.2025
433.2072
(NIST match: 86.12%)
D
cis-Coniferyl alcohol, 2 TMS (23.54 min)
73.0469
293.1391
[M]+
324.1574
204.0602
219.0836
235.1146
309.1336
131.0492
103.0550
(NIST match: 85.36%)
E
Coniferaldehyde, TMS (23.87 min)
220.0549
235.0786
[M]+
250.1018
192.0601
177.0368
166.0444
151.0217
88.0142
438.2044
(NIST match: 78.67%)
Relative abundance
m/z

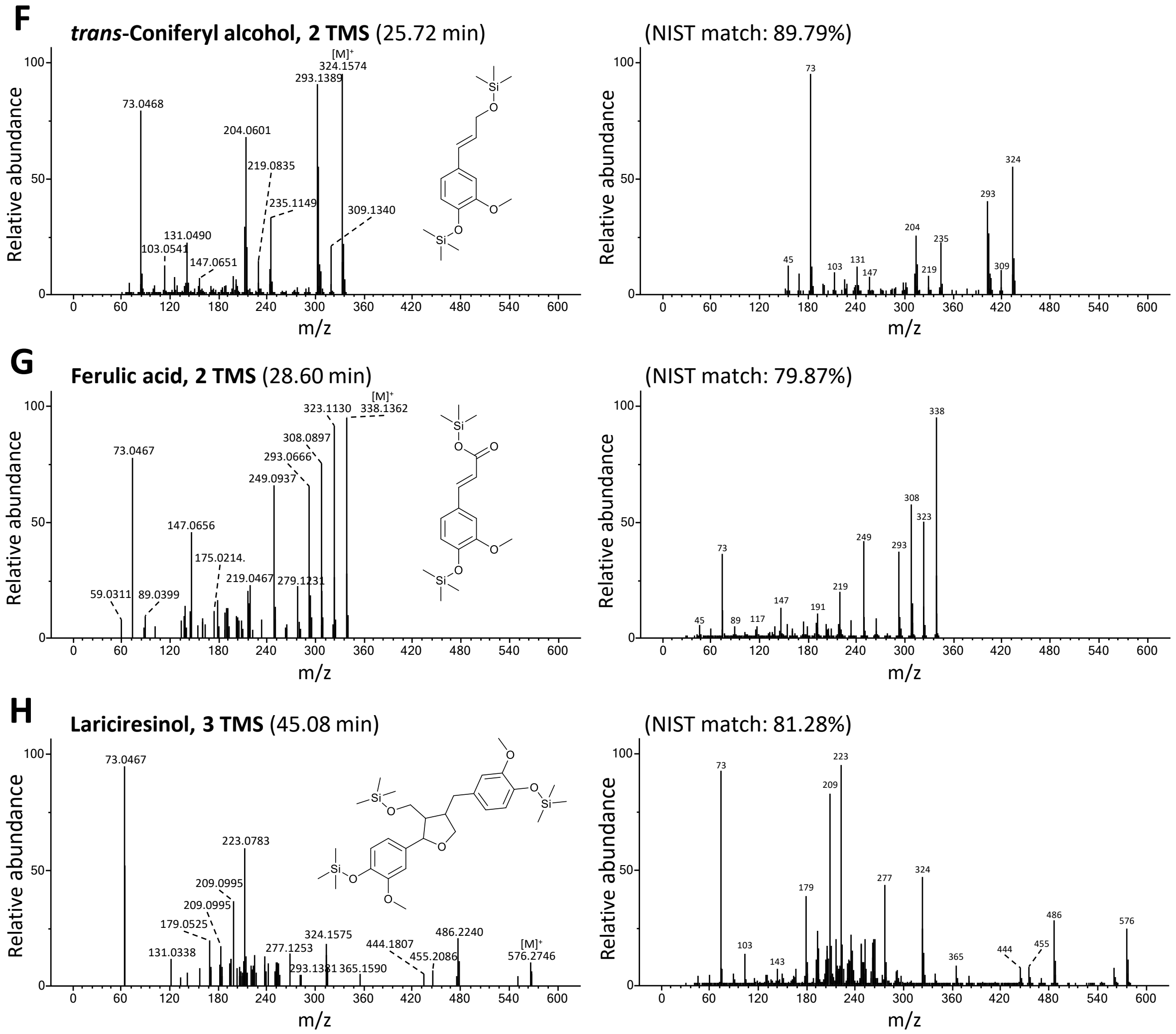

Supplementary Figure S3. Electron ionization (EI) mass spectra of volatile derivatives of compounds detected by gas chromatography–mass spectrometry (GC–MS) analysis to be present in extracellular vesicle (EV) samples, and not in the corresponding medium samples. A) Cinnamic acid; **B)** vanillic acid; **C)** D-pinitol; **D)** *cis*-coniferyl alcohol; **E)** coniferaldehyde; **F)** *trans*-coniferyl alcohol; **G)** ferulic acid; **H)** lariciresinol. Comparison spectra from the National Institute of Standards and Technology (NIST) mass spectral library are also shown on the right for each compound. For the spectrum of coniferyl alcohol at 23.54 min (panel **D**), similarities with the *trans*-coniferyl alcohol spectrum in the NIST mass spectral library are shown. Because (i) the EI-spectra of *cis*- and *trans*-configurations are typically highly similar, (ii) the EI-spectrum of *cis*-coniferyl alcohol is not part of the NIST mass spectral database, and (iii) *trans*-coniferyl alcohol is also detected at 25.72 min (panel **F**), we characterized the compound at 23.54 min as *cis*-coniferyl alcohol. TMS, trimethylsilyl.

7 days				10 days				14 days				+ KI 7 days				+ KI 10 days				+ KI 14 days				no.	metabolite name
0.18	-0.05	0.33	0.81	0.85	0.75	0.82	0.69	0.72	0.76	0.77	0.75	-0.09	0.04	-0.02	-0.05	0.18	0.06	0.16	0.30	0.84	0.73	0.86	0.88		correlation EV vs. medium sample
4.16	0.25	0.93	2.94	5.62	3.71	5.34	3.49	2.96	2.70	3.85	2.84	0.24	0.06	0.12	1.18	1.01	0.35	0.92	3.31	3.09	1.61	1.44	3.71	1	G(8-O-4)G 1
0.52	0.06	0.11	0.61	1.19	0.82	1.01	0.97	1.54	0.89	1.37	1.91	0.09	0.03	0.05	0.36	0.45	0.21	0.40	1.15	1.23	0.64	0.68	1.78	2	G(8-O-4)G 2
																								3	G(8-O-4)G 3
0.68	0.06	0.22	1.05	0.89	0.94	1.18	0.72	0.69	0.70	0.81	0.85	0.03	0.00	0.03	0.21	0.21	0.06	0.25	1.04	1.00	0.54	0.77	1.53	4	G(8-O-4)G(8-O-4)G 1
0.36	0.01	0.11	0.56	0.48	0.43	0.64	0.46	0.38	0.31	0.44	0.42	0.01	0.00	0.01	0.07	0.06	0.02	0.09	0.36	0.41	0.23	0.38	0.74	5	G(8-O-4)G(8-O-4)G 2
0.59	0.02	0.22	1.16	1.21	1.14	1.55	0.93	0.70	0.79	1.03	0.79	0.01	0.00	0.01	0.09	0.12	0.03	0.13	0.59	0.98	0.43	0.78	1.36	6	G(8-O-4)G(8-O-4)G 3
0.33	0.00	0.10	0.72	0.82	0.77	1.01	0.75	1.00	0.66	0.96	1.10	0.00	0.00	0.00	0.05	0.09	0.01	0.09	0.47	0.85	0.42	0.70	1.31	7	G(8-O-4)G(8-O-4)G 4
0.35	0.01	0.13	0.98	1.19	1.08	1.64	1.12	0.75	0.65	1.23	1.10	0.01	0.00	0.00	0.02	0.08	0.01	0.06	0.39	1.16	0.57	1.13	2.01	8	G(8-O-4)G(8-O-4)G 5
0.20	0.16	0.10	0.37	0.25	0.37	0.36	0.18	0.16	0.17	0.17	0.20	0.55	0.85	0.36	0.23	0.15	0.15	0.09	0.23	0.22	0.14	0.19	0.36	9	G(8-O-4)G(8-O-4)G(8-O-4)G 1
0.06	0.00	0.03	0.59	0.34	0.69	0.67	0.26	0.22	0.27	0.29	0.28	0.00	0.00	0.00	0.00	0.01	0.00	0.00	0.09	0.36	0.16	0.33	0.65	10	G(8-O-4)G(8-O-4)G(8-O-4)G 2
0.04	0.00	0.00	0.68	0.41	0.80	0.94	0.32	0.29	0.48	0.54	0.42	0.00	0.00	0.00	0.00	0.00	0.00	0.00	0.10	0.61	0.21	0.49	0.91	11	G(8-O-4)G(8-O-4)G(8-O-4)G 3
0.00	0.00	0.00	0.39	0.59	0.72	0.75	0.04	0.11	1.40	0.24	0.07	0.00	0.00	0.00	0.00	0.00	0.00	0.00	0.00	0.95	0.03	0.45	0.51	12	G(8-O-4)G(8-8)G
0.00	0.00	0.00	0.44	0.56	1.10	0.69	0.02	0.05	0.76	0.18	0.03	0.00	0.00	0.00	0.00	0.00	0.00	0.00	0.00	0.61	0.02	0.30	0.44	13	G(8-O-4)G(8-5)G
0.03	0.00	0.03	1.32	0.92	2.27	1.95	0.60	0.43	1.08	1.02	0.46	0.00	0.00	0.00	0.00	0.01	0.00	0.00	0.03	0.77	0.24	0.79	1.27	14	G(8-O-4)G(red8-5)G 1
0.00	0.00	0.00	0.70	0.82	1.56	1.42	0.10	0.23	1.26	0.68	0.12	0.00	0.00	0.00	0.00	0.00	0.00	0.00	0.00	1.30	0.07	0.79	1.12	15	G(8-O-4)G(red8-5)G 2
0.00	0.00	0.00	0.11	0.17	0.43	0.26	0.02	0.03	0.44	0.10	0.05	0.00	0.00	0.00	0.00	0.00	0.00	0.00	0.00	0.34	0.01	0.17	0.27	16	G(8-O-4)G(8-O-4)G(red8-5)G 2
4.28	0.51	1.29	5.04	7.35	3.00	8.35	4.97	4.09	3.22	3.80	3.09	0.24	0.07	0.10	1.29	2.04	0.40	1.39	4.61	4.30	2.53	1.79	4.82	17	G(8-O-4)dihydroG 1
0.98	0.19	0.22	0.95	1.60	1.15	1.36	1.12	1.01	0.85	1.07	1.05	0.16	0.05	0.08	0.79	0.85	0.37	0.73	2.29	1.49	0.98	0.80	2.39	18	G(8-O-4)dihydroG 2
0.22	0.02	0.06	0.29	0.28	0.28	0.31	0.25	0.28	0.27	0.28	0.34	0.01	0.00	0.00	0.11	0.17	0.05	0.15	0.65	0.43	0.27	0.30	0.85	19	G(8-O-4)G(8-O-4)dihydroG 1
0.49	0.04	0.13	0.62	0.68	0.56	0.73	0.57	0.58	0.45	0.54	0.56	0.03	0.01	0.02	0.15	0.19	0.05	0.16	0.70	0.75	0.38	0.54	1.34	20	G(8-O-4)G(8-O-4)dihydroG 2
0.64	0.04	0.18	1.03	1.15	1.08	1.43	1.16	0.86	0.77	0.95	1.00	0.03	0.01	0.02	0.12	0.17	0.05	0.17	0.80	0.99	0.45	0.80	1.72	21	G(8-O-4)G(8-O-4)dihydroG 3
0.13	0.00	0.03	0.41	0.30	0.50	0.48	0.21	0.29	0.28	0.32	0.38	0.00	0.00	0.00	0.01	0.01	0.00	0.01	0.19	0.39	0.17	0.29	0.62	22	G(8-O-4)G(8-O-4)G(8-O-4)dihydroG 1
0.25	0.20	0.21	0.86	0.66	0.42	0.86	1.57	2.27	1.24	46.7	2.58	0.23	0.08	0.09	0.80	0.77	0.51	0.46	2.69	1.47	0.70	0.5	3.23	23	G(8-O-4)7-hydroxydihydroG 1
0.71	0.40	0.30	2.11	2.03	1.13	2.17	3.24	10.3	6.10	120	10.9	0.36	0.17	0.18	1.90	1.52	1.19	1.20	4.30	4.1	1.76	2	7.2	24	G(8-O-4)7-hydroxydihydroG 2
0.04	0.00	0.01	0.12	0.05	0.05	0.08	0.08	0.07	0.07	5.41	0.11	0.00	0.00	0.00	0.05	0.11	0.18	0.04	0.35	0.18	0.08	0.06	0.35	25	G(8-O-4)G(8-O-4)7-hydroxydihydroG 1
0.02	0.00	0.01	0.10	0.03	0.05	0.06	0.05	0.07	0.05	0.03	0.10	0.00	0.00	0.00	0.05	0.07	0.01	0.04	0.26	0.14	0.07	0.06	0.29	26	G(8-O-4)G(8-O-4)7-hydroxydihydroG 2
0.06	0.02	0.02	0.14	0.08	0.08	0.11	0.09	0.12	0.09	0.15	0.18	0.01	0.00	0.00	0.09	0.10	0.07	0.06	0.38	0.22	0.10	0.10	0.41	27	G(8-O-4)G(8-O-4)7-hydroxydihydroG 3
0.07	0.02	0.03	0.17	0.13	0.11	0.15	0.12	0.16	0.11	0.19	0.17	0.00	0.00	0.00	0.09	0.10	0.05	0.05	0.41	0.28	0.11	0.14	0.40	28	G(8-O-4)G(8-O-4)7-hydroxydihydroG 4
0.13	0.04	0.02	0.14	0.15	0.09	0.18	0.20	0.28	0.15	0.62	0.33	0.12	0.04	0.04	0.19	0.15	0.21	0.09	0.39	0.23	0.32	0.09	0.48	29	G(8-O-4)7,8-dihydroxydihydroG
0.68	0.00	0.40	1.08	3.67	0.93	3.16	4.11	6.55	2.91	53.2	5.55	0.00	0.00	0.00	0.00	0.19	0.00	0.00	0.63	6.36	0.75	2.2	3.92	30	G(8-O-4)*p*-hydroxybenzyl alcohol
0.37	0.00	0.17	0.91	1.26	0.86	1.39	1.37	3.19	1.59	1.96	2.72	0.12	0.00	0.00	0.11	0.00	0.00	0.10	0.45	2.05	0.34	1.06	1.55	31	G(8-O-4)G(8-O-4)*p*-hydroxybenzyl alcohol
0.13	0.01	0.07	0.60	0.55	0.55	0.96	0.64	0.87	0.66	0.88	0.80	0.01	0.00	0.00	0.02	0.05	0.01	0.03	0.20	0.87	0.38	0.82	1.44	32	G(8-O-4)H(8-O-4)G
0.23	0.06	0.05	0.26	0.20	0.19	0.21	0.22	0.19	0.19	0.17	0.26	0.05	0.01	0.02	0.19	0.18	0.09	0.16	0.70	0.34	0.17	0.16	0.74	33	G(red8-5)G hexoside
1.05	0.12	0.29	1.15	2.25	1.33	1.88	1.24	1.05	1.10	1.36	1.43	0.07	0.01	0.02	0.30	0.65	0.12	0.37	1.45	1.96	0.78	1.16	2.89	34	G(8-8)G hexoside

Supplementary Figure S4. Evaluation of the presence of residual medium in the extracellular vesicle (EV) samples using two complementary strategies. First, the Pearson correlation coefficients between the peak intensities of 34 compounds detected in the EV samples and those in the corresponding medium samples were calculated and are shown in the first row. Correlation coefficients >0.6 (in yellow) are indicative for similarities in the relative abundancies of the compounds in the EV samples and the corresponding medium samples. The samples with correlation coefficients <0.6 are unlikely to suffer from contamination with the medium. A correlation coefficient >0.6 does not prove contamination with the medium, as there are several reasons that could cause similarities in the relative concentration of the metabolites in the EV fraction and the medium. Nevertheless, in case the correlation coefficient is larger than 0.6, a contamination cannot be ruled out. Second, for each of the 34 compounds detected in the EV samples, we calculated their relative abundance as compared to their abundance in the corresponding medium samples. The values are the outcome of the following equation: 100x(peak intensity in EV sample)/(peak intensity in medium sample). Compound **3** was below the detection limit in the medium, therefore no value is given for this compound. In addition, for each sample, the compound with the highest peak intensity in the corresponding medium sample is indicated with a black-framed box. We followed the reasoning that, in case a contamination of the EV samples with medium took place, then the compounds with the highest peak intensity in the medium samples (i.e., those in the black-framed box) are the most reliable to estimate the degree of contamination. To ease reading, we then color-coded the values of the 34 compounds in each sample as following: the ratio of the compound with the highest peak intensity in the corresponding medium sample (i.e., the value in the black-framed box) has a white background, values that are smaller are shown with a blue background, and values that are ≥2 the value in the black-framed box are shown with a red background and are written in bold. The compounds with numbers in bold on the red background are relatively enriched in the EV samples and thus unlikely to be caused by contamination with the medium.

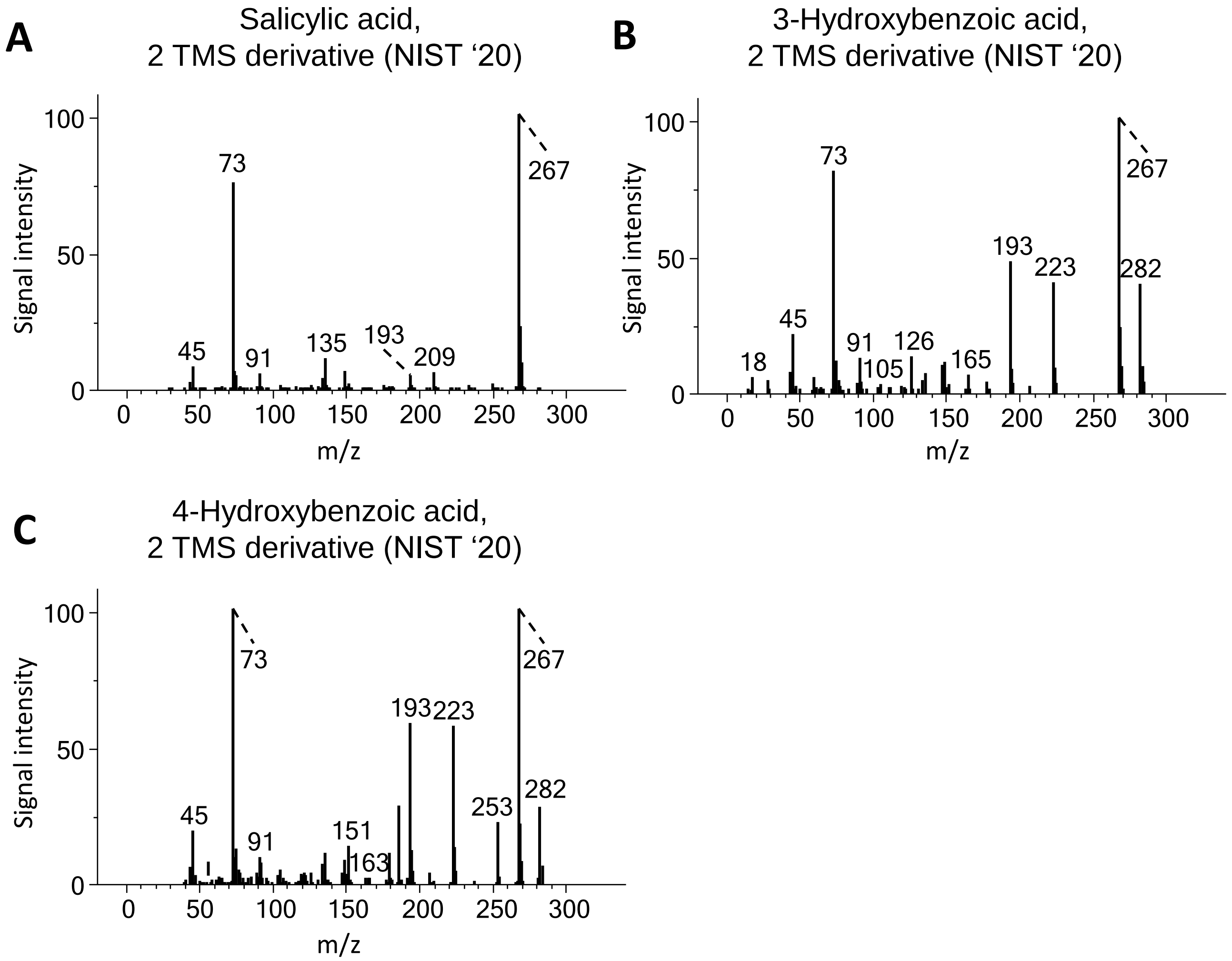

Supplementary Figure S5. **Deconvoluted electron impact (EI) spectra of salicylic acid (SA), and SA isomers. A)** EI spectrum of the 2 trimethylsilyl (TMS) derivative of SA in the National Institute of Standards and Technology (NIST) ‘20 spectral library. **B)** EI spectrum of the 2 TMS derivative of 3-hydroxybenzoic acid in the NIST ‘20 spectral library. **C)** EI spectrum of the 2 TMS derivative of 4-hydroxybenzoic acid in the NIST ‘20 spectral library.

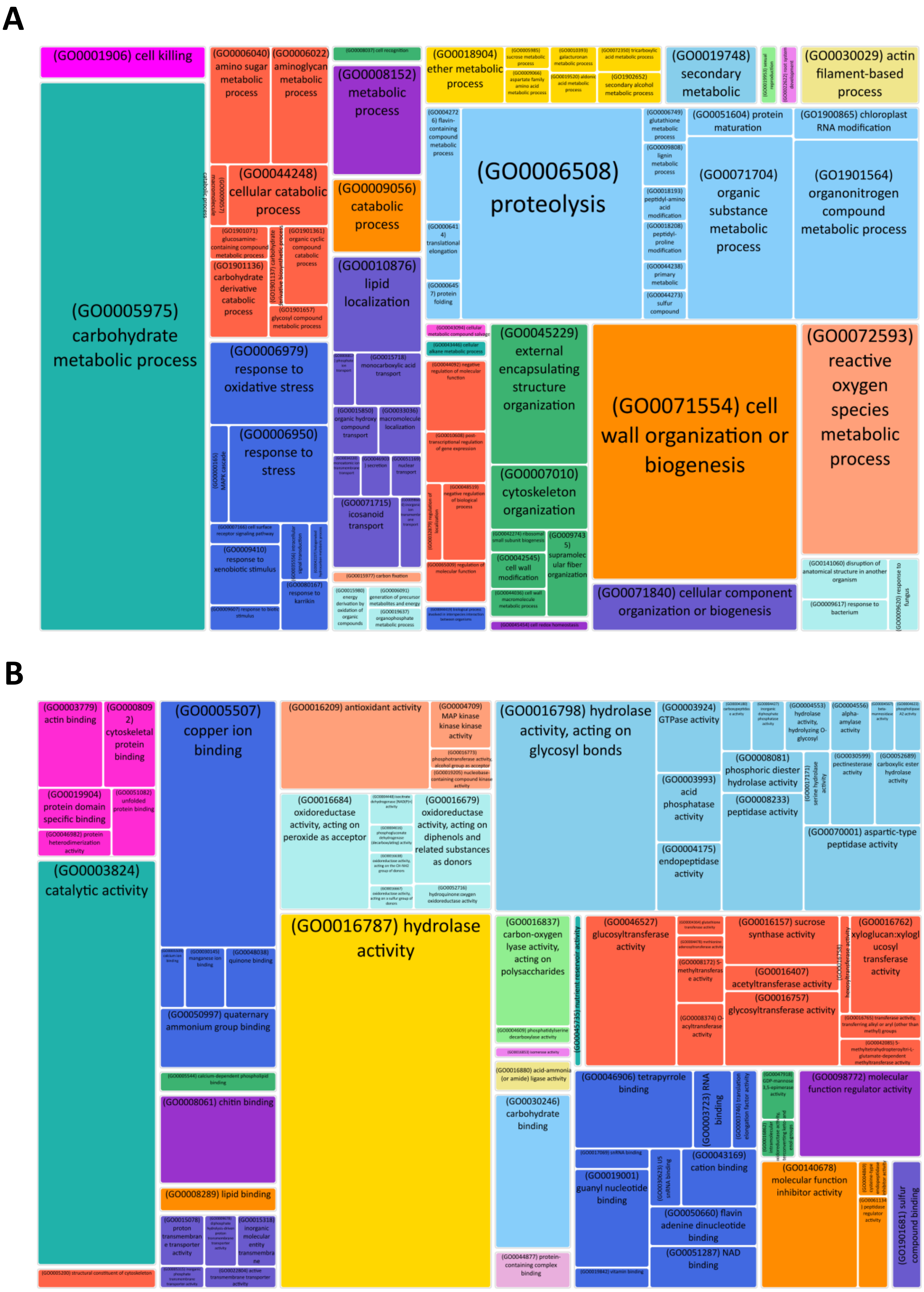
A
(GO0001906) cell killing
(GO0005975) carbohydrate metabolic process
(GO0006040) amino sugar metabolic process
(GO0006022) aminoglycan metabolic process
(GO0008152) metabolic process
(GO0018904) ether metabolic process
(GO0019748) secondary metabolic
(GO0030029) actin filament-based process
(GO0044248) cellular catabolic process
(GO0009056) catabolic process
(GO0010876) lipid localization
(GO0006508) proteolysis
(GO0051604) protein maturation
(GO1900865) chloroplast RNA modification
(GO0071704) organic substance metabolic process
(GO1901564) organonitrogen compound metabolic process
(GO1901136) carbohydrate derivative catabolic process
(GO0006979) response to oxidative stress
(GO0006950) response to stress
(GO0009410) response to xenobiotic stimulus
(GO0071715) icosanoid transport
(GO0045229) external encapsulating structure organization
(GO0007010) cytoskeleton organization
(GO0071554) cell wall organization or biogenesis
(GO0072593) reactive oxygen species metabolic process
(GO0071840) cellular component organization or biogenesis
(GO0042545) cell wall modification
B
(GO0003779) actin binding
(GO0008092) cytoskeletal protein binding
(GO0019904) protein domain specific binding
(GO0003824) catalytic activity
(GO0005507) copper ion binding
(GO0050997) quaternary ammonium group binding
(GO0008061) chitin binding
(GO0008289) lipid binding
(GO0016209) antioxidant activity
(GO0004709) MAP kinase kinase kinase activity
(GO0016684) oxidoreductase activity, acting on peroxide as acceptor
(GO0016679) oxidoreductase activity, acting on diphenols and related substances as donors
(GO0016787) hydrolase activity
(GO0016798) hydrolase activity, acting on glycosyl bonds
(GO0003924) GTPase activity
(GO0003993) acid phosphatase activity
(GO0004175) endopeptidase activity
(GO0008081) phosphoric diester hydrolase activity
(GO0008233) peptidase activity
(GO0070001) aspartic-type peptidase activity
(GO0016837) carbon-oxygen lyase activity, acting on polysaccharides
(GO0046527) glucosyltransferase activity
(GO0016157) sucrose synthase activity
(GO0016407) acetyltransferase activity
(GO0016757) glycosyltransferase activity
(GO0016762) xyloglucan:xyloglucosyl transferase activity
(GO0030246) carbohydrate binding
(GO0046906) tetrapyrrole binding
(GO0003723) RNA binding
(GO0098772) molecular function regulator activity
(GO0019001) guanyl nucleotide binding
(GO0043169) cation binding
(GO0050660) flavin adenine dinucleotide binding
(GO0051287) NAD binding
(GO0140678) molecular function inhibitor activity
(GO1901681) sulfur compound binding

C

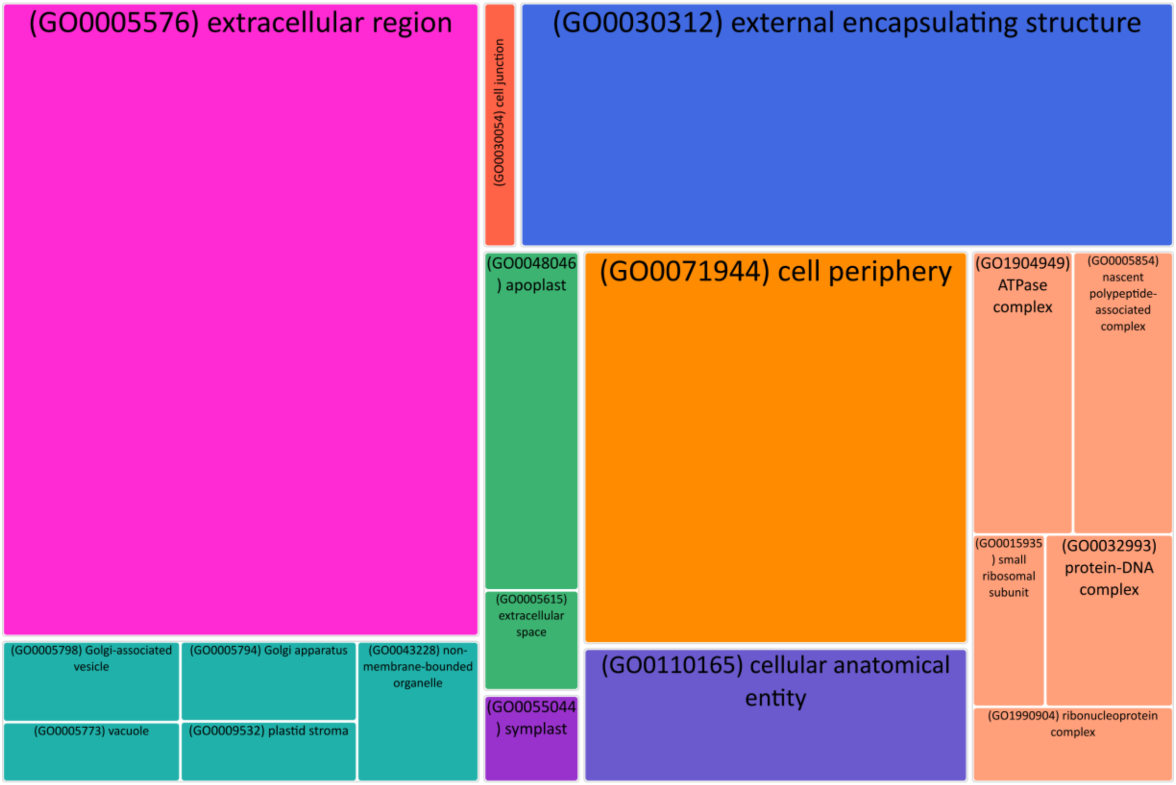

Supplementary Figure S6. REVIGO treemaps giving an overview of the 557 proteins detected in the EDTA-extracted extracellular vesicle (EV) samples isolated from the culture media of tissue-cultured Norway spruce cells. A) Biological function, **B)** molecular function, and **C)** cellular component of the proteins detected. Co-pelleted pectin was extracted away from the EV-enriched pellets with an EDTA-containing buffer before protein analysis.

A

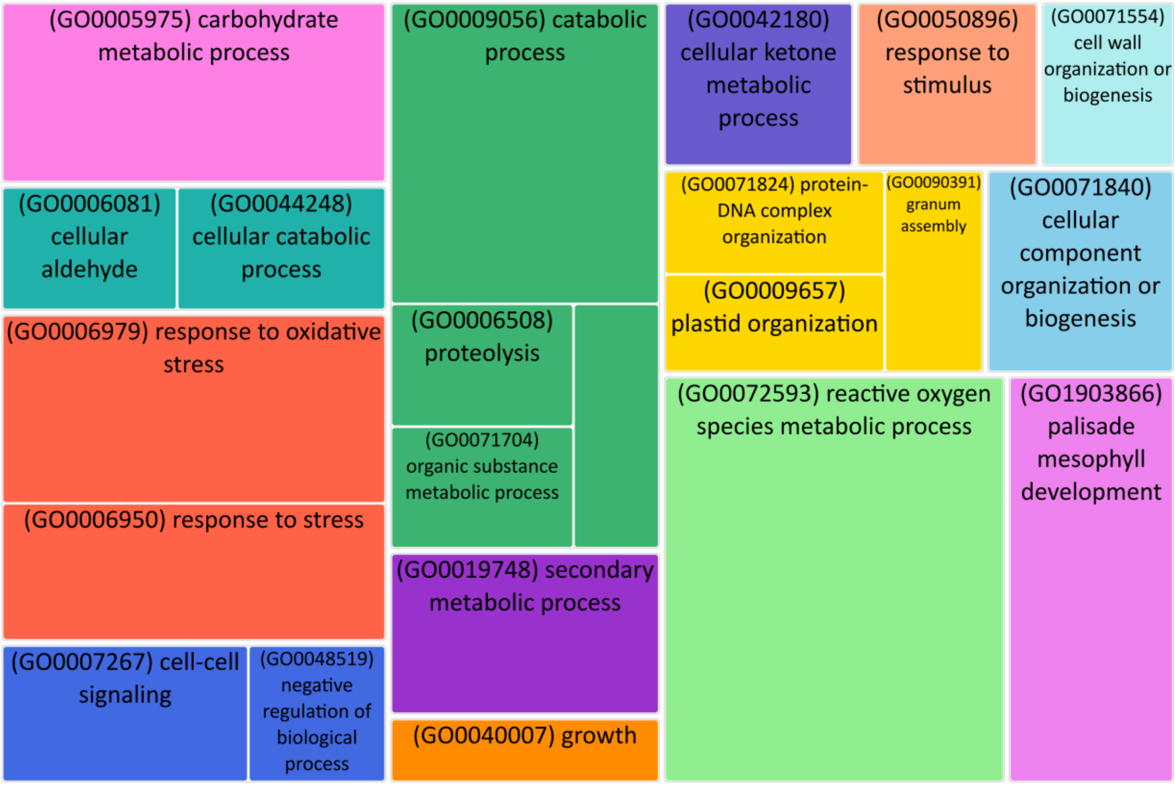

(GO0005975) carbohydrate metabolic process
(GO0009056) catabolic process
(GO0042180) cellular ketone metabolic process
(GO0050896) response to stimulus
(GO0071554) cell wall organization or biogenesis
(GO0006081) cellular aldehyde
(GO0044248) cellular catabolic process
(GO0071824) protein-DNA complex organization
(GO0090391) granum assembly
(GO0071840) cellular component organization or biogenesis
(GO0009657) plastid organization
(GO0006979) response to oxidative stress
(GO0006508) proteolysis
(GO0071704) organic substance metabolic process
(GO0072593) reactive oxygen species metabolic process
(GO1903866) palisade mesophyll development
(GO0006950) response to stress
(GO0019748) secondary metabolic process
(GO0007267) cell-cell signaling
(GO0048519) negative regulation of biological process
(GO0040007) growth

B

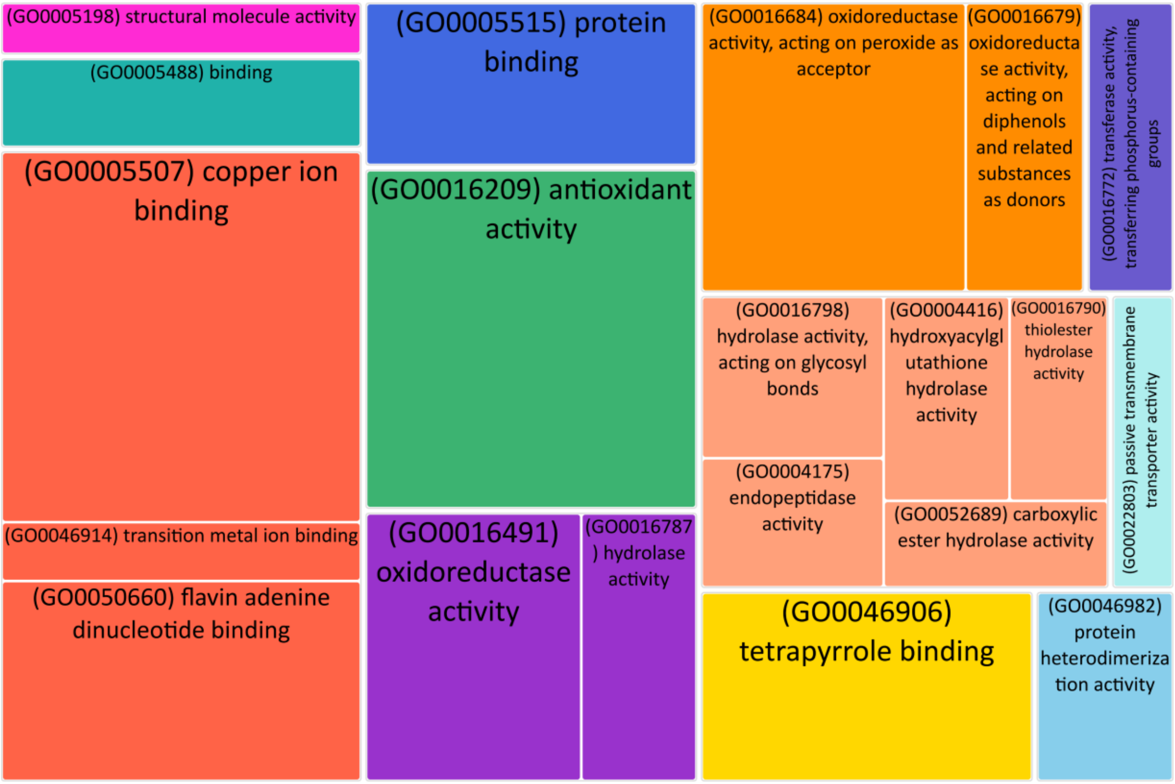

(GO0005198) structural molecule activity
(GO0005488) binding
(GO0005515) protein binding
(GO0016684) oxidoreductase activity, acting on peroxide as acceptor
(GO0016679) oxidoreductase activity, acting on diphenols and related substances as donors
(GO0016772) transferase activity, transferring phosphorus-containing groups
(GO0005507) copper ion binding
(GO0016209) antioxidant activity
(GO0016798) hydrolase activity, acting on glycosyl bonds
(GO0004416) hydroxyacylglutathione hydrolase activity
(GO0016790) thiolester hydrolase activity
(GO0022803) passive transmembrane transporter activity
(GO0004175) endopeptidase activity
(GO0052689) carboxylic ester hydrolase activity
(GO0046914) transition metal ion binding
(GO0016491) oxidoreductase activity
(GO0016787) hydrolase activity
(GO0050660) flavin adenine dinucleotide binding
(GO0046906) tetrapyrrole binding
(GO0046982) protein heterodimerization activity

C

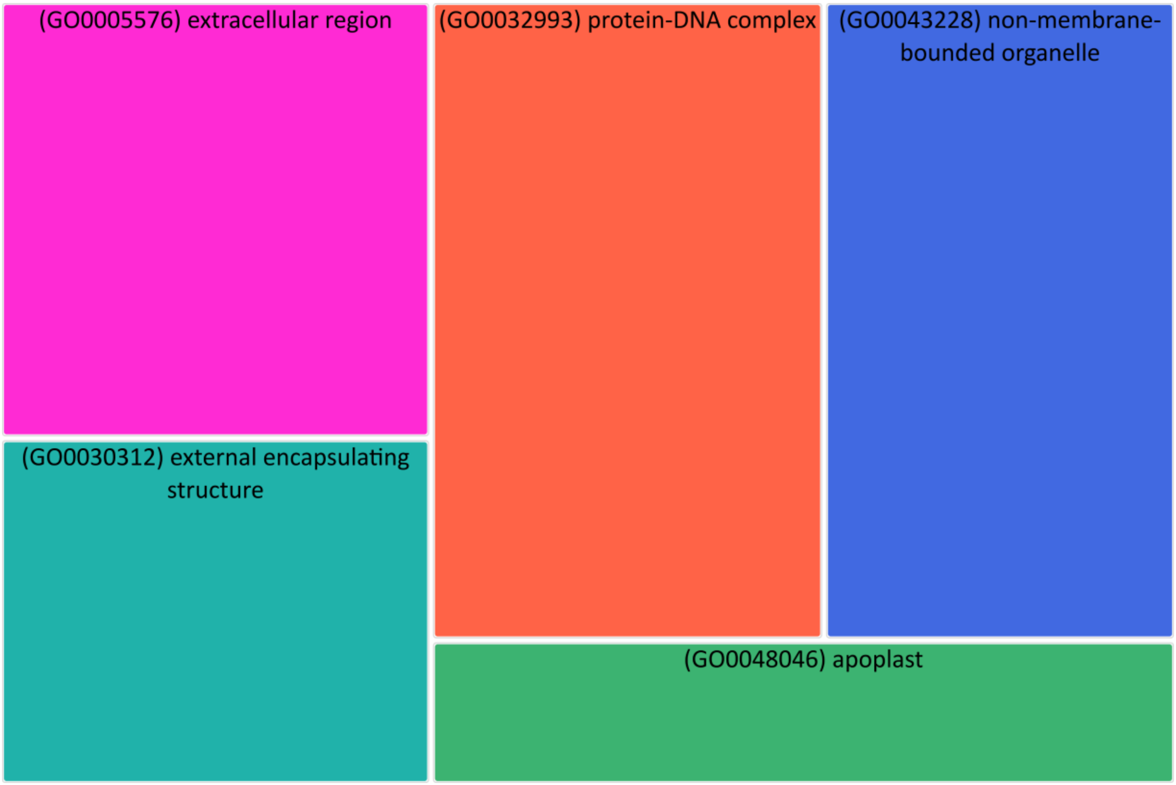

Supplementary Figure S7. REVIGO treemaps giving an overview of the 72 proteins detected in non-EDTA extracted extracellular vesicle (EV) samples isolated from the culture media of tissue-cultured Norway spruce cells. These proteins were additional to those detected in the EDTA-extracted EV samples and are putatively bound to pectin. **A)** Biological function, **B)** molecular function and **C)** cellular component of the proteins detected.

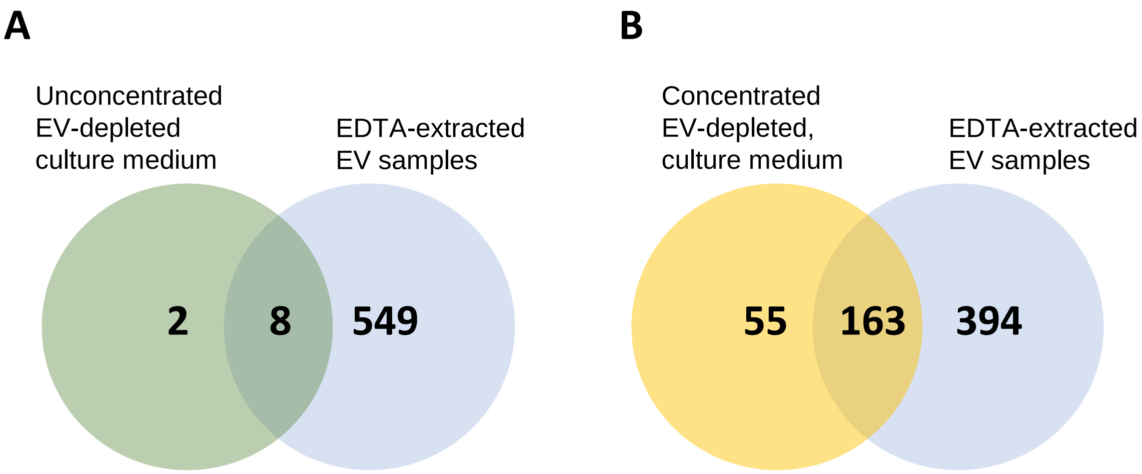

Supplementary Figure S8. Distribution of proteins in EDTA-extracted extracellular vesicle (EV) samples and EV-depleted culture medium samples of Norway spruce. A) A Venn diagram showing protein distribution in the unconcentrated EV-depleted culture medium and in the EDTA-extracted EV samples. **B)** Overall distribution of proteins in the EDTA-extracted EV samples and the concentrated culture medium.

A

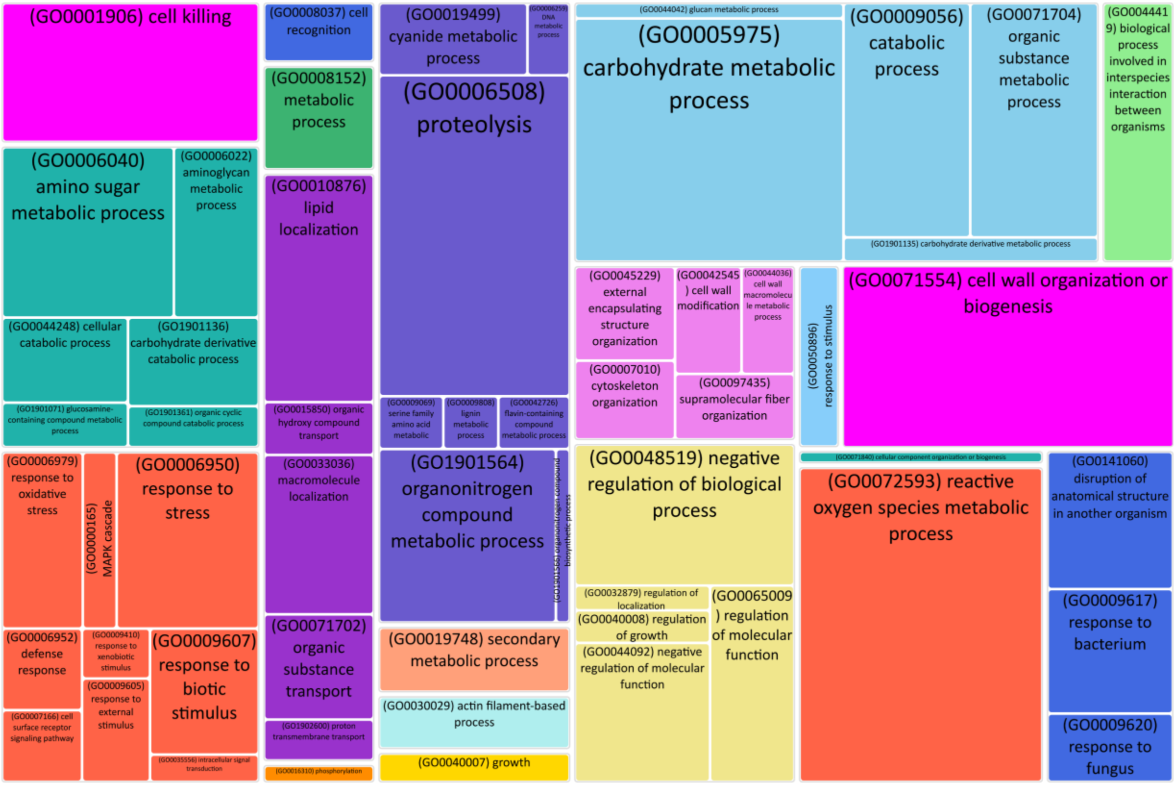
(GO0001906) cell killing
(GO0008037) cell recognition
(GO0019499) cyanide metabolic process
(GO0044042) glucan metabolic process
(GO0005975) carbohydrate metabolic process
(GO0009056) catabolic process
(GO0071704) organic substance metabolic process
(GO0044419) biological process involved in interspecies interaction between organisms
(GO0008152) metabolic process
(GO0006508) proteolysis
(GO0006040) amino sugar metabolic process
(GO0006022) aminoglycan metabolic process
(GO0010876) lipid localization
(GO1901135) carbohydrate derivative metabolic process
(GO0045229) external encapsulating structure organization
(GO0042545) cell wall modification
(GO0044036) cell wall macromolecule metabolic process
(GO0050896) response to stimulus
(GO0071554) cell wall organization or biogenesis
(GO0044248) cellular catabolic process
(GO1901136) carbohydrate derivative catabolic process
(GO0007010) cytoskeleton organization
(GO0097435) supramolecular fiber organization
(GO1901071) glucosamine-containing compound metabolic process
(GO1901361) organic cyclic compound catabolic process
(GO0015850) organic hydroxy compound transport
(GO0009069) serine family amino acid metabolic
(GO0009808) lignin metabolic process
(GO0042726) flavin-containing compound metabolic process
(GO0006979) response to oxidative stress
(GO0000165) MAPK cascade
(GO0006950) response to stress
(GO0033036) macromolecule localization
(GO1901564) organonitrogen compound metabolic process
(GO0048519) negative regulation of biological process
(GO0072593) reactive oxygen species metabolic process
(GO0141060) disruption of anatomical structure in another organism
(GO0032879) regulation of localization
(GO0040008) regulation of growth
(GO0065009) regulation of molecular function
(GO0044092) negative regulation of molecular function
(GO0009617) response to bacterium
(GO0006952) defense response
(GO0009410) response to xenobiotic stimulus
(GO0009607) response to biotic stimulus
(GO0071702) organic substance transport
(GO0019748) secondary metabolic process
(GO0009605) response to external stimulus
(GO0007166) cell surface receptor signaling pathway
(GO0030029) actin filament-based process
(GO1902600) proton transmembrane transport
(GO0035556) intracellular signal transduction
(GO0016310) phosphorylation
(GO0040007) growth
(GO0009620) response to fungus

B

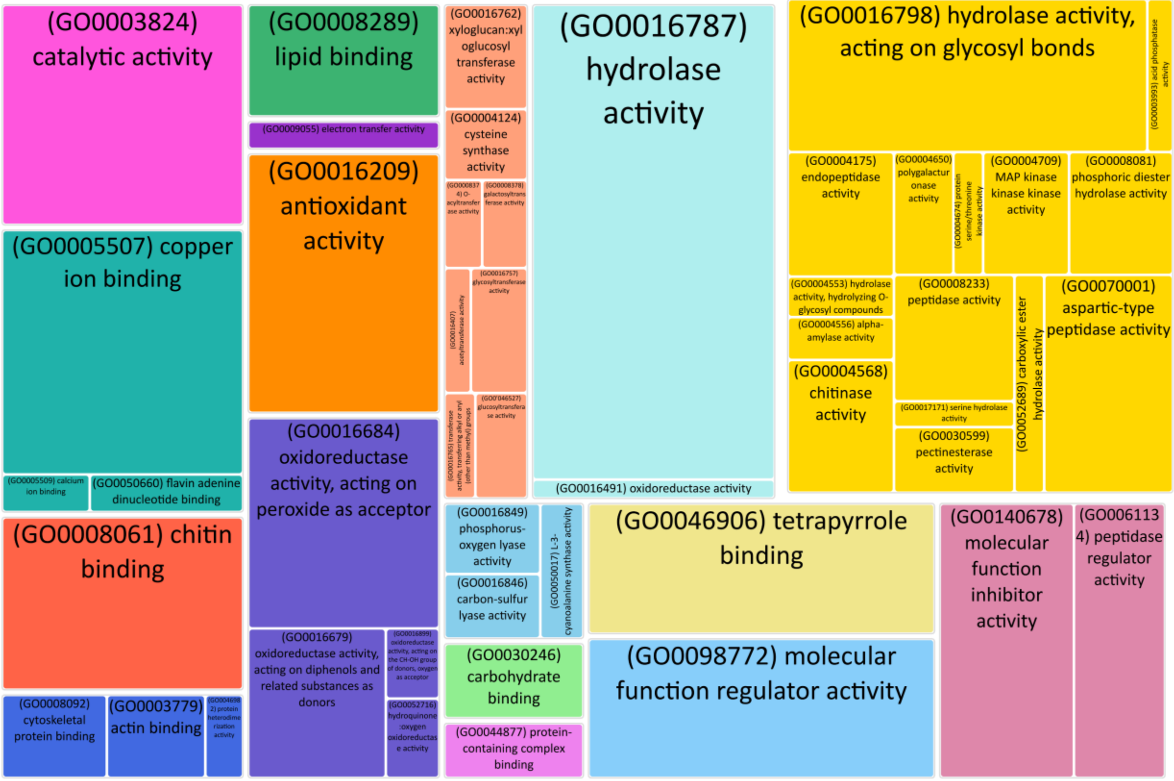
(GO0003824) catalytic activity
(GO0008289) lipid binding
(GO0016762) xyloglucan:xyloglucosyl transferase activity
(GO0016787) hydrolase activity
(GO0016798) hydrolase activity, acting on glycosyl bonds
(GO0003993) acid phosphatase activity
(GO0009055) electron transfer activity
(GO0004124) cysteine synthase activity
(GO0016209) antioxidant activity
(GO0004175) endopeptidase activity
(GO0004650) polygalacturonase activity
(GO0004674) protein serine/threonine kinase activity
(GO0004709) MAP kinase kinase kinase activity
(GO0008081) phosphoric diester hydrolase activity
(GO0005507) copper ion binding
(GO0008378) galactosyltransferase activity
(GO0016757) glycosyltransferase activity
(GO0016407) acetyltransferase activity
(GO0004553) hydrolase activity, hydrolyzing O-glycosyl compounds
(GO0004556) alpha-amylase activity
(GO0008233) peptidase activity
(GO0052689) carboxylic ester hydrolase activity
(GO0070001) aspartic-type peptidase activity
(GO0004568) chitinase activity
(GO0016765) transferase activity, transferring alkyl or aryl (other than methyl) groups
(GO0046527) glucosyltransferase activity
(GO0017171) serine hydrolase activity
(GO0016684) oxidoreductase activity, acting on peroxide as acceptor
(GO0030599) pectinesterase activity
(GO0005509) calcium ion binding
(GO0050660) flavin adenine dinucleotide binding
(GO0016491) oxidoreductase activity
(GO0008061) chitin binding
(GO0016849) phosphorus-oxygen lyase activity
(GO0050017) L-3-cyanoalanine synthase activity
(GO0046906) tetrapyrrole binding
(GO0140678) molecular function inhibitor activity
(GO0061134) peptidase regulator activity
(GO0016846) carbon-sulfur lyase activity
(GO0016679) oxidoreductase activity, acting on diphenols and related substances as donors
(GO0016899) oxidoreductase activity, acting on the CH-OH group of donors, oxygen as acceptor
(GO0030246) carbohydrate binding
(GO0098772) molecular function regulator activity
(GO0008092) cytoskeletal protein binding
(GO0003779) actin binding
(GO0052716) hydroquinone:oxygen oxidoreductase activity
(GO0044877) protein-containing complex binding

C

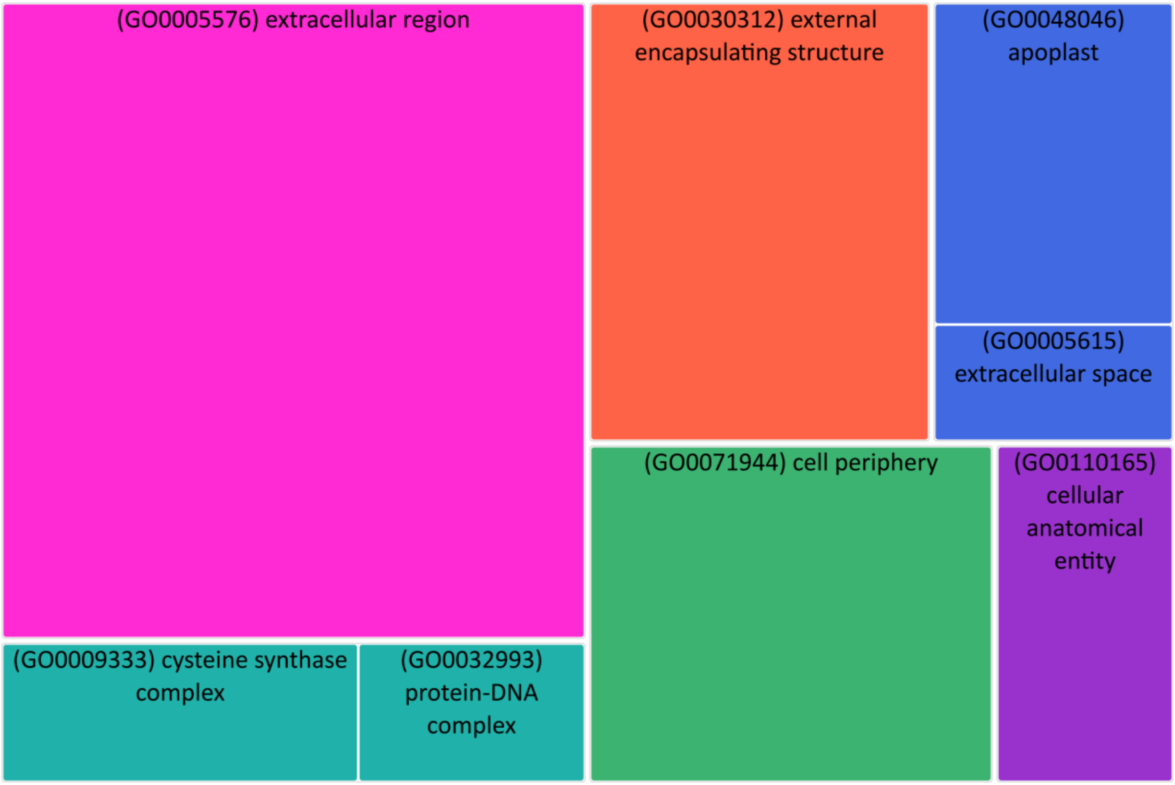

Supplementary Figure S9. REVIGO treemaps giving an overview of the 218 proteins detected in extracellular vesicle (EV)-depleted, concentrated culture medium samples of tissue-cultured Norway spruce cells. A) Cellular component, **B)** molecular function, **C)** cellular component of the proteins detected.

A

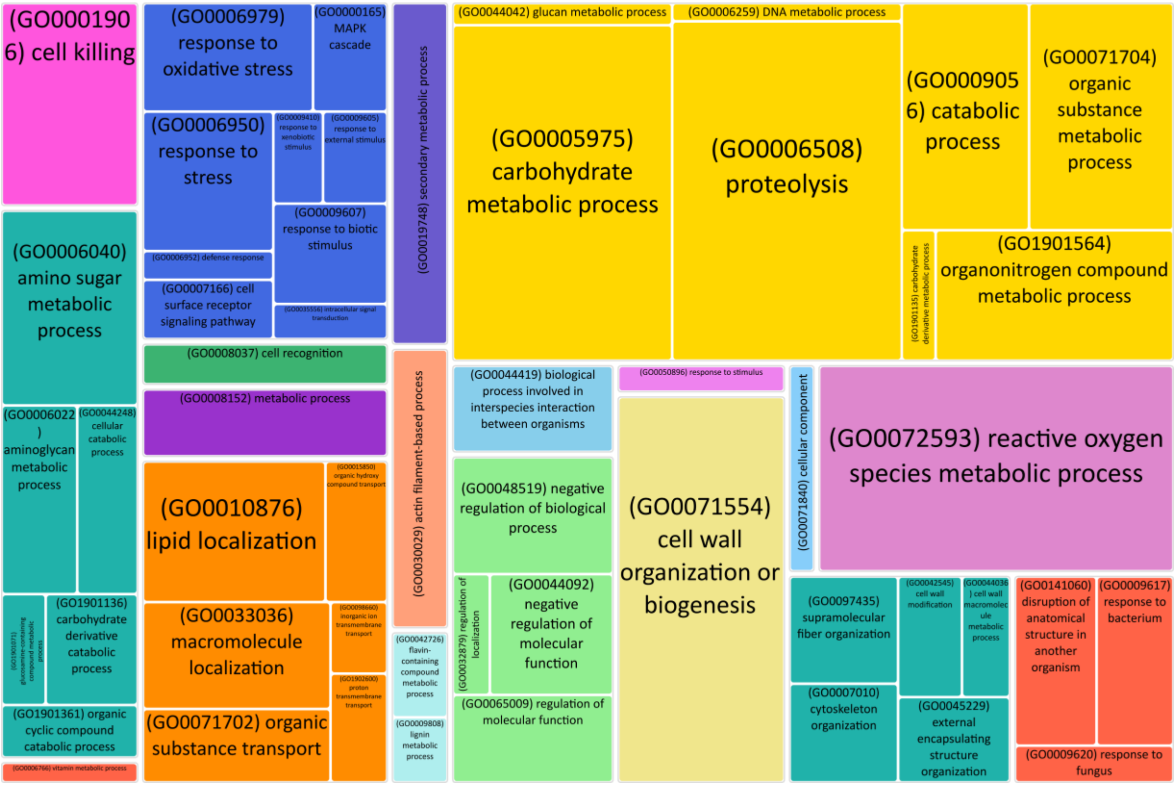
(GO0001906) cell killing
(GO0006979) response to oxidative stress
(GO0000165) MAPK cascade
(GO0019748) secondary metabolic process
(GO0044042) glucan metabolic process
(GO0006259) DNA metabolic process
(GO0005975) carbohydrate metabolic process
(GO0006508) proteolysis
(GO0009056) catabolic process
(GO0071704) organic substance metabolic process
(GO0006950) response to stress
(GO0009607) response to biotic stimulus
(GO0006040) amino sugar metabolic process
(GO0007166) cell surface receptor signaling pathway
(GO1901564) organonitrogen compound metabolic process
(GO1901135) carbohydrate derivative metabolic process
(GO0008037) cell recognition
(GO0008152) metabolic process
(GO0044419) biological process involved in interspecies interaction between organisms
(GO0050896) response to stimulus
(GO0071840) cellular component
(GO0072593) reactive oxygen species metabolic process
(GO0006022) aminoglycan metabolic process
(GO0044248) cellular catabolic process
(GO0010876) lipid localization
(GO0030029) actin filament-based process
(GO0048519) negative regulation of biological process
(GO0071554) cell wall organization or biogenesis
(GO1901136) carbohydrate derivative catabolic process
(GO0033036) macromolecule localization
(GO0032879) regulation of localization
(GO0044092) negative regulation of molecular function
(GO0097435) supramolecular fiber organization
(GO0141060) disruption of anatomical structure in another organism
(GO0009617) response to bacterium
(GO0042726) flavin-containing compound metabolic process
(GO0009808) lignin metabolic process
(GO0065009) regulation of molecular function
(GO0007010) cytoskeleton organization
(GO0045229) external encapsulating structure organization
(GO1901361) organic cyclic compound catabolic process
(GO0071702) organic substance transport
(GO0009620) response to fungus

B

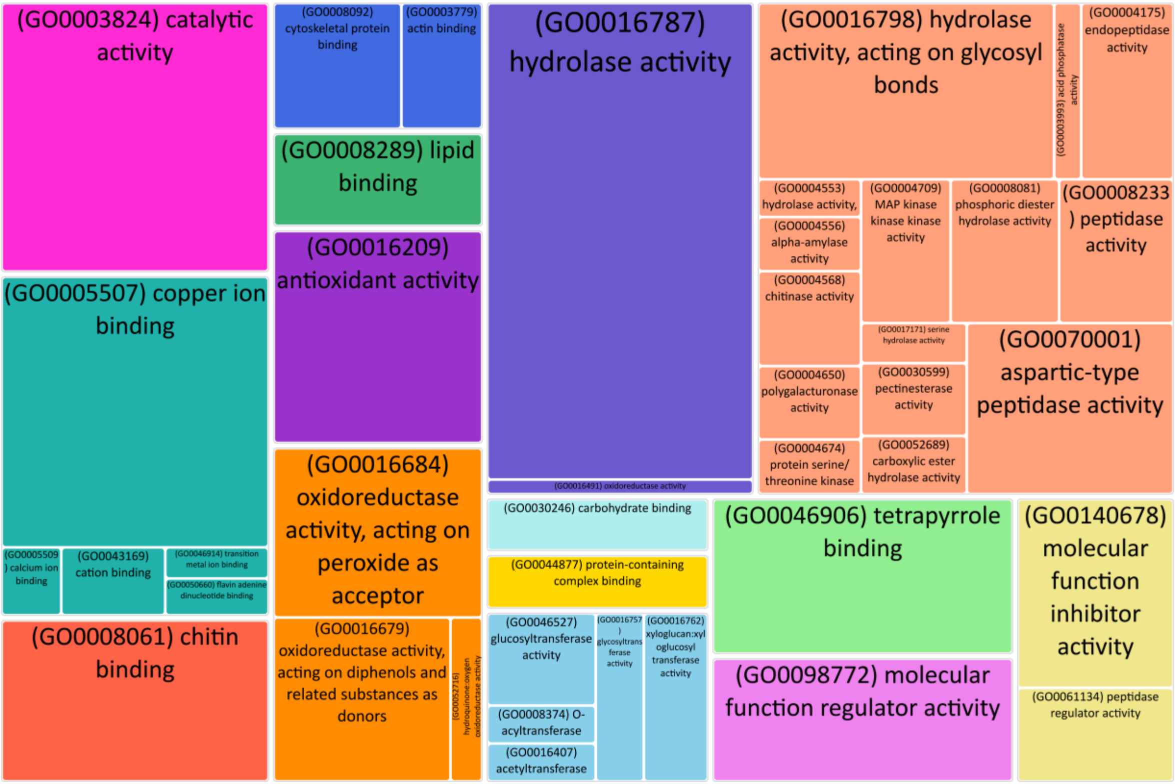
(GO0003824) catalytic activity
(GO0008092) cytoskeletal protein binding
(GO0003779) actin binding
(GO0016787) hydrolase activity
(GO0016798) hydrolase activity, acting on glycosyl bonds
(GO0003993) acid phosphatase activity
(GO0004175) endopeptidase activity
(GO0008289) lipid binding
(GO0004553) hydrolase activity,
(GO0004709) MAP kinase kinase kinase activity
(GO0008081) phosphoric diester hydrolase activity
(GO0008233) peptidase activity
(GO0016209) antioxidant activity
(GO0005507) copper ion binding
(GO0004556) alpha-amylase activity
(GO0004568) chitinase activity
(GO0070001) aspartic-type peptidase activity
(GO0004650) polygalacturonase activity
(GO0030599) pectinesterase activity
(GO0004674) protein serine/threonine kinase
(GO0052689) carboxylic ester hydrolase activity
(GO0016491) oxidoreductase activity
(GO0030246) carbohydrate binding
(GO0046906) tetrapyrrole binding
(GO0140678) molecular function inhibitor activity
(GO0016684) oxidoreductase activity, acting on peroxide as acceptor
(GO0005509) calcium ion binding
(GO0043169) cation binding
(GO0044877) protein-containing complex binding
(GO0008061) chitin binding
(GO0016679) oxidoreductase activity, acting on diphenols and related substances as donors
(GO0046527) glucosyltransferase activity
(GO0016762) xyloglucan:xyloglucosyl transferase activity
(GO0098772) molecular function regulator activity
(GO0061134) peptidase regulator activity
(GO0008374) O-acyltransferase
(GO0016407) acetyltransferase

C

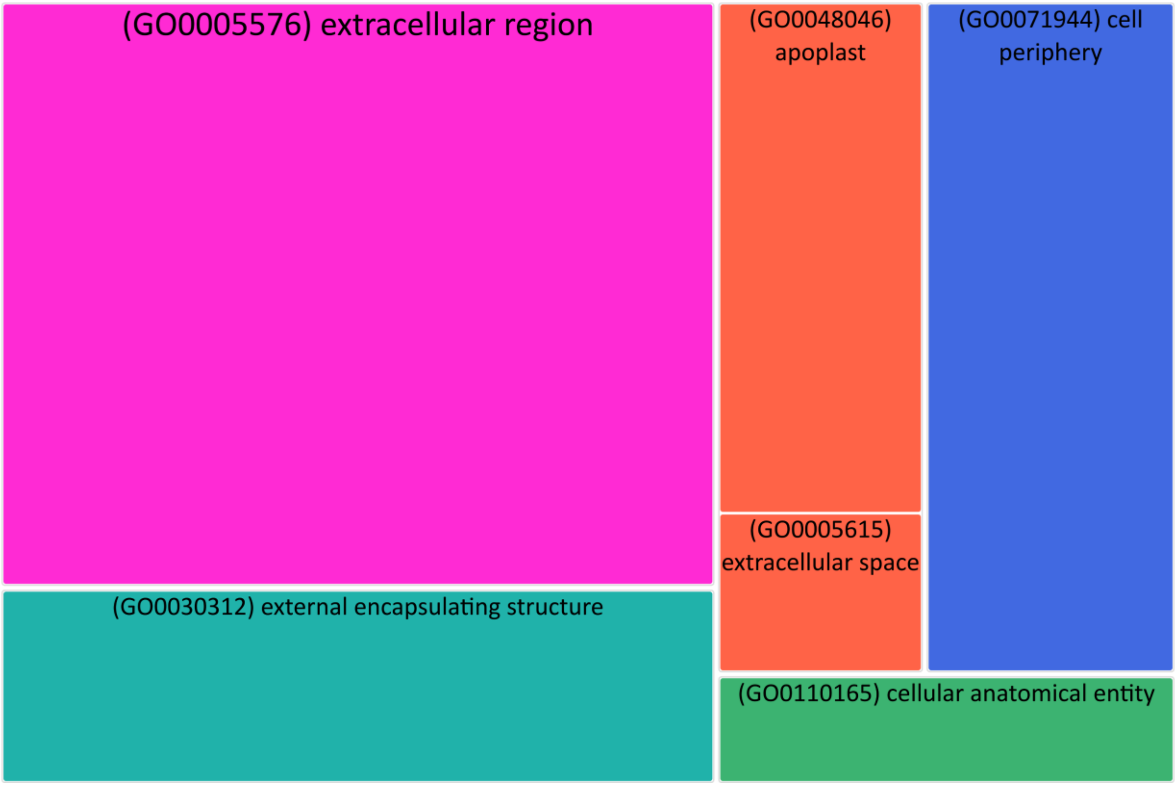

Supplementary Figure S10. REVIGO treemaps giving an overview of the 163 proteins common to concentrated, extracellular vesicle (EV)-depleted culture medium samples and EV samples of Norway spruce. A) Biological function, **B)** molecular function, **C)** cellular component of the proteins detected.
