# Supplementary material for: Extracellular vesicles of Norway spruce contain precursors and enzymes for lignin formation and salicylic acid
Source: Plant Physiol. 2024 May 21;196(2):788–809. doi: 10.1093/plphys/kiae287 (PMC11444294; doi:10.1093/plphys/kiae287)
Supplement: kiae287_Supplementary_Data [file pp.02044.2023-s06.pdf › pp.02044.2023-s06.pdf]

Plant Physiology®

Extracellular vesicles of Norway spruce contain precursors and enzymes for lignin formation, and salicylic acid

Santeri Kankaanpää, Enni Väisänen, Geert Goeminne, Rabah Soliymani, Sandrien Desmet, Anatoliy Samoylenko, Seppo Vainio, Gunnar Wingsle, Wout Boerjan, Ruben Vanholme, and Anna Karkonen

Review Timeline:

Submission Date:	20-Dec-2023
Editorial Decision:	07-Feb-2024
Revision Received:	09-Apr-2024
Accepted:	29-Apr-2024

07-Feb-2024

Dr. Anna Karkonen
Natural Resources Institute Finland
Helsinki 00790
Finland

RE: Extracellular vesicles of Norway spruce contain precursors and enzymes for lignin formation, and salicylic acid

Dear Dr. Karkonen:

We have received reviews of your manuscript, which you may view at the end of this letter. On the basis of the advice received and my own evaluation, I believe that, pending revision, this article may ultimately be acceptable for publication in Plant Physiology.
The reviewers were enthusiastic about the manuscript and its insights into the role of extracellular vesicles in lignin biosynthesis. The reviews clearly highlight areas for improvement.

When you are ready to submit your revision, click the below link and find the red arrow under your Author Tasks heading for this revision submission.

If you want to visit this version of the manuscript, you may click the link below and then click on the folder that reads "Post Decision Manuscripts".

Link Not Available

When uploading the revised version of this article, please be sure to include as complete an answer as possible to the referees' criticisms during the first round of reviews in the "response to reviewers" section.

Plant Physiology considers a revision for up to 60 days after a manuscript has been provisionally accepted; limited extension of this timeframe is at the discretion of the Monitoring Editor. A reminder notice will be sent to you 2 weeks before the deadline, at which time you may choose to contact the editorial office to request an extension; if so, please provide a justification for your request. After the expiration of this deadline, the article would need to be submitted as a resubmission and would be handled as such.

Thank you very much for giving us an opportunity to review this work. I look forward to receiving the next version.

Sincerely,

Editor
Plant Physiology

Want to add this revision deadline to your calendar? Click below!

-- Reviewer comments:
Reviewer #1 (Comments for the Author):

The research field of extracellular vesicles (EV) in plants has been neglected, or dominated by weak evidence and overblown claims (with the exception of the recent DeBellis et al. 2022 Nature Comm). The present study is the first to test if EV could be involved in lignification. Most lignifying systems, e.g. forest trees, are difficult to study as the lignification is accompanied by programmed cell death in the secondary xylem, so any EVs could be attributed to the cell death. Dr. Happonen (formerly Karokenen)'s research group has made advances using a lignifying spruce cell culture system, which has allowed the science to advance and informs directed strategies for the more time-consuming and difficult experiments in trees. Since the cultures are

not undergoing cell death, this system provides credible evidence that they have rigorously identified plant exovesicles that contain mono-, di- and oligolignols, salicylic acid, proteins, and other metabolites. As many other studies of plant EV have been based on poor electron microscopy and/or problematic biochemistry, I have been a skeptic on the topic, but between the DeBellis work on suberin and the present manuscript, I am becoming convinced that EVs are active in cells with complex phenolic metabolism.

The authors isolate EVs by centrifugation, and characterize their physical dimensions with transmission electron microscopy and nanoparticle tracking analysis (NTA). For subsequent analyses, they compare the EV's in lignifying conditions with the EV's where lignification is blocked with KI. The EV's were concentrated by centrifugation and the supernatant was used as 'media' controls. PCA of metabolomics shows EV samples are clearly different from the media. Whereas the media samples are cleanly split into lignifying and non-lignifying conditions, the EV samples are more variable. This is unpacked in the thorough analysis in Figures 3 and 4, as well as in strong supporting MS in Supplemental Figure S3, where we see that EV samples contain coniferyl alcohol, coniferaldehyde, ferulic and vanillic acids, as well as distinct patterns of dimer- and oligomer-glucosides. The metabolomics data presented in the heat map strongly support a biological role for EVs, as phenolic glucosides in EVs exhibit striking differences in lignin forming and KI-inhibited samples, as well as EV-depleted culture medium. The proteomics is carefully conducted and reveals some oxidative enzymes that could be important, however, the low abundance of some of the proteins ultimately makes reporting and interpretation of their potential roles less compelling.

The manuscript cites all the appropriate literature and is clearly written. Overall, it is an intriguing and high-quality study, even as it makes it clear that we have a long way to go to understanding the putative roles of EV in lignification.

Major concerns.
1. For proteomics, consider putting some cut-offs for protein abundance. The current Figure 6 is not very informative, it could go to supplemental. Instead make a table in the main text derived from Supplemental Table 3B for the proteins that are reliably detected in EV, but not in media. If a protein is not detected at all in one treatment or only one of three replicate samples, is it a meaningful result? For example, for the glutathione S-transferases, ABCB, MFS and MATE transporters, there were zero counts reported in almost all of the lignifying samples and only very low counts (0 or 1) in the non-lignifying samples. Given this, the discussion of glutathione transferase and transporters should be cut down. For proteomics of non-EDTA washed samples, only beta-xylosidase had consistently >1 counts, and it is also in the media. If these spectral counts are found in levels similar to histones, are they biologically important?
2. Expand the discussion of what was not found, e.g. no Exo70E homologs. This could be discussed in the context of the DeBellis work, as well. It is also worthwhile to note that no ESCRT components are detected. In DeBellis et al., they speculate either the PM evagination that is shown here in 7D or an ER-derived mechanism...we just don't know! But given no Exo70E homologs and no ESCRT components, do we need Figure 7B or C? If you say that these are the most well-verified mechanisms in mammalian cells and yeast, then ok, but from the plant data??
3. Why is Figure 3 in signal intensity and not quantified?
4. The authors say "When the next version (ver. 2) of the spruce genome will be published with improved gene models, it will be possible to evaluate the presence of signal peptides in the sequences of EV proteins in order to investigate the proportion of proteins that lack the signal peptide." Thinking of only the most highly abundant proteins detected (Table. S3B), consider manually searching for signal peptides for the top hits. What about Jokipii-Lukkari et al. 2018 RNA-seq data? Doesn't their supplemental data include the 5' sequences of the coding regions that would provide this information?

Minor points.
1. The pectin surrounding the EV is interesting and I agree with the authors interpretation, in contrast to the Prado et al. interpretation. One suggestion here is to add a scale bar to supplemental S2.
2. For supplemental Figure S4, add EV samples and EV-depleted labels similar to Figure 4.
3. For Figure 6A and B, make the EV-depleted culture medium labels consistent, e.g. Concentrated EV-depleted culture medium and Unconcentrated.

Reviewer #2 (Comments for the Author):

The study addresses an important question in lignin biosynthesis, providing strong evidence for the transport of lignin precursors and salicylic acid via extracellular vesicles (EVs). The research is interesting and solid, with robust cell culture, metabolomics, and proteomics experiments.

I have just a few suggestions to further improve the manuscript:

-The introduction provides a good overview of the topic, but it could explicitly state the research questions or hypotheses that the study aims to address.

-When I first looked at the metabolomics data, I wondered why you didn't find sinapyl alcohol or syringyl-rich oligolignols in the EVs. I was assuming that most of the extracellular lignin in cell cultures was made up of S-units. Later, I realized that spruce lignin consists mainly of G-lignin (and possibly H-lignin in cell cultures). So, it might be valuable to include information about the

types of monolignols present in the lignin from the cell cultures, maybe with a picture/micrograph showing it stained with phloroglucinol. Additionally, the authors could discuss whether different monolignols might use different transport mechanisms.

-Results show very low levels of metabolites in the EVs compared to the media. It would be constructive to quantify this difference and discuss whether this suggests that most lignin precursors transport might occur via passive diffusion (Models A and D, in Figure 7?). Could you discuss which model makes more sense based on your data/experience?

-The observation of pinoresinol glucoside levels in the EVs in the lignin-forming cultures is intriguing, especially when compared with the KI treatment at day 14. Please clarify the intensity levels of pinoresinol glucoside compared to the metabolites reported in Figure 4A.

-Isolation of EVs (methods): Do we know the molecular function of the EVs in cell cultures (intercellular communication or stress response)? I am curious why the authors did not attempt to isolate EVs from plant stem tissues. It would be useful to include a discussion on the feasibility of doing this. These insights would be valuable for fellow researchers in the field.

-GC-MS analyses (methods): Please elaborate on the steps used for deconvolutions in Analyzer Pro XD, especially considering the challenges of using NIST in identifying phenolic compounds after derivatization.

Overall, I enjoyed reading the manuscript. The models presented in Figure 7 are similar to those from early studies by Helen A. Stafford. The research is promising, I believe you are in the right path regarding the monolignol transport mechanisms. The key question is to clarify the extent of transport via EVs compared to passive diffusion or active transport. Considering the low amounts of metabolites found in the EVs, one could hypothesize that passive transport may be the primary mechanism for metabolites, while vesicle transport could be the primary route for laccasses and peroxidases. Any additional clarification on this point would be helpful.

RE: Extracellular vesicles of Norway spruce contain precursors and enzymes for lignin formation, and salicylic acid

Dear Dr. :

We have received reviews of your manuscript, which you may view at the end of this letter. On the basis of the advice received and my own evaluation, I believe that, pending revision, this article may ultimately be acceptable for publication in Plant Physiology.
The reviewers were enthusiastic about the manuscript and its insights into the role of extracellular vesicles in lignin biosynthesis. The reviews clearly highlight areas for improvement.

When you are ready to submit your revision, click the below link and find the red arrow under your Author Tasks heading for this revision submission.

If you want to visit this version of the manuscript, you may click the link below and then click on the folder that reads "Post Decision Manuscripts".

https://pphys.msubmit.net/cgi-bin/main.plex?el=A7It2Nuv3A5FDj2F6A9ftdrModEuNvL5cXSxrJlNdAZ

When uploading the revised version of this article, please be sure to include as complete an answer as possible to the referees' criticisms during the first round of reviews in the "response to reviewers" section.

Plant Physiology considers a revision for up to 60 days after a manuscript has been provisionally accepted; limited extension of this timeframe is at the discretion of the Monitoring Editor. A reminder notice will be sent to you 2 weeks before the deadline, at which time you may choose to contact the editorial office to request an extension; if so, please provide a justification for your request. After the expiration of this deadline, the article would need to be submitted as a resubmission and would be handled as such.

Thank you very much for giving us an opportunity to review this work. I look forward to receiving the next version.

Sincerely,

Sibongile Mafu
Monitoring Editor
Plant Physiology

-- Reviewer comments:

Reviewer #1 (Comments for the Author):

The research field of extracellular vesicles (EV) in plants has been neglected, or dominated by weak evidence and overblown claims (with the exception of the recent DeBellis et al. 2022 Nature Comm). The present study is the first to test if EV could be involved in lignification. Most lignifying systems, e.g. forest trees, are difficult to study as the lignification is accompanied by programmed cell death in the secondary xylem, so any EVs could be attributed to the cell death. Dr. Happonen (formerly Karokenen)'s research group has made advances using a lignifying spruce cell culture system, which has allowed the science to advance and informs directed strategies for the more time-consuming and difficult experiments in trees. Since the cultures are not undergoing cell death, this system provides credible evidence that they have rigorously identified plant exovesicles that contain mono-, di- and oligolignols, salicylic acid, proteins, and other metabolites. As many other studies of plant EV have been based on poor electron microscopy and/or problematic biochemistry, I have been a skeptic on the topic, but between the DeBellis work on suberin and the present manuscript, I am becoming convinced that EVs are active in cells with complex phenolic metabolism.

The authors isolate EVs by centrifugation, and characterize their physical dimensions with transmission electron microscopy and nanoparticle tracking analysis (NTA). For subsequent analyses, they compare the EV's in lignifying conditions with the EV's where lignification is blocked with KI. The EV's were concentrated by centrifugation and the supernatant was used as 'media' controls. PCA of metabolomics shows EV samples are clearly different from the media. Whereas the media samples are cleanly split into lignifying and non-lignifying conditions, the EV samples are more variable. This is unpacked in the thorough analysis in Figures 3 and 4, as well as in strong supporting MS in Supplemental Figure S3, where we see that EV samples contain coniferyl alcohol, coniferaldehyde, ferulic and vanillic acids, as well as distinct patterns of dimer- and oligomer-glucosides. The metabolomics data presented in the heat map strongly support a biological role for EVs, as phenolic glucosides in EVs exhibit striking differences in lignin forming and KI-inhibited samples, as well as EV-depleted culture medium. The proteomics is carefully conducted and reveals some oxidative enzymes that could be important, however, the low abundance of some of the proteins ultimately makes reporting and interpretation of their potential roles less compelling.

The manuscript cites all the appropriate literature and is clearly written. Overall, it is an intriguing and high-quality study, even as it makes it clear that we have a long way to go to understanding the putative roles of EV in lignification.

Response: We thank the Reviewer #1 for the nice comments on our ms.

Major concerns.
1. For proteomics, consider putting some cut-offs for protein abundance. The current Figure 6 is not very informative, it could go to supplemental. Instead make a table in the main text derived from Supplemental Table 3B for the proteins that are reliably detected in EV, but not in media. If a protein is not detected at all in one treatment or only one of three replicate samples, is it a meaningful result? For example, for the glutathione S-transferases, ABCB, MFS and MATE transporters, there

were zero counts reported in almost all of the lignifying samples and only very low counts (0 or 1) in the non-lignifying samples. Given this, the discussion of glutathione transferase and transporters should be cut down. For proteomics of non-EDTA washed samples, only beta-xylosidase had consistently >1 counts, and it is also in the media. If these spectral counts are found in levels similar to histones, are they biologically important?

Response: We thank Reviewer #1 for this comment and understand the concern. Due to the large size and long repetitive sequences, the version 1.0 genome assembly of Norway spruce contains many mistakes (e.g., missing exons in coding regions) and partial gene sequences in addition to full-length correct gene models. The fact that many protein sequences are of partial length affects the number of unique peptides obtained in the proteomic analysis. It is more probable to get higher peptide counts for proteins whose gene models are full-length and correct than for those whose gene models are of partial length and/or contain mistakes, for example gaps, in the sequences. Please, see the alignment of spruce homologous sequences to that of Arabidopsis Exo70E2 in the end of this file (for review purposes only). Despite this shortage, the current genome assembly is useful for studies and enables us to get information of the proteins present in spruce EVs. The above-mentioned situation is one reason we are discussing the proteomic data of all samples as one group and GO analyses have been conducted using the whole data. Technical handling of the vesicle samples is not easy, and membrane proteins are known to be more difficult than the soluble ones. The technical reproducibility is very challenging to standardize.

We have now included the Percolator node in the Proteome Discover (PD) analysis for the proteomic data (EDTA-extracted EV samples and corresponding EV-depleted culture media samples) and remade the Supplemental Table 3B and 3D with a lot of useful statistics (q value, false discovery rate (FDR), abundancies). Three runs of short protein lists (the unconcentrated EV-depleted culture medium samples) did not pass the Percolator analysis. For these, we used the Fixed Value Validator node. The novel information is helpful in analyzing the validity of the annotations. All the protein annotations now listed have a q value < 0.05. For many proteins (e.g., tau class glutathione transferases), the number of unique peptides obtained from the Percolator analysis was higher than that obtained in the earlier analysis using a Fixed Value Validator node in PD. In addition, some new annotations appeared by including the Percolator node in the analysis. We have kept the criteria to have at least two unique peptides for a protein to be in the table. This is because we have MS/MS fragmentation data for each unique peptide; these data can be added as Supplemental data if needed. For example, an annotation for a membrane-protein syntaxin was obtained in a single sample with two unique peptides. Based on the MS/MS profile, one of the unique peptides has 11 consecutive fragments. This means that the identification is clear even though this membrane protein was now detected only in one sample — although it is most likely present in all EV samples and was seen in a trial run also for the H_2O-treated control sample. Based on the stringent statistical as well as the fragmentation information, we consider that the reported list is reliable. We have also prepared a summary table (Table 1) in the main text to summarize the most interesting proteins in relation to the focus of this work.

According to the comment of Reviewer #1, we have transferred the remade Figs 6A and B to Supplemental Figures (now Supplemental Fig. S6) and shortened the discussion about ABC transporters and glutathione transferases.

2. Expand the discussion of what was not found, e.g. no Exo70E homologs. This could be discussed in the context of the DeBellis work, as well. It is also worthwhile to note that no ESCRT components are detected. In DeBellis et al., they speculate either the PM evagination that is shown here in 7D or an ER-derived mechanism...we just don't know! But given no Exo70E homologs and no ESCRT components, do we need Figure 7B or C? If you say that these are the most well-verified mechanisms in mammalian cells and yeast, then ok, but from the plant data??

Response: We thank Reviewer #1 for the comment. We have now extended the discussion on the topic. As seen in the separate alignment of the spruce homologous sequences present in the ConGenIE database with the Arabidopsis Exo70E2 and Exo70A2 (see alignment in the end of this file), none of the spruce gene models is full length. Additionally, there are also very short gene models that have homology in the conserved areas but lack the rest of the sequence. Additionally, the spruce EV-proteomic data contains an annotation for a protein (MA_96110g0010) associated with the ESCRT III complex assembly. This is now mentioned in the discussion of the ms. At the present stage, we think that we cannot abandon EXPOs' or ESCRT' role in spruce EV generation. The version 2 of the genome assembly that the Swedish scientists are actively working on (pers. comm. Prof. Nathaniel Street) will be very useful for clarifying this question.

3. Why is Figure 3 in signal intensity and not quantified?

Response: None of the metabolites have been quantified, as the process would involve running calibration curves for each metabolite and/or spiking with standard compounds. For untargeted explorative research, this is practically impossible. In such studies, the peak intensity is used as a surrogate for the concentration. It is important to note that the response factor (i.e., the signal obtained for a given concentration) depends on the physico-chemical properties of the compound. Consequently, comparing peak intensities is only valid within the same compound across different samples, not between different compounds. Essentially, the higher signal intensity signifies a greater abundance of the metabolite in the respective sample.

4. The authors say "When the next version (ver. 2) of the spruce genome will be published with improved gene models, it will be possible to evaluate the presence of signal peptides in the sequences of EV proteins in order to investigate the proportion of proteins that lack the signal peptide." Thinking of only the most highly abundant proteins detected (Table. S3B), consider manually searching for signal peptides for the top hits. What about Jokipii-Lukkari et al. 2018 RNA-seq data? Doesn't their supplemental data include the 5' sequences of the coding regions that would provide this information?

Response: It is the Norway spruce ver. 1 genome assembly that Jokipii-Lukkari and colleagues have also utilized in their work (Jokipii-Lukkari et al., 2018). Hence, their expression data are based on the same gene models that we use here. When making phylogenetic trees of the genes of interest, Jokipii-Lukkari et al. notify "any aberrant sequences (e.g., truncated or those missing conserved

amino acids… were manually identified and subsequently discarded from further analyses“. Furthermore, Jokipii-Lukkari et al. have verified one coding sequence (of *PaMC1*) by cloning. As compared to eudicot species such as Arabidopsis, using the Norway spruce genome sequence presents challenges. To study any gene or protein of interest, cDNAs must be verified by cloning. We have experience that this is not always easy, since designing RACE primers to obtain the 5’ or 3’ part of the coding region is not always successful. We think that in the context of the present manuscript, it is not feasible to resolve the beginning of the coding sequences of all genes of interest. After the publication of the version 2 genome assembly that the Swedish scientists are actively working on (pers. comm. N. Street, UPSC) this will be feasible.

Minor points.

1. The pectin surrounding the EV is interesting and I agree with the authors interpretation, in contrast to the Prado et al. interpretation. One suggestion here is to add a scale bar to supplemental S2.

Response: We are glad to hear that Reviewer #1 agrees with our interpretation for pectin. However, we think that addition of the scalebar to Fig. S2 is unnecessary.

2. For supplemental Figure S4, add EV samples and EV-depleted labels similar to Figure 4.

Response: We cannot add the same headers in Figure S4 as those in Figure 4, because the two figures show different things. For each cell in Figure S4, the comparison is made between the EV samples and the corresponding media, as explained in the legend. Given this question by Reviewer #1, we re-evaluated which header could be used. Unfortunately, we could not come up with a title that covers the content, unless it would be a copy of the title of the figure itself ("Evaluation of the presence of residual medium [...]", which is not desirable.

3. For Figure 6A and B, make the EV-depleted culture medium labels consistent, e.g. Concentrated EV-depleted culture medium and Unconcentrated.

Response: This has been corrected.

Reviewer #2 (Comments for the Author):

The study addresses an important question in lignin biosynthesis, providing strong evidence for the transport of lignin precursors and salicylic acid via extracellular vesicles (EVs). The research is interesting and solid, with robust cell culture, metabolomics, and proteomics experiments.

I have just a few suggestions to further improve the manuscript:

-The introduction provides a good overview of the topic, but it could explicitly state the research questions or hypotheses that the study aims to address.

Response: We thank Reviewer #2 for the nice comments on our ms. Now the aims of our study have been added to the Introduction.

-When I first looked at the metabolomics data, I wondered why you didn't find sinapyl alcohol or syringyl-rich oligolignols in the EVs. I was assuming that most of the extracellular lignin in cell cultures was made up of S-units. Later, I realized that spruce lignin consists mainly of G-lignin (and possibly H-lignin in cell cultures). So, it might be valuable to include information about the types of monolignols present in the lignin from the cell cultures, maybe with a picture/micrograph showing it stained with phloroglucinol. Additionally, the authors could discuss whether different monolignols might use different transport mechanisms.

Response: We have now re-written the introduction to avoid confusion and write only about units that occur in the lignin of a coniferous species. A sentence with two citations has also been added to inform that the extracellular lignin formed in the culture medium of cultured spruce cells is composed of subunits G and H as observed by using NMR methods. Considering different transport mechanisms of different monolignols, the discussion would be totally hypothetical, so we have not added that. All of the monolignol alcohols are able to pass the membrane via diffusion. There is also a report about a transporter that can transport *p*-coumaryl alcohol. The EV-mediated transport is one option. These possible ways of transport lignin precursors are already discussed in the present ms.

-Results show very low levels of metabolites in the EVs compared to the media. It would be constructive to quantify this difference and discuss whether this suggests that most lignin precursors transport might occur via passive diffusion (Models A and D, in Figure 7?). Could you discuss which model makes more sense based on your data/experience?

Response: Indeed, as explained in the manuscript, the EVs showed relatively low levels of metabolites as compared to the medium. In the text we wrote: "Notably, the medium samples were much more concentrated than the corresponding EV samples, and this complicated the comparative analyses." This observation hints that precursor transport thus quantitively goes via passive diffusion (and/or with active transport) and not via EV-mediated transport. However, without insight in the lifetime of EVs this remains hypothetical. The alternative hypothesis is that EVs do play a major role, but that they quickly release their cargo (and disintegrate). The low signal intensity is, in the first place, intrinsic to the low abundance of EVs. To address the reviewers' comment, we rephrased the claim to: "Because of the relatively low abundance of EVs in the culture, the EV samples were much less concentrated than the corresponding medium samples. This difference in concentration complicated the comparative analyses."

-The observation of pinoresinol glucoside levels in the EVs in the lignin-forming cultures is intriguing, especially when compared with the KI treatment at day 14. Please clarify the intensity levels of pinoresinol glucoside compared to the metabolites reported in Figure 4A.

Response: Thanks for this comment. We previously showed that the KI treatment slows down lignin coupling reactions in spruce cell cultures, not the biosynthesis of the precursors (Laitinen et al., 2017). The abundance of a dimer should be interpreted with care: at each given timepoint the

abundance is a combination of the coupling of two monomers minus the further coupling of the dimer to higher order oligomers. The accumulation of dimers might thus be the result of a hindered coupling to higher oligomers caused by KI. Specifically, for pinoresinol hexoside, a hexosylation took place after the dimerization. Hexosylation reaction happens intracellularly indicating that the intracellular concentration of pinoresinol increased and was then rather glycosylated than further coupled to another lignin monomer.

-Isolation of EVs (methods): Do we know the molecular function of the EVs in cell cultures (intercellular communication or stress response)? I am curious why the authors did not attempt to isolate EVs from plant stem tissues. It would be useful to include a discussion on the feasibility of doing this. These insights would be valuable for fellow researchers in the field.

Response:
We do not know the exact molecular function of EVs in plant cell cultures. Cell wall modification is needed in all plant cells during active growth. Woith and collaborators (2021) discuss that EV-located cell-wall-polysaccharide-modifying enzymes could be a means to facilitate EVs´ penetration through the cell wall. In EVs isolated from growing sunflower seedlings, polysaccharide-modifying enzymes are also observed (Regente et al. 2017). In the lignin-forming cell culture of Norway spruce, the EVs were observed to contain polysaccharide-modifying enzymes, but also oxidative enzymes (laccases, peroxidases) and dirigent proteins possibly involved in lignin biosynthesis. Hence, one function of EVs in the cell cultures seems to be the transport of cargo into the apoplast in addition to the conventional protein transport. This is discussed in the manuscript.

Now that we have accumulated data from the cell cultures, the logical next step is to study EVs from developing xylem. Developing xylem of Norway spruce is excised from the outer surface of a tree trunk after the bark is peeled off (figure in Väisänen et al. 2018). There are several wounded cells on both sides of the developing xylem. For this reason, isolating pure apoplastic fluid with infiltration-centrifugation method, commonly used to isolate apoplastic fluid in plants, is not possible, since significant contamination from the cytosolic content would occur. Use of antibodies may potentiate affinity isolation of EVs. This information is now added to the manusript.

-GC-MS analyses (methods): Please elaborate on the steps used for deconvolutions in Analyzer Pro XD, especially considering the challenges of using NIST in identifying phenolic compounds after derivatization.

Response: This information has now been added to the methods section.

Overall, I enjoyed reading the manuscript. The models presented in Figure 7 are similar to those from early studies by Helen A. Stafford. The research is promising, I believe you are in the right path regarding the monolignol transport mechanisms. The key question is to clarify the extent of transport via EVs compared to passive diffusion or active transport. Considering the low amounts of metabolites found in the EVs, one could hypothesize that passive transport may be the primary

mechanism for metabolites, while vesicle transport could be the primary route for laccasses and peroxidases. Any additional clarification on this point would be helpful.

Response: We are glad to hear that you have liked our manuscript and that you think that we are on a right track. Thank you also for mentioning Prof. H.A. Stafford's work and her model. We were not aware about that, and a citation to one of her papers has now been added. Some additional discussion on the roles of EVs has also been added into the discussion. The observation that there was a relatively low abundance of EVs in the culture medium hints that lignin precursor transport thus quantitively goes via passive diffusion (and/or with active transport) and not via EV-mediated transport. However, without insight in the lifetime of EVs this remains hypothetical. The alternative hypothesis is that EVs do play a major role, but that they quickly release their cargo (and disintegrate). Protein transport, however, will go via the conventional protein secretion pathway shown in Fig. 7A or via the unconventional protein secretion pathway such as the EV-mediated transport.

Anna Happonen, 03/24 (For review purposes only)

Exo70A1/Exo70E2 sequence homologs in Norway spruce

Let´s have a look of homologs of AtExo70E2 and AtExo70A1 in Norway spruce genome.

>NP_200909.1 exocyst subunit exo70 family protein E2 [Arabidopsis thaliana]

>AtExo70E2

MAEFDSKVPVSGMNNHVFEACHHVVKALRASDNNLDANLRKLLSDLEMHLSTFGIADTKVEDAGFSEI
KKRFKEAVKRIRSWETNQSTMFEAGLSEADQFFQALYDVQTVLVGFKALPMKTNQMEKDVYNQATVAL
DIAMLRLEKELCDVLHQHKRHVQPDYLAVSSRRKDIVYDESFVSLDDEVIVEASSHEDDEQISDFYNSDL
VDPIVLPHIKAIANAMFACEYDQPFCEAFIGVQREALEEYMVTLEMERFSCVDVLRMDWEDLNGAMRK
WTKVVKIITQVYLASEKQLCDQILGDFESISTACFIEISKDAILSLLNFGEAVVLRSCKPEMLERFLSMYEVSA
EILVDVDNLFPDETGSSLRIAFHNLSKKLADHTTTTFLKFKDAIASDESTRPFHGGGIHHLTRYVMNYLKLL
PEYTDSLNSLLQNIHVDDSIPEKTGEDVLPSTFSPMARHLRSIVTTLESSLERKAQLYADEALKSIFLMNNF
RYMVQKVKGSELRRLFGDEWIRKHIASYQCNVTNYERSTWSSILALLRDNNDSVRTLRERCRLFSLAFD
DVYKNQTRWSVPDSELRDDLHISTSVKVVQSYRGFLGRNAVRIGEKHIRYTCEDIENMLLDLFECLPSPR
SLRSSRKR

>AtExo70A1

MAVDSRMDLLSERAVLMRASLQKSQTITDNVVSILGSFDSRLSALETAMRPTQIRTHAIRKAHENIDRTL
KAAEVILSQFDLLRQAETKVLKGPHEDLESYLDAIAQLRKIIRYFMSNKSFKSSDGVLNHANSLLAKAQSK
LEEEFKQLLASYRILRMTFYLNYRSCHLTKIFFCEIKHFKAVEPDRLFDGLPNSLRPSSDGDGGGKPHGGH
HNDDAETAAYTLPILIPSRVLPLLHDLAQQMVQAGHQQQLLQIYRDTRSFVLEESLKKLGVEKLSKEDVQ
RMQWEVLEAKIGNWIHFMRIAVKLLFAGERQVCDQIFRGFDSLSDQCFAEVTVSSVSMLLSFGDAIARS
KRSPEKLFVLLDMYEIMRELHTEIETIFKGKACLEIRDSATGLTKRLAQTAQETFGDFEEAVEKDATKTAVLD
GTVHPLTSYVINYVKFLFDYQTTLKQLFLEFGNGDDSNSQLASVTMRIMQALQNNLDGKSKQYKDPAL
THLFLMNNIHYMVRSVRRSEAKDLLGDDWVQRHRRIVQQHANQYKRVAWTKILQSSSAQGLTSSGG
GSLEGGNSSGVSRGLLKERFKMFNMQFDELHQRQSQWTVPDTELRESLRLAVAEVLLPAYRSFLKRFGP
LVESGKNPQKYIKYTAEDLERLLGELFEGKSMNEPRR

There seems to be 17 members in this family in Norway spruce (obtained when ConGenIE database is blasted with the AtExo70E2):

MA_10428691g0010, MA_10431828g0010, MA_121699g0010, MA_12790g0040, MA_129851g0010, MA_130238g0010, MA_138348g0010, MA_22002g0010, MA_25591g0010, MA_29736g0010, MA_33877g0010, MA_418978g0010, MA_494970g0010, MA_5009135g0010, MA_8703090g0010, MA_89549g0010, MA_9976656g0010

Sequences in the end of the file.

Let's align them with the Arabidopsis ones. Let's do alignment so that the longer ones are aligned separately to the short ones. This gives better idea of the alignment.

Longer sequences of spruce aligned with the Arabidopsis ones:

```
CLUSTAL O(1.2.4) multiple sequence alignment


AtExo70E2           ------------------------------------------------------MAEFDS 6
AtExo70A1           ------------------------------------------------------------ 0
MA_494970g0010      ------------------------------------------------------------ 0
MA_12790g0040       ------------------------------------------------------------ 0
MA_22002g0010       ------------------------------------------------------------ 0
MA_29736g0010       ------------------------------------------------------------ 0
MA_89549g0010       ------------------------------------------------------------ 0
MA_25591g0010       ---------------------------------------MLRMYALIQGTFSEGIKEFTN 21
MA_138348g0010      ------------------------------------------------------------ 0
MA_121699g0010      ------------------------------------------------------------ 0
MA_5009135g0010     ------------------------------------------------------------ 0
MA_418978g0010      ------------------------------------------------------------ 0
MA_130238g0010      MDQPGDEKKMAEAHSRNNSVVNSECPSEDQGSLDENSECLSQSFVVLDENLD-------- 52
MA_33877g0010       ------------------------------------------------------------ 0


AtExo70E2           ---KVP----------VSGMNNHVFEACHHVVKALRASDNNLDANLRKLLSDLEMHLSTF 53
AtExo70A1           ----MAVDS----------RMDLLSERAVLMRASLQK-SQTITDNVVSILGSFDSRLSAL 45
MA_494970g0010      ---------------------------------------------MLTVPSTFDSHTSAR 15
MA_12790g0040       ------------------------------------------------------------ 0
MA_22002g0010       ------------------------------------------------------------ 0
MA_29736g0010       ---------------------------------------------MLTILT-FDNCLSPT 14
MA_89549g0010       --------ME---IQEEAAGEDGVVATTHHIDKSLGS-SEDMTRDMLTTLT-FDNGLSAT 47
MA_25591g0010       GGFTMPIGWKIEKATADQGGAKEKSSGHGTSYCEI-----------SRHLREHDERHAHS 70
MA_138348g0010      --------MEIEEVAAHQGGEDRVVAMVHHIVTSLST-SENMTNDMLTMLSTFDNRPSTM 51
MA_121699g0010      ------------------------------------------------------------ 0
MA_5009135g0010     ------------------------------------------------------------ 0
MA_418978g0010      ------------------------MASPEEDDTSESV-VETLADDMHRIFSKLDGPFSCI 35
MA_130238g0010      NAEKLILKWDMASSEAAKQS-MIFEGQPEEAEKYLKT-VDE----LQKLMESVSLSQADV 106
MA_33877g0010       ------------------------------------------------------------ 0


AtExo70E2           GIADTK------VEDAGFSEIKKRFKEAVKRIRSWETNQS-------------------- 87
AtExo70A1           ETAMRPTQIRTHAIRKAHENIDRTLKAAEVILSQFDLLRQA------------------- 86
MA_494970g0010      SHQNPG-N-----PPGKSADFETNLAFADRIISFWDSHSSE------------------- 50
MA_12790g0040       ------------------------------------------------------------ 0
MA_22002g0010       ------------------------------------------------------------ 0
MA_29736g0010       SHQNPGK------LHGKSADLEANLAFAERIISSSDSNSFN------------------- 49
MA_89549g0010       SHQNPERN-----PHEKSTDLEANLASADRIISLWDSKSFN------------------- 83
MA_25591g0010       PL-HL-RQ-----PPFRHE-------TSKSLQPTWKIGRFGYQFCLRRPKNLVLGLE-PV 115
MA_138348g0010      SHQCP-TN-----PPRKSADLESNLASADRIISFWESNSAK------------------- 86
MA_121699g0010      ------------------------------------------------------------ 0
MA_5009135g0010     ------------------------------------------------------------ 0
MA_418978g0010      DGANNRKN----EEFVLPVNLEEELSSLEDKIMKWEGPGFMV-----------AG----- 75
MA_130238g0010      H---KAQN----VLQIAMVRLEEEFRNLLLVHSEPLDPELVL-----------NPASKAS 148
MA_33877g0010       ------------------------------------------------------------ 0


AtExo70E2           -----TMFEAGLSEADQFFQALYDVQTVLVGFKALPMKTNQMEKDVYNQATVALDIAMLR 142
AtExo70A1           ---ETKVLKGPHEDLESYLDAIAQLRKIIRYFMSNKSFKS--SDGVLNHANSLLAKAQSK 141
MA_494970g0010      ----CMLFQSSRKETNEYLAAVDGLRDLMEILVVTQSRSA-----DLVRAHRLMEMSMAC 101
MA_12790g0040       ------------------------------------------------------------ 0
MA_22002g0010       ------------------------------------------------------------ 0
MA_29736g0010       ----SILFQSNPKETNEYLDAVDGLRHVMESLVVTHSSSD-----KMVRAQRLMEMSMAR 100
MA_89549g0010       ----SMLFQSNPKEASEYLDAVDGLRHLMETLVVVHSSSA-----MLVRAQRLMEMSMVR 134
MA_25591g0010       QTLDPVPEQSKRSQ--RVSCLLGWSPSPLENLVVTHNSSA-----KLVRAQRLMEVWMAR 168
MA_138348g0010      ----AMLFQSSRKEANEYLTAVDGLRHLMENMVLVHSSSA-----KMVRAQRLMEMSMAR 137
MA_121699g0010      -----MLFQSSRKEVYEYLSAVDSLRQLMENLVVTPSSSA-----KLVRAQRLMEMSMPR 50
MA_5009135g0010     ------------------------------------------------------------ 0
MA_418978g0010      ---EGSNSSEETERVYSYLRAVDDVQSLVNGFSQLPDDVK--DFTVLQRIEAIFHQAMDC 130
MA_130238g0010      FRSLNESFGGQPEEAEKYLKTVDELQKLMESVSLSQADV--------HKAQNVLQIAMVR 200
MA_33877g0010       ------------------------------------------------------------ 0


AtExo70E2           LEKELCDVLHQ--------------------------HKRHVQPDYLAVSSRRKDIVYDE 176
AtExo70A1           LEEEFKQLLASYRILRMTFYLNYRSCHLTKIFFCEIKHFKAVEPDRLFDGLP-NS----- 195
MA_494970g0010      LQKEFHRILTV--------------------------NVEGTM-----APADANS----- 125
MA_12790g0040       ------------------------------------------------------------ 0
MA_22002g0010       ------------------------------------------------------------ 0
MA_29736g0010       LQKEFHRILSA--------------------------NVEPTM-----APVDGNS----- 124
MA_89549g0010       LEKEFHRILSA--------------------------NVEPSM-----APDDGNS----- 158
```

```
MA_25591g0010       LKIEFQQILSA--------------------------NVEPTM-----VPDDSDS----- 192
MA_138348g0010      LEKEFHRILSA--------------------------NVKPTI-----APDDSNS----- 161
MA_121699g0010      LQKEFQRILSA--------------------------NVEPTL-----APDDGNL----- 74
MA_5009135g0010     ------------------------------------------------------------ 0
MA_418978g0010      LEEEFRALLEK--------------------------HSNAVDPDCLFDAASGPS----- 159
MA_130238g0010      LEEEFRNLLLV--------------------------HSEPLDPELVLNPASKAS----- 229
MA_33877g0010       ------------------------------------------------------------ 0


AtExo70E2           SFVSLDDEVIVEA--SSHEDDE-----------QISDFYNSDLVDPIVLPHIKAIANAMF 223
AtExo70A1           LRPSSDGDGGGKPHGGHHNDDAET-----------AAYTLPILIPSRVLPLLHDLAQQMV 244
MA_494970g0010      SHESTDEEGSRGASCRSRSSNADKISSCRSRSSNADKICEIDLMPLDAVLDLRNIAKRMV 185
MA_12790g0040       ------------------------------------------------------------ 0
MA_22002g0010       ------------------------------------------------------------ 0
MA_29736g0010       REASSDD--------GSTDEDESSSGRRQSWNCNPDEICKIDMMCVEAVLDLRSIAKRMV 176
MA_89549g0010       SHASSHE--------GLIDEDERSSGHRQIWNSKADEICEIDMMSVESVLDLRSIAKRMV 210
MA_25591g0010       SHASSDD--------ESTDEEGSSGGHRRSRNSNADEICEIDMMSLEAVLDLRSIAKRMV 244
MA_138348g0010      NHGSSDD--------GSTDEDVSSSGHRRSRNSNAEEICEIDMMPLDAVLDLRSIAKRMV 213
MA_121699g0010      SRGSSDDDRGSSDD-ESTDQVGNSGGRRRTRNCNADEICEIDMMPLDAVLDLRSIAKRIV 133
MA_5009135g0010     ------------------------------------------------------------ 0
MA_418978g0010      F-NSSCD---ERAFRNL-ADDEKEEEDVPVAQPVGDLNFTIDSLPSEIVNRLSEIAVRMV 214
MA_130238g0010      F-RSLNESFGDRPSHSSDDDEAEEEEEEAVSHPLADKICEMDLIPQDVIPDLHRIAERMV 288
MA_33877g0010       ------------------------------------------------------------ 0


AtExo70E2           ACEYDQPFCEAFIGVQREALEEYMVTLEME--RFSCVDVLRMDWEDLNGAMRKWTKVVKI 281
AtExo70A1           QAGHQQQLLQIYRDTRSFVLEESLKKLGVE--KLSKEDVQRMQWEVLEAKIGNWIHFMRI 302
MA_494970g0010      AGGYWRDCVRIYGLTRKSVMEGCLNRIGVG--RLSATDVKKMEWGILDMKIKNWIGAVSI 243
MA_12790g0040       ------------------------------------------------------------ 0
MA_22002g0010       ------------------------------------------------------------ 0
MA_29736g0010       AGGYGSECVRIYGLTRKSVIEGCLNRIGVE--RLSTSDVQKMEWGILDIKIKKWIRAVRI 234
MA_89549g0010       AGGYGSECVRIYGLTRKSYEQRPEDRMGNSRHWLSTSNVQKIEWGILDINIQKWIRVVRI 270
MA_25591g0010       AAGHGRECFRIYEKTRKSVIEGCLNRIGVD--RLSTSDVQKMEWDILDIKIKKWIRAVRI 302
MA_138348g0010      SGGYGSECVRIYGQTRKSVIKECLNRIGVE--RLSTSDVQKMEWEILDFKIKNWIRSVRI 271
MA_121699g0010      AGGYGSECIRIYGLTRKSVIKRCLNRIGVE--WLNTSDVQKMEWAVLDIKIKNWIRAARI 191
MA_5009135g0010     -------------------------------------------------KIKKWIRAVRI 11
MA_418978g0010      RAGYRTECCQVYCMIRKMFLEESVMRLGFE--KLSIEEVQRIQWELLEVEISKWMQTLKV 272
MA_130238g0010      SAGYTRECMQVYGSIRKSMVDESFLRLGVE--KLSIGDVQRLGWETLEVKIKKWIRAAKV 346
MA_33877g0010       ------------------------------------------------------------ 0


AtExo70E2           ITQVYLASEKQLCDQI----------------LGDFESISTACFIEISKDAILSLLNFGE 325
AtExo70A1           AVKLLFAGERQVCDQI----------------FRGFDSLSDQCFAEVTVSSVSMLLSFGD 346
MA_494970g0010      AVRILFASEKQLCDEI----------------FAGLNHVRDSCFAEVVKEPTTKLLDFGE 287
MA_12790g0040       ------------------------------------------------------------ 0
MA_22002g0010       ------------------------------------------------------------ 0
MA_29736g0010       AVRILFASEKR-------------EKRFCDEIFSGLHSVSDFCFAELVKGPTTKLLVFGE 281
MA_89549g0010       AVRILFASE----------------KRLCDKIFAGLNNVSEFCFAEVVKGPTMKLLAFGE 314
MA_25591g0010       VVRILFASEKLLCDEI----------------FAGINNVRDSCFTEVVKGPTTKLLAFGE 346
MA_138348g0010      GVRILMASEKRLCDEI----------------FSGLNNVRDSCFAEVVKGPTTKLLAFGE 315
MA_121699g0010      AVRILFASEKRLCDEIFAGLNHVSEKRLCDEIFAGLNHVRDSCFTEVVKGPTTKLLAFGE 251
MA_5009135g0010     VVRILFASEKRLCDEI----------------FAGIINVRDSCFTEVVKGPTTKLLAFGE 55
MA_418978g0010      SVNVFMSSERILCDRV----------------FVDLPSVSETCFGELARGVMIQFLNFGE 316
MA_130238g0010      CIRMLFASEKHLSEQV----------------FDGLDSVGETCFSELAKGATMQLFNFSE 390
MA_33877g0010       ------------------------------------------------------------ 0


AtExo70E2           AVVLRSCKPEMLERFLSMYEVSAEILVDVDNLFPDETGSSLRIAFHNLSKKLADHTTTTF 385
AtExo70A1           AIARSKRSPEKLFVLLDMYEIMRELHTEIETIFKGKACLEIRDSATGLTKRLAQTAQETF 406
MA_494970g0010      SVAMNQKGSERLFR---------------------EPCAGVRTQASRIFVRLGKSAIGIM 326
MA_12790g0040       ---MGKKSSDRLFGVLDMYDCLSKLMPDIDTVYSQKECVGVRTKASAVLVRLGEAARGIF 57
MA_22002g0010       -----------------MYETLSELMPDFDIVYCQKSCASVRTQVSTIVVQLGESALGIL 43
MA_29736g0010       SVALSKKSSERLFRVLDMYETLWDLIPDIDTVYSQESCASVRTQASTILVRLGESARGIL 341
MA_89549g0010       IVAMSKMSSERLFRVLDMYETLWDLMPNIDTVYCQESCASVKTQASTILVRLGESAWGIL 374
MA_25591g0010       SVAMSKTVSERLFRVLDMYEILSDLMPDLDIVYCQKSWASLRTHVSMNVVQLGESALGIL 406
MA_138348g0010      SVAMSKKSSERLFRVLDMYETLWDLMPDIDTVYCQDSCASVRTQASTIFVRLRDSALGIM 375
MA_121699g0010      SVAMSRKSSEKLFRVLDMYETLSDLMPDIDTVYCQESCASVRAQASTIFVRLGESAWGIL 311
MA_5009135g0010     SVAMSKKVSERLFRVLDMYETLSDLMPDFDIVYGQKSCASVRTQVSMNVVRLGESALGIL 115
MA_418978g0010      AVAISRRSPEKLFKILDMYETLWELIPKINSIFSGEVCSTLRSEASAVWLRLGEAARGTF 376
MA_130238g0010      AIAISRRSPEKLFKILDLYETLSELLPDIEIVFSNEMCANVKTQASEILTRLGEAARGIF 450
MA_33877g0010       -----------------MYESLSDLLPVIKSIVLGKSCTSIRAEADGILTRLGEAAKGTF 43
                                                       .    ::        :* . :   :

AtExo70E2           LKFKDAIAS--DEST-------RPFHGGGIHHLTRYVMNYLKLLPEYTDSLNSLLQNIHV 436
AtExo70A1           GDFEEAVEK--DATK-------TAVLDGTVHPLTSYVINYVKFLFDYQTTLKQLFLEFGN 457
MA_494970g0010      TVFETAIHK--ENSK-------TSVPGGTIHPLTR------------------------- 352
MA_12790g0040       IEFENAVHG--ENSK-------TRIPGGTIHPLTRYVMNYLSFMSDYKEALVKITAHVPM 108
```

```
MA_22002g0010             TEFENAVQV--ENSK-------TPVAGGTIHPLTRYVMNYLSFLSDYKEALMKIIANAPI 94
MA_29736g0010             REFENAILA--ENSK-------TPVPGGTIHPLTGYVMNYLIFLSDYKETLMNITANAPV 392
MA_89549g0010             TDFENAIQA--ENSK-------TPVPGGTIHPLTGYVTNYLIFLSDYKETLMNIMVNSQI 425
MA_25591g0010             RNSRMQSRWRIQIHRSPALRFIRLQGLATIHPLTRYVMNYLSFLSDYKEVLMNIIANAPI 466
MA_138348g0010            TTFENAIQA--ENSK-------TPVPGGTIHPLTGYVMNYLSFLSDYKETLMNMMASATI 426
MA_121699g0010            TEFENAIQA--ENSK-------TPVPGGTIHPLARYVMNYLSFLCDYKEALMNITANAPI 362
MA_5009135g0010           TEFENAVQV--ENSN-------TPVAGGTIHPLTRYVMNYLSFLSDYKEVLMNIIANAPI 166
MA_418978g0010            LELENAIKG--DLAK-------NPVPGGAIHPLTRYVMNYMRLACDYRDTLEQVFKEDGG 427
MA_130238g0010            TEFENAIQR--ETSR-------TPIPGGTIHPITRYVMNYISFISDYKETLMKLIVNKPS 501
MA_33877g0010             VEFENAIQR--ETSK-------TPIPGGAIHPLTRYVMNYIKFLFDYTDTLQQLIDEKKE 94
                             .       :               . :* ::

AtExo70E2                 DDS--IPE---------------KTGEDVLPSTFSPMARHLRSIVTTLESSLERKAQLYA 479
AtExo70A1                 G-----------------------------DDSNSQLASVTMRIMQALQNNLDGKSKQYK 488
MA_494970g0010            ----ALPDGLMGMSGDLDGHDS------ELGSPASALSVKLRWIIIILQCKLDLKSNLYK 402
MA_12790g0040             DIPRALPNGLMGLFEDQDWDVE------EQGRPASVLSVRLGWIIIILQCKLDTTSKLYK 162
MA_22002g0010             DIPKALPDGLMGLFGDLDGPAS-------------DLSVKLGCIIIILQCKLDLKSNVYE 141
MA_29736g0010             DISKDLPDTLMDLFGELDGHGD------DLGSVASAVSVRLGWIIISLQKGQRFEASR-- 444
MA_89549g0010             GISKDVPDDLVDLS----RKDD------ELGSLAFALSVRLGWIIISLQCKLHMKSNLYQ 475
MA_25591g0010             DIAKALREGVMGL----------------------------------------------- 479
MA_138348g0010            D-----------------------GQDDELDSRASALSTRLGWIIINLQCNLDLKSNLYK 463
MA_121699g0010            AIPKALPVGLVGSSGDLVGHDDELGSPDELGSPASVLRVRVGWIITNLQGKLELKSNLYK 422
MA_5009135g0010           DIAKALPEGVMGLSEDLERSDN------ELGSPASDLSVKLGRIIFILQCKLDLKSILYE 220
MA_418978g0010            AEMNGVSARALDHSDVLEFRNG------TNTTDSSPLSTKTIAIMELLESNLNAKSKLYK 481
MA_130238g0010            QTSNLLPEGLIDL--IGEYDRA------SDSDNQSVLSLHLIWITLILQNNLEGKSKVYK 553
MA_33877g0010             AIPRQLSDEFTEF---PEEDRH------GDRSNTTPLARRLLSITSLLESNLEGKSKLYK 145


AtExo70E2                 DEALKSIFLMNNFRYMVQKVKGSELRRLFGDEWIRKHIASYQCNVTNYERSTWSSILALL 539
AtExo70A1                 DPALTHLFLMNNIHYMVRSVRRSEAKDLLGDDWVQRHRRIVQQHANQYKRVAWTKILQSS 548
MA_494970g0010            DVALSNLFLMNNLHYIVKKVKGSKLLGLLGYGWLRKNQDNVRHYAANSERAAWMKALDSL 462
MA_12790g0040             DVALSYLFLMNNLNYIVKKIKDSELLGLLGYGWLTKNQGKVRQYAANYERGAWMKVLNCL 222
MA_22002g0010             DVSLSYLFLMNNFHYIVKKVKNSKLLGLLGYGWLRKNQDNVGHYAANYERAAWMKALNCL 201
MA_29736g0010             ----------AARVWLVEEESGQELLGLLGYGWLRKNQGRMRQYAENYEREAWMKALNCL 494
MA_89549g0010             DVSLSYLFLMNNLHYIVKKVKGSKLLGLFGYGWLRKNQCKVRKYAENYERAAWTKALDCL 535
MA_25591g0010             -------------------VKGSKLLELLGYRWLRENQDKVKHYAANYERVAWMKALNCL 520
MA_138348g0010            DVALSNLFLMNNLHYIVNKVKGSKLLGLLGYGWLRQNQSEVREYAKNYERAAWMKAFNCL 523
MA_121699g0010            DVSLSYLFLTNNLHYIVKKIKGSELLGLLGNRWLTKNEDKVRQYATNYEQAAWMKVLNCL 482
MA_5009135g0010           DISLSFLFLMNNLHYIVKKVKGSKLLELLGYGWLRENQDKVRHYAANYERVGWMKALNCL 280
MA_418978g0010            DPALSYVFLMNNGRYIVQKVKDSEIHSLLGDVWVRQHSSNVRRYHKSYQRVAWGKVLSCL 541
MA_130238g0010            DVSLSHIFIMNNVHYIVQKVKGSDLRALIGDDWLRKHSGKVRQYATNYQRATWMKVLSCL 613
MA_33877g0010             DIALSHLFLMNNVRYIVQKVRGSELKTLLGDDWIRKHSGQVRQYATHYQRSAWKKVLSCL 205
                                                ..   *:*  *: .:           ::  * . :

AtExo70E2                 RDNNDS----------------VRTLRERCRLFSLAFDDVYKNQTRWSVPDSELRDDLHI 583
AtExo70A1                 SAQGLTSSGGGSLEGGNSSGVSRGLLKERFKMFNMQFDELHQRQSQWTVPDTELRESLRL 608
MA_494970g0010            RDERIR------VNGGISTGVSEQALNDRYNGFNSEIEEVFRNHSGWIVPDVQLREEIRI 516
MA_12790g0040             RDEGTP------V-----SEMSQHALKDKFKGFNHGIEEVLRKHSGWMVPDVQLQGELRK 271
MA_22002g0010             RDEGIH------VSSGISSGMSRQALKDRFKRFNFAIEEVLRNHSGWMVPDVQLQEEITI 255
MA_29736g0010             RDEGIQ------VRGDFSSGVSQQVLEDRFKGFNFAFEEALRKHSGWMVPDLQLEEELRI 548
MA_89549g0010             RDEGIH------VSGDFSSGVSQQALEDRFKGFNFAIEEVLRKHSDWMVPDLQLQEELRL 589
MA_25591g0010             RDEGID------VSSSISSKMSQQALKDTLKRFNFAIEEVLRNHSGWIVPDVQLQEEITI 574
MA_138348g0010            RDEGIH------VSGGISSGVSQRALKDRFKGFNCAIEEVLRKHSAWMVPDVQLQEELRI 577
MA_121699g0010            KDEDIH------MNEGCSNEVSQQAVKDRFKGFNLAIEEALRKHSGWMVPDVQLQEELRH 536
MA_5009135g0010           RDEGID------VSSGISSKMSQQALKDTLKRFNFAIEEVLRNHSGWIVPDVQLQ----- 329
MA_418978g0010            RDEGIH------VGGNFSSGVSKPVLKERFKNFNALFEELQKTQSTWIVADDQLQTELRI 595
MA_130238g0010            RDEGIH------VGGSFSSAVSKTALKERFKSFNAAFEEVHRSQSTWLVPDPQLREELRI 667
MA_33877g0010             RDEGIY------IGGSSSTKVSKDALKDRFKSFNAVFEEVYRNHTAWFVPDPQLREELRI 259
                            :                      :.:  . *.  :::  : ::  * * * :*.

AtExo70E2                 STSVKVVQSYRGFLGRNA-----VRIGEKHIRYTCEDIENMLLDLFECLPSPRSLRSSRK 638
AtExo70A1                 AVAEVLLPAYRSFLKRFGPLVESGKNPQKYIKYTAEDLERLLGELFEGKSMNEPRR---- 664
MA_494970g0010            SIAEKIIPAYRFFLGRLRKYLESGRHSEMYIKYTSEHLETCLLNLFHGNTSSHGNLVGDT 576
MA_12790g0040             SITEKMIPTYRSFLGRFRRSLENGRHSEMYIKYTPDDLDAHLSHLFRAKSHHP------- 324
MA_22002g0010             SIAAKMIPTYRSFLGRLGKYLETGRHSEMYIKYTAEDLDAHLLDLFHGNPLSLSSRRF-- 313
MA_29736g0010             SIAEQMIPAYRSFLGRLRKYLESGRDSKMYIKYTPEDLETHLLD---------------- 592
MA_89549g0010             SIAEKMIPAYRSFLGRLRKYLESGRHSKMYIKYTPEDLETHLLDLFHSNP---------- 639
MA_25591g0010             SIAVKMIPAYRSFLGRLREYLETGRQSDMNIKYTAEDLETLILDLFHGNSLCLTSRGPNK 634
MA_138348g0010            SVAEKMIPTYRSVLGRLRKSSESGSHSQIYIKYTPGELEALLLDLFHDNSSSVSSSFLS- 636
MA_121699g0010            SIIEKMIPDYRSFLGRFSSYLESGRYSEMYMKYTTEDVEARLLDLFKVTHHRRGRS---- 592
MA_5009135g0010           ------------------------------------------------------------ 329
MA_418978g0010            SVAEMVIPAYRQFLGRFQYYLENDRHPERYIKYGPEEVEALINELFEGAPSSMTRRKS-- 653
MA_130238g0010            SIAEKLLPAYRSFFGRFKNHLESGRHSEMYIKYSPEDLEGAILDFFEGSPVSMHVRRRSN 727
MA_33877g0010             SIEEKLLPAYRSFMGRFRMHLEGVRHAEKYIKYSPEDLESYVLELFQGSPGSMHNSRRRI 319


AtExo70E2                 R----------------------------------------------------------- 639
```

```
AtExo70A1           ------------------------------------------------------------ 664
MA_494970g0010      LR---------CILSTRLNTW-----KLAS------------------------------ 592
MA_12790g0040       ------------------------------------------------------------ 324
MA_22002g0010       ------------------------------------------------------------ 313
MA_29736g0010       ------------------------------------------------------------ 592
MA_89549g0010       ------------------------------------------------------------ 639
MA_25591g0010       VSQSHEEEHGISHSSSKKDEDVVEEKEPESVQHDDGLLMCPHPSDESMQSSILPTQEEED 694
MA_138348g0010      SGRSEKEEEGDPKR------------KKESIDV--PLY---------------------- 660
MA_121699g0010      ----K---EGVC------------------------------------------------ 597
MA_5009135g0010     ------------------------------------------------------------ 329
MA_418978g0010      ------------------------------------------------------------ 653
MA_130238g0010      ------------------------------------------------------------ 727
MA_33877g0010       SS---------------------------------------------------------- 321


AtExo70E2           ----------------------           639
AtExo70A1           ----------------------           664
MA_494970g0010      ----------------------           592
MA_12790g0040       ----------------------           324
MA_22002g0010       ----------------------           313
MA_29736g0010       ----------------------           592
MA_89549g0010       ----------------------           639
MA_25591g0010       KASYSPFQDFDNTLFYDSESEG           716
MA_138348g0010      ----------------------           660
MA_121699g0010      ----------------------           597
MA_5009135g0010     ----------------------           329
MA_418978g0010      ----------------------           653
MA_130238g0010      ----------------------           727
MA_33877g0010       ----------------------           321
```

```
#  Percent Identity  Matrix - created by Clustal2.1
#
#

     1: AtExo70E2        100.00   27.79   28.06   30.20   36.17   28.20   27.44   25.60   28.88   28.38   34.98   30.78   30.13   41.10
     2: AtExo70A1         27.79  100.00   34.07   38.87   38.97   34.24   34.12   29.39   36.03   34.95   38.24   37.85   36.05   46.10
     3: MA_494970g0010    28.06   34.07  100.00   64.23   73.31   66.23   67.77   55.47   70.38   67.95   73.48   35.15   42.86   49.27
     4: MA_12790g0040     30.20   38.87   64.23  100.00   69.97   63.70   65.62   59.09   67.66   67.28   68.80   44.75   54.97   50.49
     5: MA_22002g0010     36.17   38.97   73.31   69.97  100.00   68.29   74.75   75.77   74.66   70.42   84.80   44.09   58.52   49.35
     6: MA_29736g0010     28.20   34.24   66.23   63.70   68.29  100.00   77.09   62.40   74.02   71.77   66.67   36.77   45.57   47.77
     7: MA_89549g0010     27.44   34.12   67.77   65.62   74.75   77.09  100.00   57.87   74.84   71.97   72.64   38.54   46.03   51.47
     8: MA_25591g0010     25.60   29.39   55.47   59.09   75.77   62.40   57.87  100.00   60.34   65.42   87.36   30.65   36.39   43.18
     9: MA_138348g0010    28.88   36.03   70.38   67.66   74.66   74.02   74.84   60.34  100.00   74.59   77.92   39.15   44.35   51.32
    10: MA_121699g0010    28.38   34.95   67.95   67.28   70.42   71.77   71.97   65.42   74.59  100.00   74.47   37.70   48.05   49.84
    11: MA_5009135g0010   34.98   38.24   73.48   68.80   84.80   66.67   72.64   87.36   77.92   74.47  100.00   40.73   53.82   48.03
    12: MA_418978g0010    30.78   37.85   35.15   44.75   44.09   36.77   38.54   30.65   39.15   37.70   40.73  100.00   46.08   53.63
    13: MA_130238g0010    30.13   36.05   42.86   54.97   58.52   45.57   46.03   36.39   44.35   48.05   53.82   46.08  100.00   63.32
    14: MA_33877g0010     41.10   46.10   49.27   50.49   49.35   47.77   51.47   43.18   51.32   49.84   48.03   53.63   63.32  100.00
```

Let's align the short spruce sequences with the Arabidopsis ones:

```
CLUSTAL O(1.2.4) multiple sequence alignment


AtExo70A1           MAVDSRMDLLSERAVLMRASLQKSQTITDNVVSILGSFDSRLSALETAM---RPTQIRTH 57
MA_9976656g0010     ------------------------------------------------------------ 0
AtExo70E2           ----------------------------------MAEFDSKVPVSGMNNHVFEACHHVVK 26
MA_8703090g0010     ------------------------------------------------------------ 0
MA_129851g0010      ------------------------------------------------------------ 0


AtExo70A1           AIRKAHENIDRT----LKAAEVILSQFDLLRQAETKVLKGPHEDL-ESYLDAIAQLRKII 112
MA_9976656g0010     ------------------------------------------------------------ 0
AtExo70E2           ALRASDNNLDANLRKLLSDLEMHLSTFGIA---DTKVEDAGFSEIKKRFKEAV----KRI 79
MA_8703090g0010     ------------------------------------------------------------ 0
MA_129851g0010      ------------------------------------------------------------ 0


AtExo70A1           RYFMSNKSFKSSDGVLNHANSLLAKAQSKLEEEFKQLLASYRILRMT-------FY---- 161
MA_9976656g0010     ------------------------------------------------------------ 0
AtExo70E2           RSWETNQSTMFEAGLSE-ADQFFQA-----LYDVQTVLVGFKALPMKTNQMEKDVYNQAT 133
MA_8703090g0010     ------------------------------------------------------------ 0
MA_129851g0010      ------------------------------------------------------------ 0


AtExo70A1           --LNYRSCHLTKIFFCEIKH--FKAVEPDRL----------FDGLPNSLRPSSDGDGGGK 207
MA_9976656g0010     ------------------------------------------------------------ 0
AtExo70E2           VALDIAMLRL-EKELCDVLHQHKRHVQPDYLAVSSRRKDIVYDESFVSL------DDEVI 186
MA_8703090g0010     ------------------------------------------------------------ 0
MA_129851g0010      ------------------------------------------------------------ 0
```

```
AtExo70A1           PHGGHHNDDAETAAYTLPILIPSRVLPLLHDLAQQMVQAGHQQQLLQIYRDTRSFVLEES 267
MA_9976656g0010     ------------------------------------------------------------ 0
AtExo70E2           VEASSHEDDEQISDFYNSDLVDPIVLPHIKAIANAMFACEYDQPFCEAFIGVQREALEEY 246
MA_8703090g0010     ------------------------------------------------------------ 0
MA_129851g0010      ------------------------------------------------------------ 0

AtExo70A1           LKKLGVEKLSKEDVQRMQWEVLEAKIGNWIHFMRIAVKLLFAGERQVCDQIFRGFDSLSD 327
MA_9976656g0010     ------------------------------------------------------------ 0
AtExo70E2           MVTLEMERFSCVDVLRMDWEDLNGAMRKWTKVVKIITQVYLASEKQLCDQILGDFESIST 306
MA_8703090g0010     ------------------------------------------------------------ 0
MA_129851g0010      ------------------------------------------------------------ 0

AtExo70A1           QCFAEVTVSSVSMLLSFGDAIARSKRSPEKLFVLLDMYEIMRELHTEIETIFKGKACLEI 387
MA_9976656g0010     ------------------------------------------------------------ 0
AtExo70E2           ACFIEISKDAILSLLNFGEAVVLRSCKPEMLERFLSMYEVSAEILVDVDNLFPDETGSSL 366
MA_8703090g0010     ------------------------------------------------------------ 0
MA_129851g0010      ------------------------------------------------------------ 0

AtExo70A1           RDSATGLTKRLAQTAQETFGDFEEAVEKDATKTAVLDGTVHPLTSYVINYVKFLFDYQTT 447
MA_9976656g0010     ------------------------------------------------------------ 0
AtExo70E2           RIAFHNLSKKLADHTTTTFLKFKDAIASDESTRPFHGGGIHHLTRYVMNYLKLLPEYTDS 426
MA_8703090g0010     ------------------------------------------------------------ 0
MA_129851g0010      ------------------------------------------MTITANAPIGISKDLPDG 18

AtExo70A1           LKQLFLE--------FGNGDDSNSQL----ASVTMRIMQALQNNLDGKSKQYKDPALTHL 495
MA_9976656g0010     ------------------------------------------------------------ 0
AtExo70E2           LNSLLQNIHVDDSIPEKTGEDVLPSTFSPMARHLRSIVTTLESSLERKAQLYADEALKSI 486
MA_8703090g0010     ---------------------------------------------------YKDPALSHL 9
MA_129851g0010      LVDFSRD--------LGEQDNELGSLATALSVRLGWIIISLQSKLDVKSDLYQDVSLSYL 70

AtExo70A1           FLMNNIHYMVRSVRRSEAKDLLGDDWVQRHRRIVQQHANQYKRVAWTKILQSSSAQGLTS 555
MA_9976656g0010     ------------------------------------------------------------ 0
AtExo70E2           FLMNNFRYMVQKVKGSELRRLFGDEWIRKHIASYQCNVTNYERSTWSSILALLRDNN--- 543
MA_8703090g0010     FLMNNGRYIVHKVKDTEIGSLLGNDWVRKHAAKVRQYHKNYQRTAWGKVLACLKDGG--- 66
MA_129851g0010      FLMNNLHYIVQKVKSSKLLGLLGYGWLRKNRCKAREYAENYERAAWTKTLNCLRDEG--- 127

AtExo70A1           SGGGSLEGGNSSGVSRGLLKERFKMFNMQFDELHQRQSQWTVPDTELRESLRLAVAEVLL 615
MA_9976656g0010     ----------------------------------------------------ISVAEKMI 8
AtExo70E2           -------------DSVRTLRERCRLFSLAFDDVYKNQTRWSVPDSELRDDLHISTSVKVV 590
MA_8703090g0010     -------------IH--------------------------------------------- 68
MA_129851g0010      -------------IRVGGDFSS-GVSQQALEDRFX------------------------- 148

AtExo70A1           PAYRSFLKRFGPLVESGKNPQKYIKYTAEDLERLLGELFEGKSMNEPRR-----       664
MA_9976656g0010     PAYRSFLGRLRKYSESGRHSQINIKYTAEDLEARLLDLFHGNSS----------       52
AtExo70E2           QSYRGFLGRNAV-----RIGEKHIRYTCEDIENMLLDLFECLPSPRSLRSSRKR       639
MA_8703090g0010     ------------------------------------------------------       68
MA_129851g0010      ------------------------------------------------------       148


#  Percent Identity  Matrix - created by Clustal2.1
#
#

     1: AtExo70A1        100.00   55.77   30.56   48.53   29.17
     2: MA_9976656g0010   55.77  100.00   46.81    -nan    -nan
     3: AtExo70E2         30.56   46.81  100.00   45.59   30.41
     4: MA_8703090g0010   48.53    -nan   45.59  100.00   54.41
     5: MA_129851g0010    29.17    -nan   30.41   54.41  100.00
```

Spruce sequences as obtained from the ConGenIE (in plantgenie.org)

>MA_10428691g0010

MDYMSSALIFSGLQDFSVLTNSEAFEQSVDLIILIIYTSMVIQEAAVGGGDRVGGSSENMTRDMFPILTFD
NRLSATSHQNPGNPHGKSADLEANLDYADRIISCRDSNSFNPILFQSNPKETNEYLDAVDGLRLLMESL
VVTHSSSDKMVRAQRLMEIEKRLCDEIFSGLHSVRDFCFAELVKGPTTRLLAFGESVAMSKKSSERLFRVL
DMYETLSDLMPDIDTVYCHESCASVRTQASTILVRIGESARGILREFENAIQAENSKTPVPGGTIHPLTAYV
MNYLIFLSDYKETLMNITANGPTDMSKDLPNSLMDLFGELDGHDDELGSVASALSVKLGWIIISLQSKLD
VKSNLYKDVSLSYLFLMNNLHYIVKKVKGSKLLGLLGYGWLRKNQGRVRQYAENYERAAWMKALNCLR
DEGIHVRGNFSSGLSQQVLEDRFKGFNFAIEEALRKHSGWMVPDLQLEEELRISIAEQMIPAYRSFLGRLR
KYLKSGSQSKMYIKYSPEDLETHLLDLFHSNPSSVSSRRSEISSRRSEKEAE

>MA_10431828g0010

MASEDGEEKVIATAQHIVKALGTTETMTDDMIHIFSKFDHRFSGMSEGLSRKSAESYDSEGLQDALDSV
EERIMRWDMATSESARERFIWSDVPSEASSYLEAVDDLQNMIAKLSLAPDDRKEFACLDRAQNVLQQA
MARLEEEFRMLLEKHSESVDPDWLFDPISQPSFSSSNNDDKASHTSAEDEGDADDLPVAQPVTDLNFTI
DMLPSEVVADLNRIAKCMIGAGYMAECCHVYVTVRRMFLEESVFRLGFEKQSIEEVQKMGWDILEVEIS
KWMQVMKVTVHVFFTSERKLCERVFVDLPSVAVTCFVELARGVMVQLLNFAEAVVISRRSPERLFKILD
MYETLRDLLPEINSLFSGEDGFELRSEASTIWMRLAEAARGTFVDGVMVQPLNFAGAVVISRRSPERLFKI
LDMYKTLRDLLPEINSLFSGEDGFELRSEASTVRMRLAEAARGTFVEFENAIQRDSAKNPVPGGAVHPLT
RGEDPRVFDGAVNCAGSVSPDNRKNSADGLSPWSIQTIWLMELLEGNLDAKSKLYKDPALSHLFLMN
NGRYIVHKVKDTEIGSLFSNDWVRKHA

>MA_121699g0010

MLFQSSRKEVYEYLSAVDSLRQLMENLVVTPSSSAKLVRAQRLMEMSMPRLQKEFQRILSANVEPTLAP
DDGNLSRGSSDDDRGSSDDESTDQVGNSGGRRRTRNCNADEICEIDMMPLDAVLDLRSIAKRIVAGG
YGSECIRIYGLTRKSVIKRCLNRIGVEWLNTSDVQKMEWAVLDIKIKNWIRAARIAVRILFASEKRLCDEIFA
GLNHVSEKRLCDEIFAGLNHVRDSCFTEVVKGPTTKLLAFGESVAMSRKSSEKLFRVLDMYETLSDLMPD
IDTVYCQESCASVRAQASTIFVRLGESAWGILTEFENAIQAENSKTPVPGGTIHPLARYVMNYLSFLCDYK
EALMNITANAPIAIPKALPVGLVGSSGDLVGHDDELGSPDELGSPASVLRVRVGWIITNLQGKLELKSNLY
KDVSLSYLFLTNNLHYIVKKIKGSELLGLLGNRWLTKNEDKVRQYATNYEQAAWMKVLNCLKDEDIHMN
EGCSNEVSQQAVKDRFKGFNLAIEEALRKHSGWMVPDVQLQEELRHSIIEKMIPDYRSFLGRFSSYLESG
RYSEMYMKYTTEDVEARLLDLFKVTHHRRGRSKEGVC

>MA_12790g0040

MGKKSSDRLFGVLDMYDCLSKLMPDIDTVYSQKECVGVRTKASAVLVRLGEAARGIFIEFENAVHGENS
KTRIPGGTIHPLTRYVMNYLSFMSDYKEALVKITAHVPMDIPRALPNGLMGLFEDQDWDVEEQGRPASV
LSVRLGWIIIILQCKLDTTSKLYKDVALSYLFLMNNLNYIVKKIKDSELLGLLGYGWLTKNQGKVRQYAANY
ERGAWMKVLNCLRDEGTPVSEMSQHALKDKFKGFNHGIEEVLRKHSGWMVPDVQLQGELRKSITEK
MIPTYRSFLGRFRRSLENGRHSEMYIKYTPDDLDAHLSHLFRAKSHHP

>MA_130238g0010

MDQPGDEKKMAEAHSRNNSVVNSECPSEDQGSLDENSECLSQSFVVLDENLDNAEKLILKWDMASS
EAAKQSMIFEGQPEEAEKYLKTVDELQKLMESVSLSQADVHKAQNVLQIAMVRLEEEFRNLLLVHSEPL
DPELVLNPASKASFRSLNESFGGQPEEAEKYLKTVDELQKLMESVSLSQADVHKAQNVLQIAMVRLEEE
FRNLLLVHSEPLDPELVLNPASKASFRSLNESFGDRPSHSSDDDEAEEEEEEAVSHPLADKICEMDLIPQD
VIPDLHRIAERMVSAGYTRECMQVYGSIRKSMVDESFLRLGVEKLSIGDVQRLGWETLEVKIKKWIRAAK
VCIRMLFASEKHLSEQVFDGLDSVGETCFSELAKGATMQLFNFSEAIAISRRSPEKLFKILDLYETLSELLPDI
EIVFSNEMCANVKTQASEILTRLGEAARGIFTEFENAIQRETSRTPIPGGTIHPITRYVMNYISFISDYKETLM
KLIVNKPSQTSNLLPEGLIDLIGEYDRASDSDNQSVLSLHLIWITLILQNNLEGKSKVYKDVSLSHIFIMNN
VHYIVQKVKGSDLRALIGDDWLRKHSGKVRQYATNYQRATWMKVLSCLRDEGIHVGGSFSSAVSKTAL
KERFKSFNAAFEEVHRSQSTWLVPDPQLREELRISIAEKLLPAYRSFFGRFKNHLESGRHSEMYIKYSPEDL
EGAILDFFEGSPVSMHVRRRSN

>MA_138348g0010

MEIEEVAAHQGGEDRVVAMVHHIVTSLSTSENMTNDMLTMLSTFDNRPSTMSHQCPTNPPRKSADLE
SNLASADRIISFWESNSAKAMLFQSSRKEANEYLTAVDGLRHLMENMVLVHSSSAKMVRAQRLMEMS
MARLEKEFHRILSANVKPTIAPDDSNSNHGSSDDGSTDEDVSSSGHRRSRNSNAEEICEIDMMPLDAVL
DLRSIAKRMVSGGYGSECVRIYGQTRKSVIKECLNRIGVERLSTSDVQKMEWEILDFKIKNWIRSVRIGVRI
LMASEKRLCDEIFSGLNNVRDSCFAEVVKGPTTKLLAFGESVAMSKKSSERLFRVLDMYETLWDLMPDID
TVYCQDSCASVRTQASTIFVRLRDSALGIMTTFENAIQAENSKTPVPGGTIHPLTGYVMNYLSFLSDYKET
LMNMMASATIDGQDDELDSRASALSTRLGWIIINLQCNLDLKSNLYKDVALSNLFLMNNLHYIVNKVK
GSKLLGLLGYGWLRQNQSEVREYAKNYERAAWMKAFNCLRDEGIHVSGGISSGVSQRALKDRFKGFNC
AIEEVLRKHSAWMVPDVQLQEELRISVAEKMIPTYRSVLGRLRKSSESGSHSQIYIKYTPGELEALLLDLFH
DNSSSVSSSFLSSGRSEKEEEGDPKRKKESIDVPLY

>MA_22002g0010

MYETLSELMPDFDIVYCQKSCASVRTQVSTIVVQLGESALGILTEFENAVQVENSKTPVAGGTIHPLTRYV
MNYLSFLSDYKEALMKIIANAPIDIPKALPDGLMGLFGDLDGPASDLSVKLGCIIIILQCKLDLKSNVYEDV
SLSYLFLMNNFHYIVKKVKNSKLLGLLGYGWLRKNQDNVGHYAANYERAAWMKALNCLRDEGIHVSS
GISSGMSRQALKDRFKRFNFAIEEVLRNHSGWMVPDVQLQEEITISIAAKMIPTYRSFLGRLGKYLETGRH
SEMYIKYTAEDLDAHLLDLFHGNPLSLSSRRF

>MA_25591g0010

MLRMYALIQGTFSEGIKEFTNGGFTMPIGWKIEKATADQGGAKEKSSGHGTSYCEISRHLREHDERHAHS
PLHLRQPPFRHETSKSLQPTWKIGRFGYQFCLRRPKNLVLGLEPVQTLDPVPEQSKRSQRVSCLLGWSPS
PLENLVVTHNSSAKLVRAQRLMEVWMARLKIEFQQILSANVEPTMVPDDSDSSHASSDDESTDEEGSS
GGHRRSRNSNADEICEIDMMSLEAVLDLRSIAKRMVAAGHGRECFRIYEKTRKSVIEGCLNRIGVDRLST
SDVQKMEWDILDIKIKKWIRAVRIVVRILFASEKLLCDEIFAGINNVRDSCFTEVVKGPTTKLLAFGESVAM
SKTVSERLFRVLDMYEILSDLMPDLDIVYCQKSWASLRTHVSMNVVQLGESALGILRNSRMQSRWRIQI
HRSPALRFIRLQGLATIHPLTRYVMNYLSFLSDYKEVLMNIIANAPIDIAKALREGVMGLVKGSKLLELLGY
RWLRENQDKVKHYAANYERVAWMKALNCLRDEGIDVSSSISSKMSQQALKDTLKRFNFAIEEVLRNHS
GWIVPDVQLQEEITISIAVKMIPAYRSFLGRLREYLETGRQSDMNIKYTAEDLETLILDLFHGNSLCLTSRGP

NKVSQSHEEEHGISHSSSKKDEDVVEEKEPESVQHDDGLLMCPHPSDESMQSSILPTQEEEDKASYSPF
QDFDNTLFYDSESEG

>MA_29736g0010

MLTILTFDNCLSPTSHQNPGKLHGKSADLEANLAFAERIISSSDSNSFNSILFQSNPKETNEYLDAVDGLR
HVMESLVVTHSSSDKMVRAQRLMEMSMARLQKEFHRILSANVEPTMAPVDGNSREASSDDGSTDED
ESSSGRRQSWNCNPDEICKIDMMCVEAVLDLRSIAKRMVAGGYGSECVRIYGLTRKSVIEGCLNRIGVER
LSTSDVQKMEWGILDIKIKKWIRAVRIAVRILFASEKREKRFCDEIFSGLHSVSDFCFAELVKGPTTKLLVFGE
SVALSKKSSERLFRVLDMYETLWDLIPDIDTVYSQESCASVRTQASTILVRLGESARGILREFENAILAENSK
TPVPGGTIHPLTGYVMNYLIFLSDYKETLMNITANAPVDISKDLPDTLMDLFGELDGHGDDLGSVASAVS
VRLGWIIISLQKGQRFEASRAARVWLVEEESGQELLGLLGYGWLRKNQGRMRQYAENYEREAWMKALN
CLRDEGIQVRGDFSSGVSQQVLEDRFKGFNFAFEEALRKHSGWMVPDLQLEEELRISIAEQMIPAYRSFL
GRLRKYLESGRDSKMYIKYTPEDLETHLLD

>MA_33877g0010

MYESLSDLLPVIKSIVLGKSCTSIRAEADGILTRLGEAAKGTFVEFENAIQRETSKTPIPGGAIHPLTRYVMN
YIKFLFDYTDTLQQLIDEKKEAIPRQLSDEFTEFPEEDRHGDRSNTTPLARRLLSITSLLESNLEGKSKLYKDI
ALSHLFLMNNVRYIVQKVRGSELKTLLGDDWIRKHSGQVRQYATHYQRSAWKKVLSCLRDEGIYIGGSS
STKVSKDALKDRFKSFNAVFEEVYRNHTAWFVPDPQLREELRISIEEKLLPAYRSFMGRFRMHLEGVRHA
EKYIKYSPEDLESYVLELFQGSPGSMHNSRRRISS

>MA_418978g0010

MASPEEDDTSESVVETLADDMHRIFSKLDGPFSCIDGANNRKNEEFVLPVNLEEELSSLEDKIMKWEGP
GFMVAGEGSNSSEETERVYSYLRAVDDVQSLVNGFSQLPDDVKDFTVLQRIEAIFHQAMDCLEEEFRAL
LEKHSNAVDPDCLFDAASGPSFNSSCDERAFRNLADDEKEEDVPVAQPVGDLNFTIDSLPSEIVNRLSE
IAVRMVRAGYRTECCQVYCMIRKMFLEESVMRLGFEKLSIEEVQRIQWELLEVEISKWMQTLKVSVNVF
MSSERILCDRVFVDLPSVSETCFGELARGVMIQFLNFGEAVAISRRSPEKLFKILDMYETLWELIPKINSIFS
GEVCSTLRSEASAVWLRLGEAARGTFLELENAIKGDLAKNPVPGGAIHPLTRYVMNYMRLACDYRDTLE
QVFKEDGGAEMNGVSARALDHSDVLEFRNGTNTTDSSPLSTKTIAIMELLESNLNAKSKLYKDPALSYVF
LMNNGRYIVQKVKDSEIHSLLGDVWVRQHSSNVRRYHKSYQRVAWGKVLSCLRDEGIHVGGNFSSGV
SKPVLKERFKNFNALFEELQKTQSTWIVADDQLQTELRISVAEMVIPAYRQFLGRFQYYLENDRHPERYIK
YGPEEVEALINELFEGAPSSMTRRKS

>MA_494970g0010

MLTVPSTFDSHTSARSHQNPGNPPGKSADFETNLAFADRIISFWDSHSSECMLFQSSRKETNEYLAAVD
GLRDLMEILVVTQSRSADLVRAHRLMEMSMACLQKEFHRILTVNVEGTMAPADANSSHESTDEEGSRG
ASCRSRSSNADKISSCRSRSSNADKICEIDLMPLDAVLDLRNIAKRMVAGGYWRDCVRIYGLTRKSVME
GCLNRIGVGRLSATDVKKMEWGILDMKIKNWIGAVSIAVRILFASEKQLCDEIFAGLNHVRDSCFAEVVK
EPTTKLLDFGESVAMNQKGSERLFREPCAGVRTQASRIFVRLGKSAIGIMTVFETAIHKENSKTSVPGGTIH
PLTRALPDGLMGMSGDLDGHDSELGSPASALSVKLRWIIIILQCKLDLKSNLYKDVALSNLFLMNNLHYI

VKKVKGSKLLGLLGYGWLRKNQDNVRHYAANSERAAWMKALDSLRDERIRVNGGISTGVSEQALNDR
YNGFNSEIEEVFRNHSGWIVPDVQLREEIRISIAEKIIPAYRFFLGRLRKYLESGRHSEMYIKYTSEHLETCLL
NLFHGNTSSHGNLVGDTLRCILSTRLNTWKLAS

>MA_5009135g0010

KIKKWIRAVRIVVRILFASEKRLCDEIFAGIINVRDSCFTEVVKGPTTKLLAFGESVAMSKKVSERLFRVLDM
YETLSDLMPDFDIVYGQKSCASVRTQVSMNVVRLGESALGILTEFENAVQVENSNTPVAGGTIHPLTRYV
MNYLSFLSDYKEVLMNIIANAPIDIAKALPEGVMGLSEDLERSDNELGSPASDLSVKLGRIIFILQCKLDLK
SILYEDISLSFLFLMNNLHYIVKKVKGSKLLELLGYGWLRENQDKVRHYAANYERVGWMKALNCLRDEGI
DVSSGISSKMSQQALKDTLKRFNFAIEEVLRNHSGWIVPDVQLQ

>MA_89549g0010

MEIQEEAAGEDGVVATTHHIDKSLGSSEDMTRDMLTTLTFDNGLSATSHQNPERNPHEKSTDLEANLAS
ADRIISLWDSKSFNSMLFQSNPKEASEYLDAVDGLRHLMETLVVVHSSSAMLVRAQRLMEMSMVRLEK
EFHRILSANVEPSMAPDDGNSSHASSHEGLIDEDERSSGHRQIWNSKADEICEIDMMSVESVLDLRSIA
KRMVAGGYGSECVRIYGLTRKSYEQRPEDRMGNSRHWLSTSNVQKIEWGILDINIQKWIRVVRIAVRILF
ASEKRLCDKIFAGLNNVSEFCFAEVVKGPTMKLLAFGEIVAMSKMSSERLFRVLDMYETLWDLMPNIDT
VYCQESCASVKTQASTILVRLGESAWGILTDFENAIQAENSKTPVPGGTIHPLTGYVTNYLIFLSDYKETLM
NIMVNSQIGISKDVPDDLVDLSRKDDELGSLAFALSVRLGWIIISLQCKLHMKSNLYQDVSLSYLFLMNN
LHYIVKKVKGSKLLGLFGYGWLRKNQCKVRKYAENYERAAWTKALDCLRDEGIHVSGDFSSGVSQQALE
DRFKGFNFAIEEVLRKHSDWMVPDLQLQEELRLSIAEKMIPAYRSFLGRLRKYLESGRHSKMYIKYTPEDL
ETHLLDLFHSNP

>MA_8703090g0010

YKDPALSHLFLMNNGRYIVHKVKDTEIGSLLGNDWVRKHAAKVRQYHKNYQRTAWGKVLACLKDGGI
H

>MA_9976656g0010

ISVAEKMIPAYRSFLGRLRKYSESGRHSQINIKYTAEDLEARLLDLFHGNSS

>MA_129851g0010

MTITANAPIGISKDLPDGLVDFSRDLGEQDNELGSLATALSVRLGWIIISLQSKLDVKSDLYQDVSLSYLFL
MNNLHYIVQKVKSSKLLGLLGYGWLRKNRCKAREYAENYERAAWTKTLNCLRDEGIRVGGDFSSGVSQ
QALEDRFX

29-Apr-2024
Dr. Anna Karkonen
Natural Resources Institute Finland
Helsinki 00790
Finland

MSID: PP2023-RA-02044R1
MS TITLE: Extracellular vesicles of Norway spruce contain precursors and enzymes for lignin formation, and salicylic acid

Dear Dr. Anna Karkonen:

I am pleased to inform you that your revised manuscript "Extracellular vesicles of Norway spruce contain precursors and enzymes for lignin formation, and salicylic acid" has been accepted for publication in Plant Physiology. Your manuscript will be checked for consistency with journal formatting and image standards by one of our Science Editors, who will contact you shortly. They may have a number of queries and suggestions for you. Please keep in mind that they work to increase the online visibility of your article and its attraction for readers from the widest of backgrounds. You will have the opportunity to review any revisions, and I hope you will work constructively with them to get the most out of your research. You will also have the opportunity to review all changes when you receive the author proofs for your article.

Once validated for publication, your article will be sent to a Science Editor for a final review of your manuscript and source files. After your final files are uploaded, your article will appear in the next available issue and the online Preview version of your article will be posted after your signed Copyright and Checklist forms and final source files are received.

Please be sure to include the following in your manuscript file:
Harvard style citations
List of all author contributions and funding information at the beginning of manuscript after the titles and author list.
List all supplemental materials associated with the article, with titles, at the end of the ms, before the references and acknowledgments section.
Complete Figure Legends for all figures appearing in the article listed before the Literature Cited.

Thank you and congratulations on your Plant Physiology paper!

Sincerely,
Editor
Plant Physiology

=====
FIRST AUTHOR PROFILES
=====

We recognize the hard work that goes into being First Author of a Plant Physiology paper by publishing a "First Author Feature" for each First Author alongside your paper.

If the first author(s) would like to submit information for a feature on Facebook and Twitter, fill out the form by clicking on the link below:

Plant Physiology First Author Feature Form: https://forms.office.com/r/ZBCGZfhm8F
If you cannot access the form through the link above, please contact Rachel at rbelsky@aspb.org.

To upload an image of yourself to accompany your profile, please put your photo in the correct journal folder at the link below. You must label your image: Author First Name Last Name Article Title.

First Author Profile Image Dropbox: https://www.dropbox.com/scl/fo/xz029p7qjr2jml2rp2kmq/h?dl=0&rlkey=6fkxcaqmxx35ap152b2osy1fz

=====
IMPORTANT REMINDER: PEER REVIEW REPORTS
=====

If you opted to publish a peer review report along with your article during the original submission process, it will be prepared by the editorial staff and publicly posted with your manuscript, inside the zip file that contains any other supplemental material. As a reminder, the peer review report is a public record of all comments from editors and reviewers, as well as your prior responses, as you received them in the decision letters for each draft of your manuscript. If you agreed to publish this report and have changed your mind, or are not sure if you selected this option, please contact the editorial office as soon as possible before signing the license agreement from our publisher.

-- Reviewer comments:
Reviewer #1 (Comments for the Author):

The authors have done a balanced job of addressing the concerns of the reviewers. The new Table 1 really adds to the work, and I appreciate the challenges of the spruce genome more fully.

Reviewer #2 (Comments for the Author):

The authors have addressed all my concerns
